# Supplementary figures and images for: Fast polypharmacy side effect prediction using tensor factorization (part 2 of 2)
Source: Bioinformatics. 2024 Nov 25;40(12):btae706. doi: 10.1093/bioinformatics/btae706 (PMC11646082; doi:10.1093/bioinformatics/btae706)

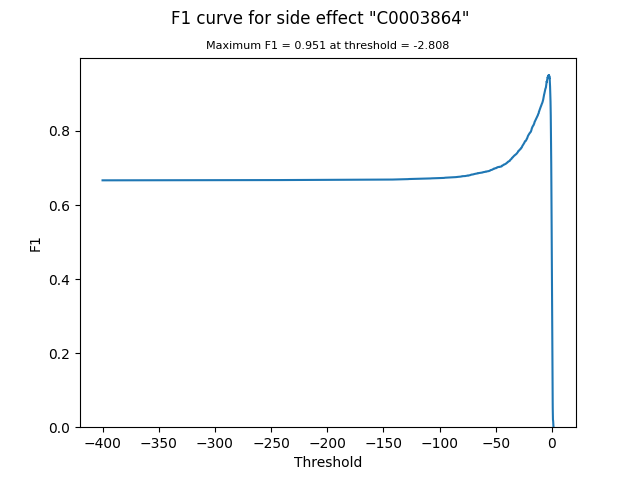

Supplement: btae706_Supplementary_Data [file btae706_supplementary_data.zip › simple_selfloops/figures/C0003864/F1_curve.png]

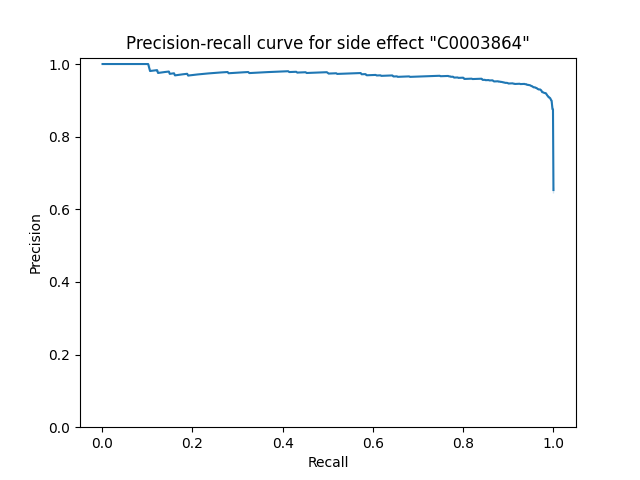

Supplement: btae706_Supplementary_Data [file btae706_supplementary_data.zip › simple_selfloops/figures/C0003864/precision_recall.png]

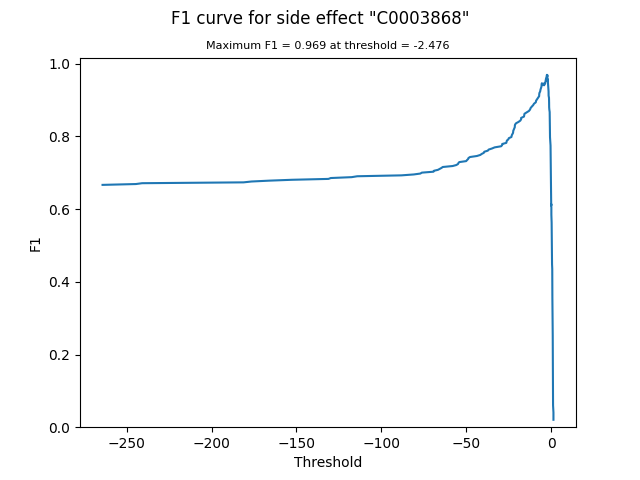

Supplement: btae706_Supplementary_Data [file btae706_supplementary_data.zip › simple_selfloops/figures/C0003868/F1_curve.png]

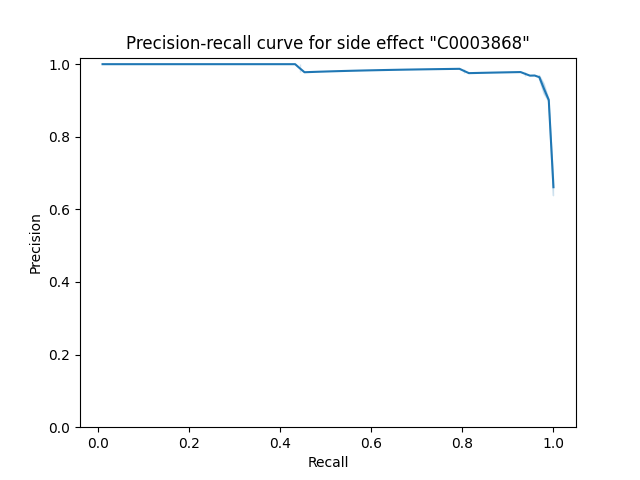

Supplement: btae706_Supplementary_Data [file btae706_supplementary_data.zip › simple_selfloops/figures/C0003868/precision_recall.png]

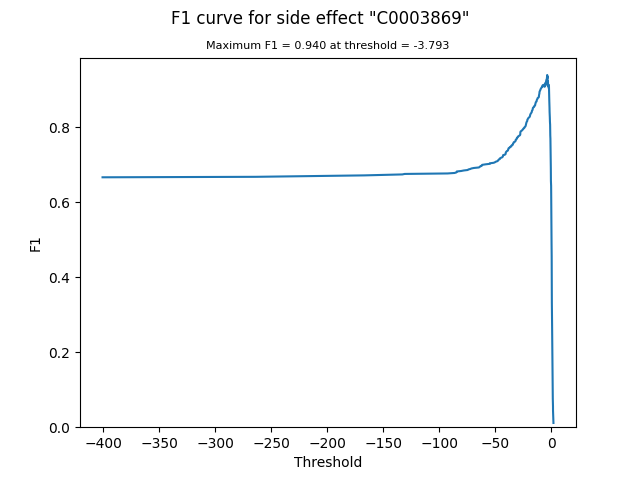

Supplement: btae706_Supplementary_Data [file btae706_supplementary_data.zip › simple_selfloops/figures/C0003869/F1_curve.png]

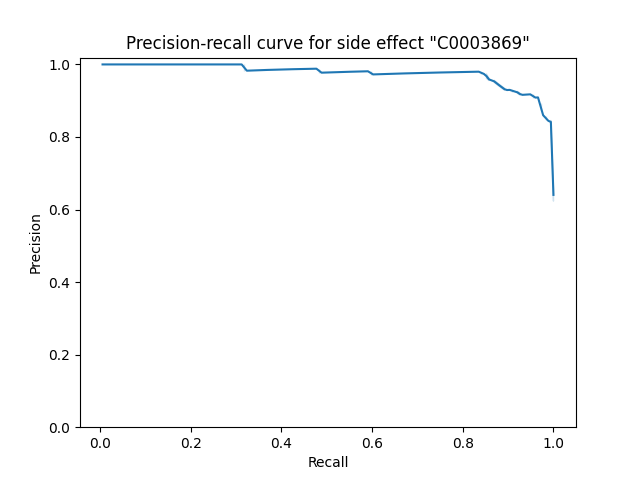

Supplement: btae706_Supplementary_Data [file btae706_supplementary_data.zip › simple_selfloops/figures/C0003869/precision_recall.png]

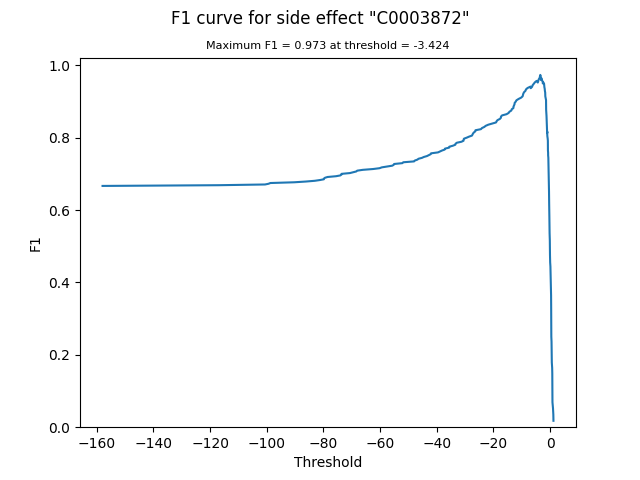

Supplement: btae706_Supplementary_Data [file btae706_supplementary_data.zip › simple_selfloops/figures/C0003872/F1_curve.png]

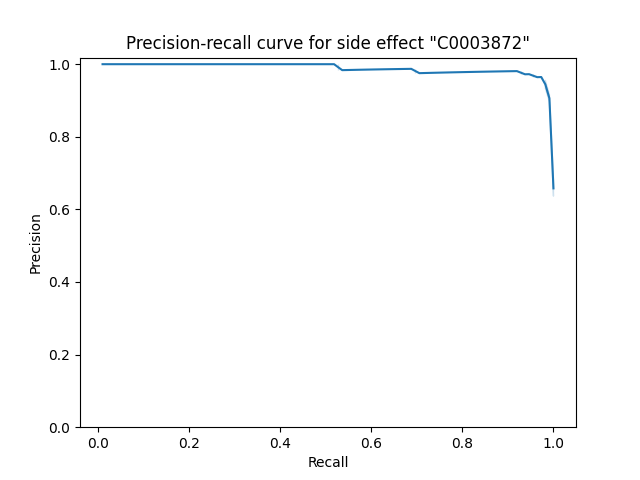

Supplement: btae706_Supplementary_Data [file btae706_supplementary_data.zip › simple_selfloops/figures/C0003872/precision_recall.png]

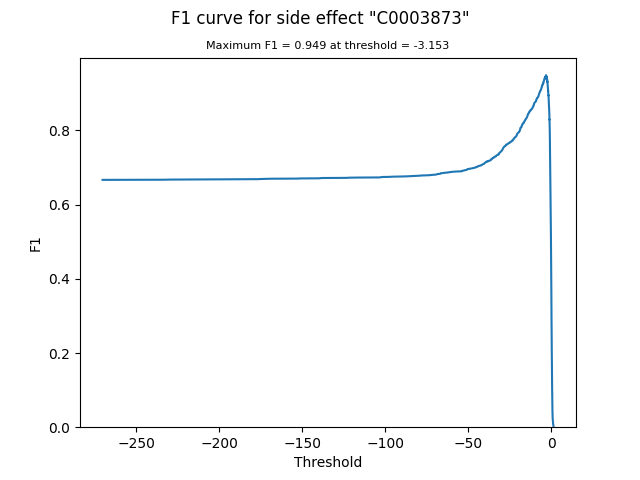

Supplement: btae706_Supplementary_Data [file btae706_supplementary_data.zip › simple_selfloops/figures/C0003873/F1_curve.png]

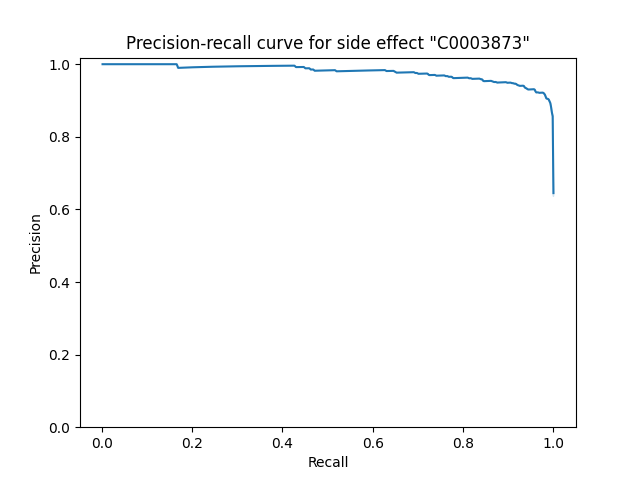

Supplement: btae706_Supplementary_Data [file btae706_supplementary_data.zip › simple_selfloops/figures/C0003873/precision_recall.png]

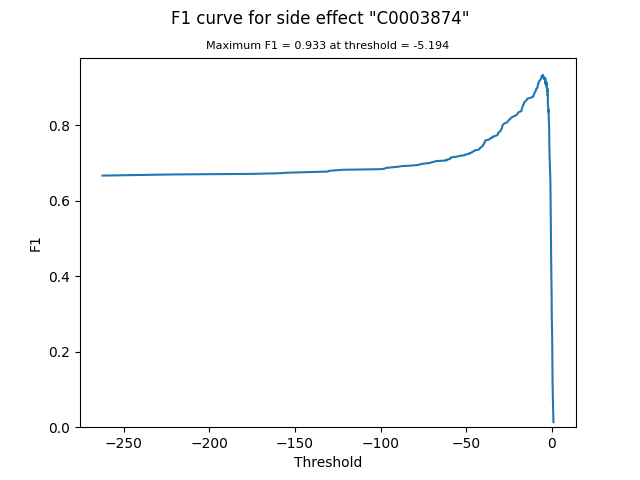

Supplement: btae706_Supplementary_Data [file btae706_supplementary_data.zip › simple_selfloops/figures/C0003874/F1_curve.png]

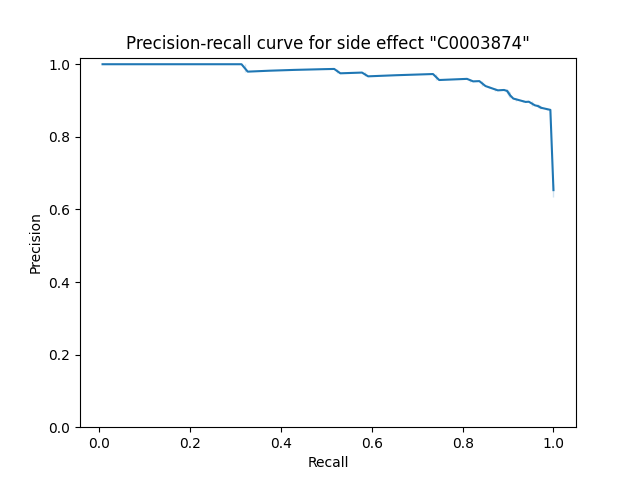

Supplement: btae706_Supplementary_Data [file btae706_supplementary_data.zip › simple_selfloops/figures/C0003874/precision_recall.png]

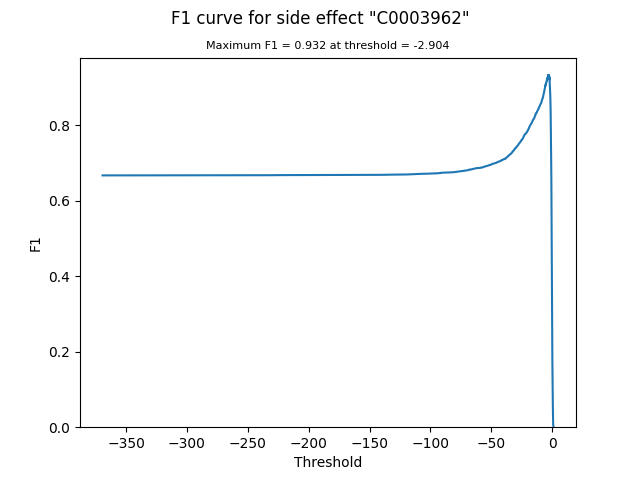

Supplement: btae706_Supplementary_Data [file btae706_supplementary_data.zip › simple_selfloops/figures/C0003962/F1_curve.png]

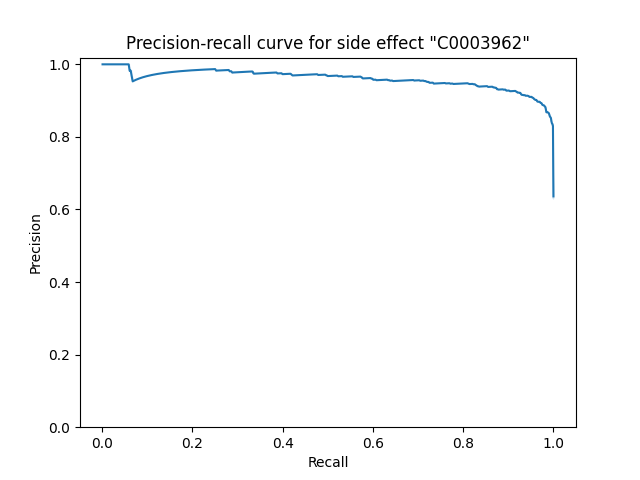

Supplement: btae706_Supplementary_Data [file btae706_supplementary_data.zip › simple_selfloops/figures/C0003962/precision_recall.png]

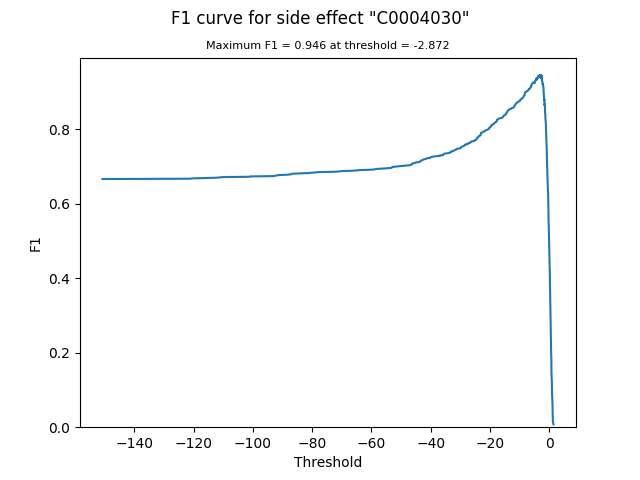

Supplement: btae706_Supplementary_Data [file btae706_supplementary_data.zip › simple_selfloops/figures/C0004030/F1_curve.png]

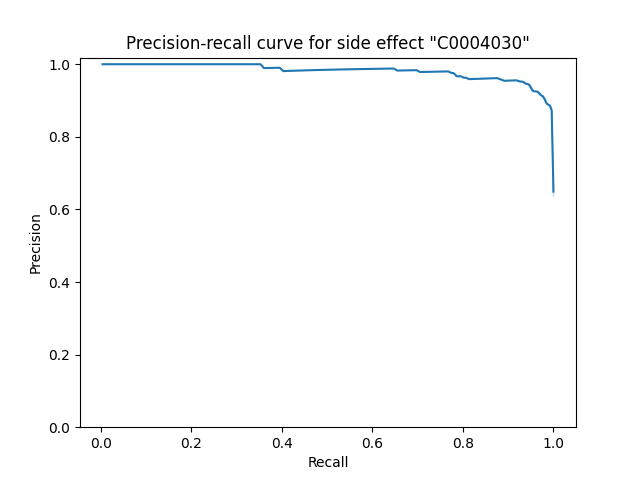

Supplement: btae706_Supplementary_Data [file btae706_supplementary_data.zip › simple_selfloops/figures/C0004030/precision_recall.png]

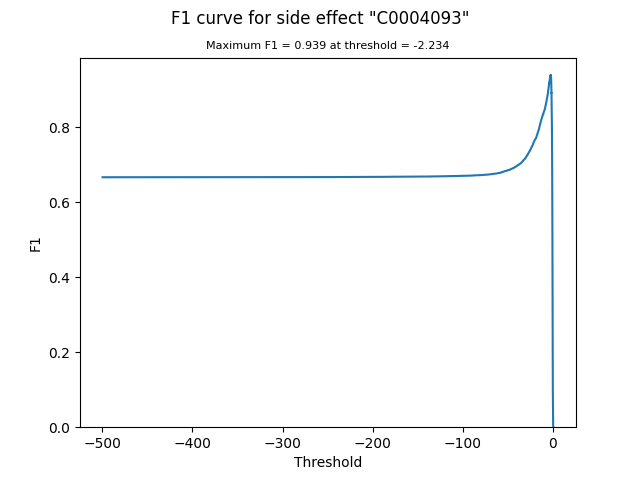

Supplement: btae706_Supplementary_Data [file btae706_supplementary_data.zip › simple_selfloops/figures/C0004093/F1_curve.png]

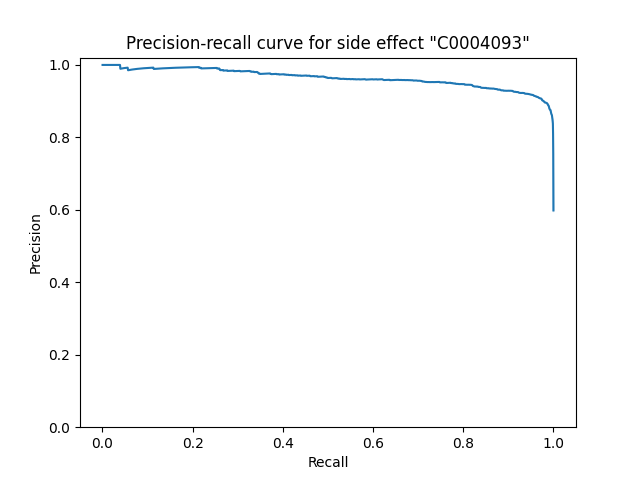

Supplement: btae706_Supplementary_Data [file btae706_supplementary_data.zip › simple_selfloops/figures/C0004093/precision_recall.png]

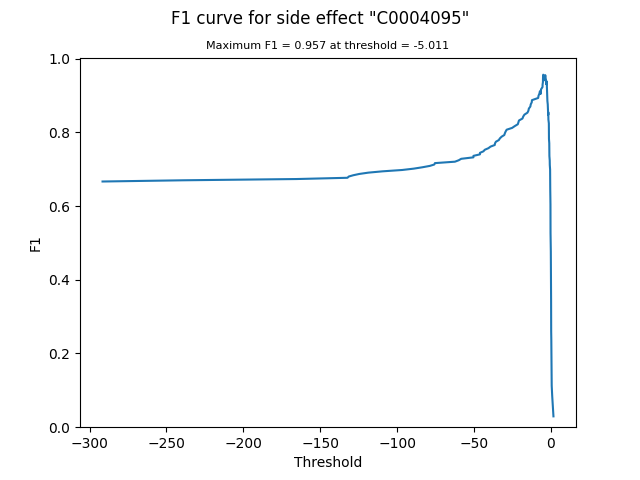

Supplement: btae706_Supplementary_Data [file btae706_supplementary_data.zip › simple_selfloops/figures/C0004095/F1_curve.png]

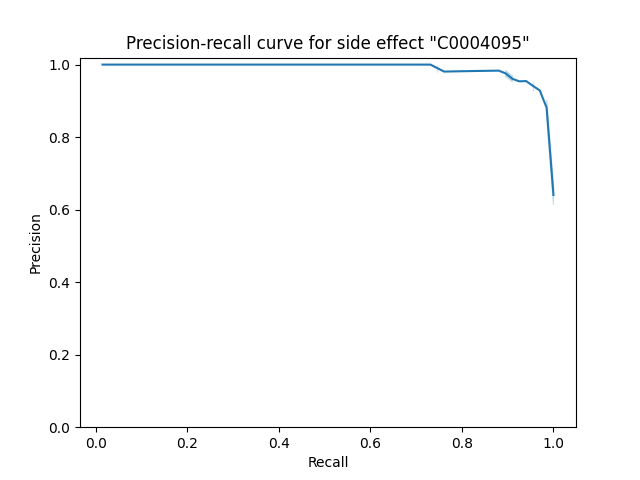

Supplement: btae706_Supplementary_Data [file btae706_supplementary_data.zip › simple_selfloops/figures/C0004095/precision_recall.png]

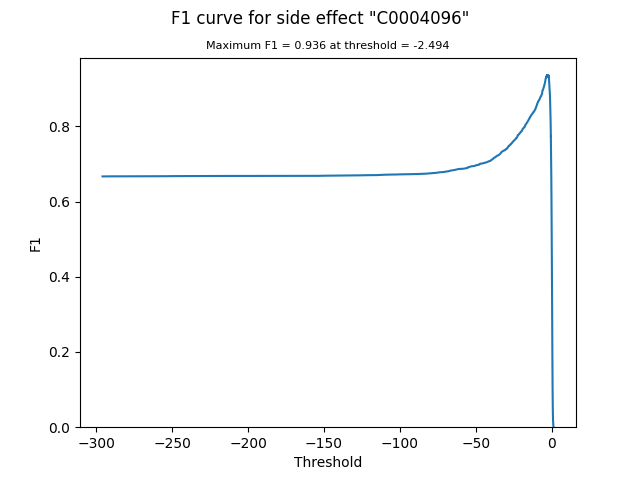

Supplement: btae706_Supplementary_Data [file btae706_supplementary_data.zip › simple_selfloops/figures/C0004096/F1_curve.png]

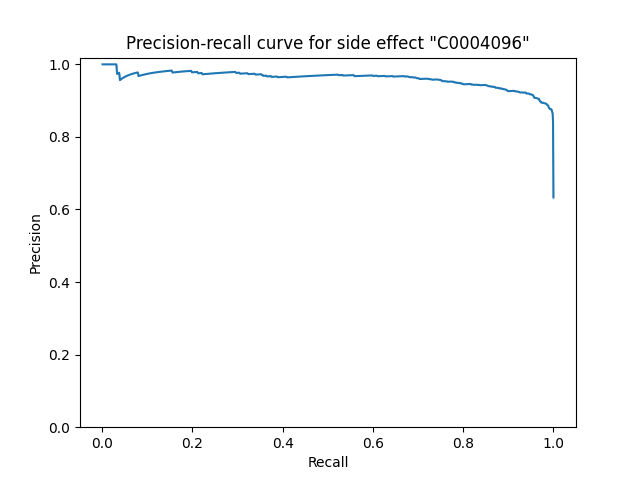

Supplement: btae706_Supplementary_Data [file btae706_supplementary_data.zip › simple_selfloops/figures/C0004096/precision_recall.png]

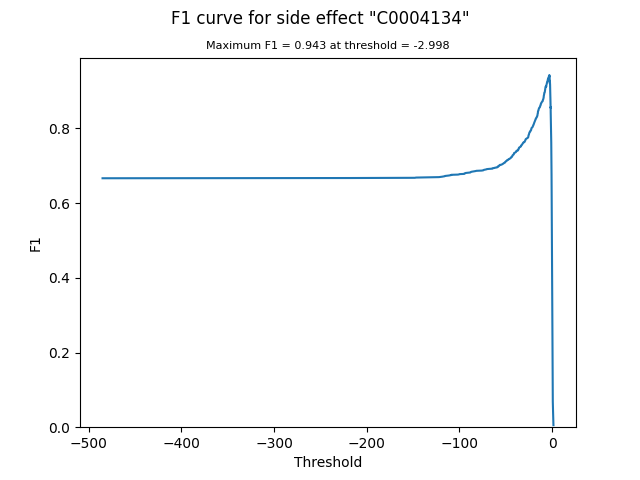

Supplement: btae706_Supplementary_Data [file btae706_supplementary_data.zip › simple_selfloops/figures/C0004134/F1_curve.png]

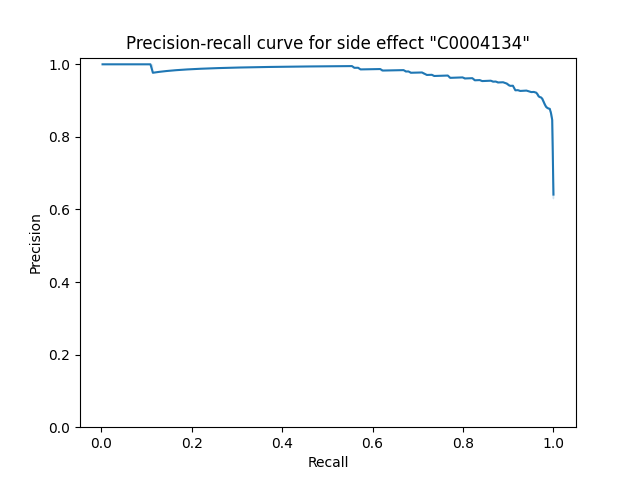

Supplement: btae706_Supplementary_Data [file btae706_supplementary_data.zip › simple_selfloops/figures/C0004134/precision_recall.png]

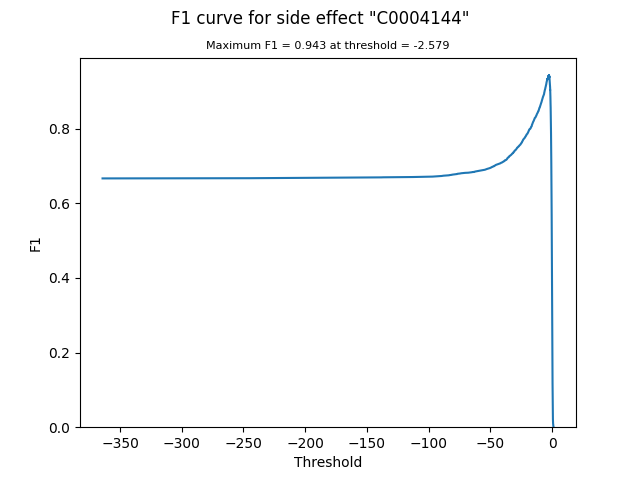

Supplement: btae706_Supplementary_Data [file btae706_supplementary_data.zip › simple_selfloops/figures/C0004144/F1_curve.png]

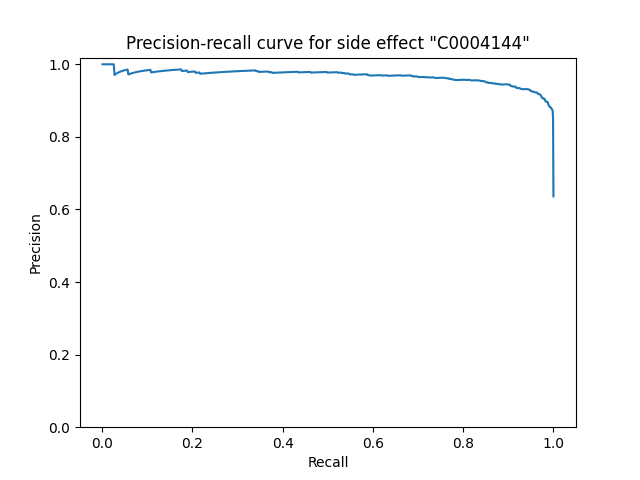

Supplement: btae706_Supplementary_Data [file btae706_supplementary_data.zip › simple_selfloops/figures/C0004144/precision_recall.png]

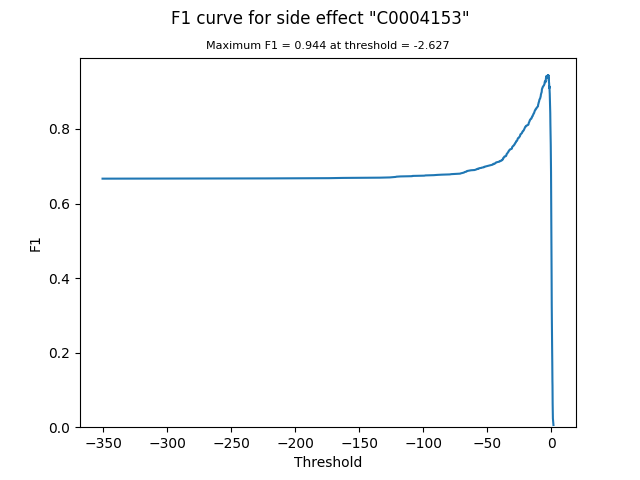

Supplement: btae706_Supplementary_Data [file btae706_supplementary_data.zip › simple_selfloops/figures/C0004153/F1_curve.png]

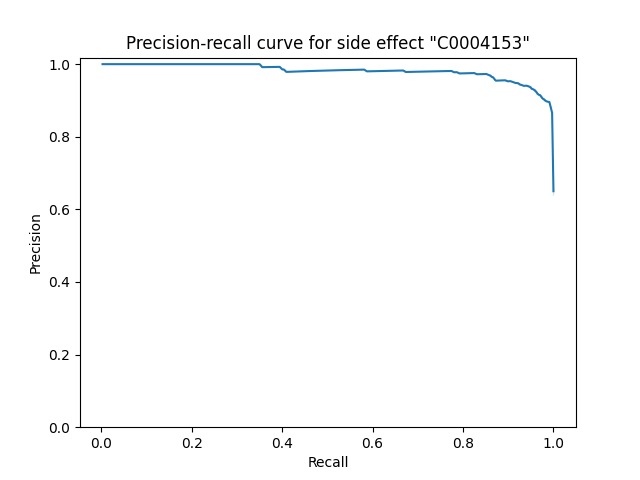

Supplement: btae706_Supplementary_Data [file btae706_supplementary_data.zip › simple_selfloops/figures/C0004153/precision_recall.png]

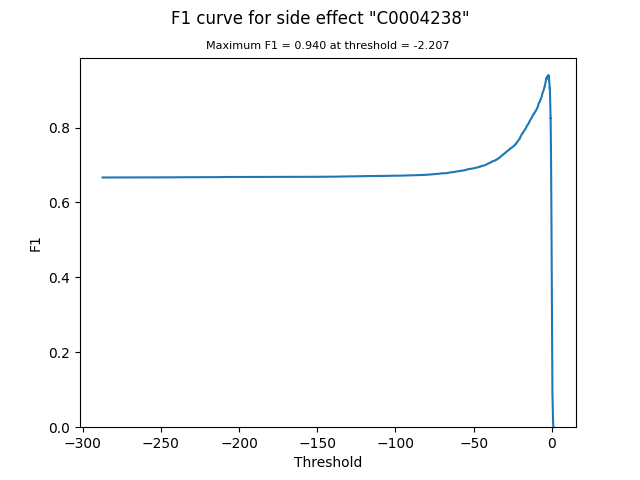

Supplement: btae706_Supplementary_Data [file btae706_supplementary_data.zip › simple_selfloops/figures/C0004238/F1_curve.png]

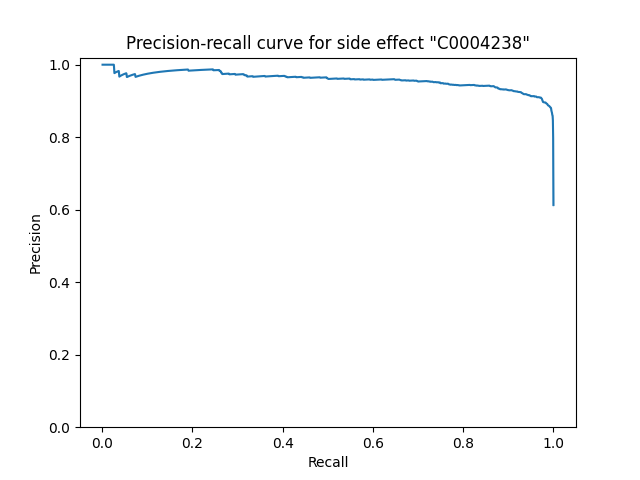

Supplement: btae706_Supplementary_Data [file btae706_supplementary_data.zip › simple_selfloops/figures/C0004238/precision_recall.png]

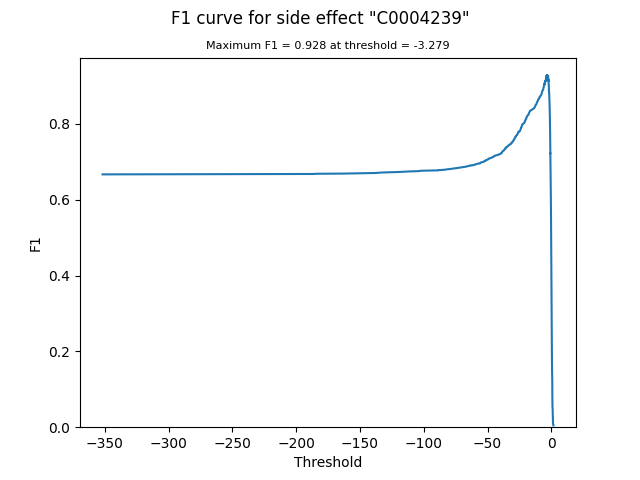

Supplement: btae706_Supplementary_Data [file btae706_supplementary_data.zip › simple_selfloops/figures/C0004239/F1_curve.png]

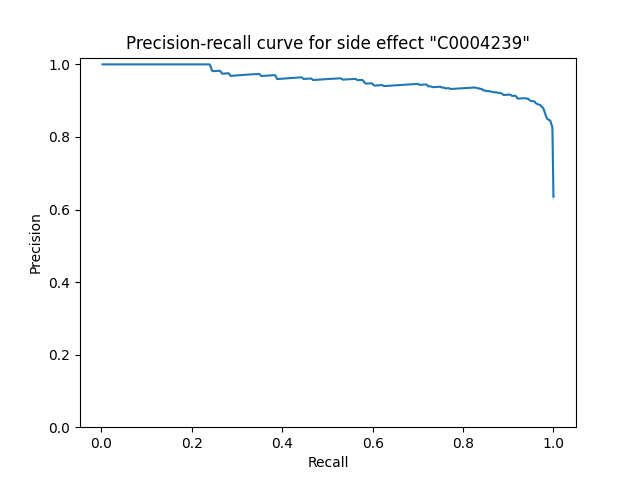

Supplement: btae706_Supplementary_Data [file btae706_supplementary_data.zip › simple_selfloops/figures/C0004239/precision_recall.png]

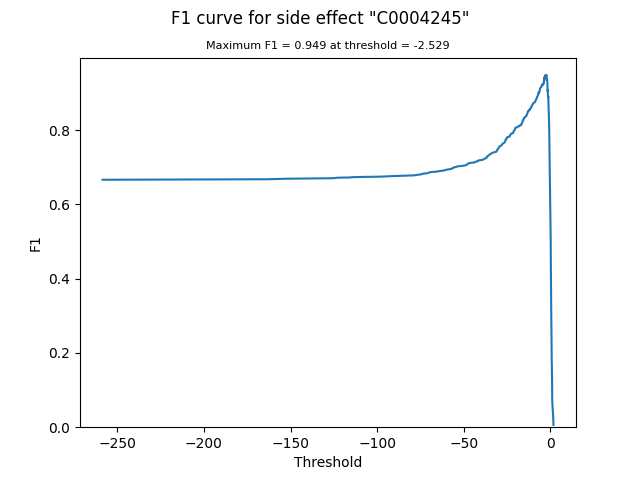

Supplement: btae706_Supplementary_Data [file btae706_supplementary_data.zip › simple_selfloops/figures/C0004245/F1_curve.png]

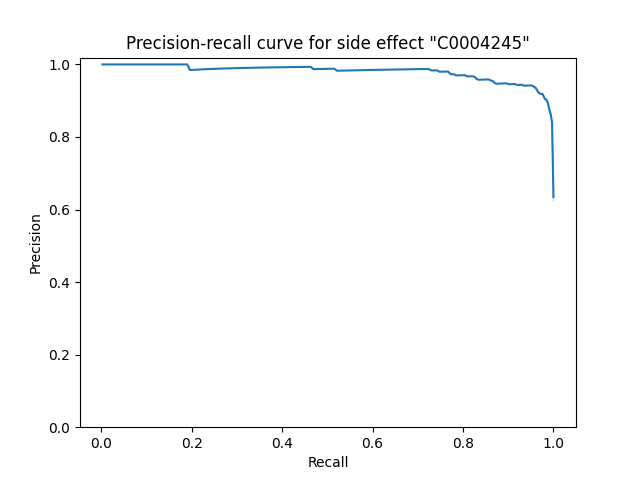

Supplement: btae706_Supplementary_Data [file btae706_supplementary_data.zip › simple_selfloops/figures/C0004245/precision_recall.png]

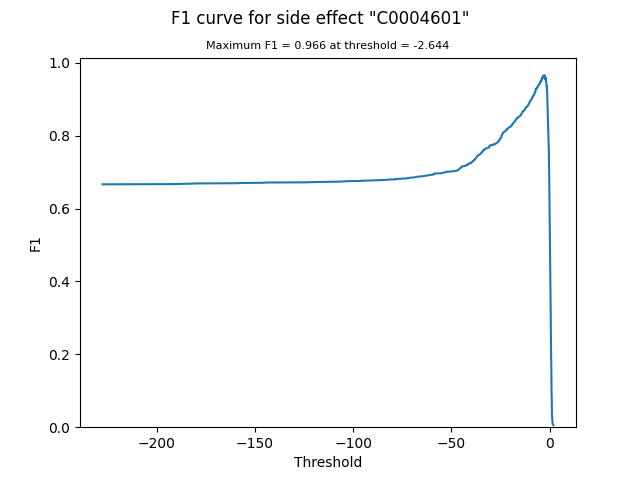

Supplement: btae706_Supplementary_Data [file btae706_supplementary_data.zip › simple_selfloops/figures/C0004601/F1_curve.png]

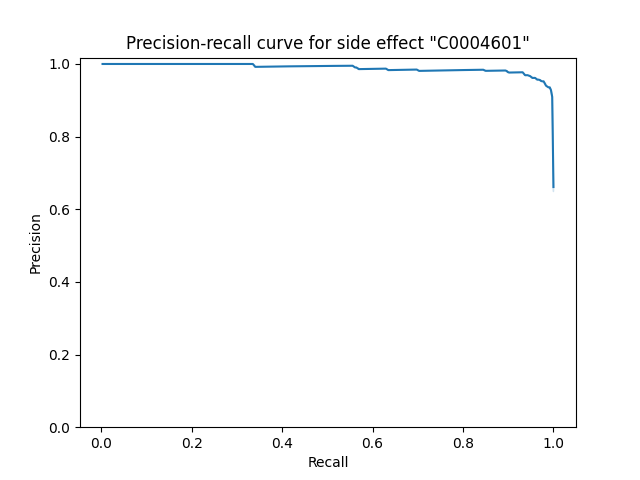

Supplement: btae706_Supplementary_Data [file btae706_supplementary_data.zip › simple_selfloops/figures/C0004601/precision_recall.png]

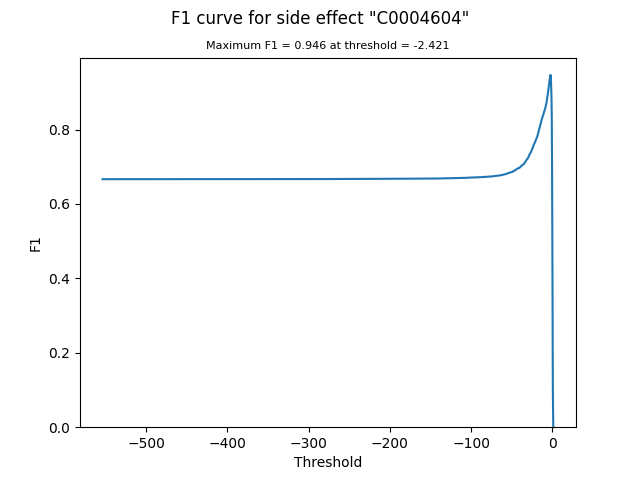

Supplement: btae706_Supplementary_Data [file btae706_supplementary_data.zip › simple_selfloops/figures/C0004604/F1_curve.png]

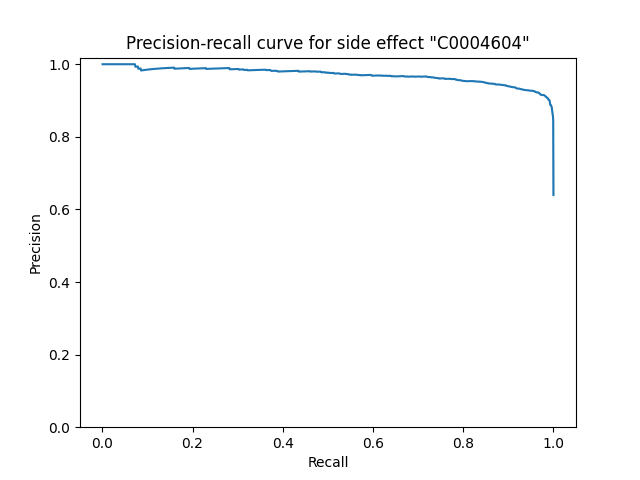

Supplement: btae706_Supplementary_Data [file btae706_supplementary_data.zip › simple_selfloops/figures/C0004604/precision_recall.png]

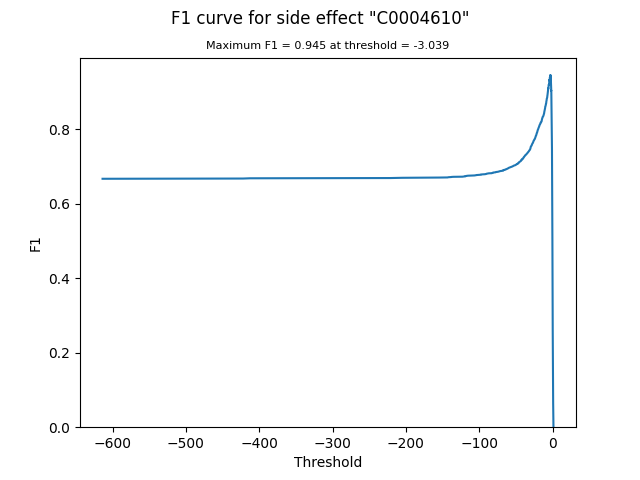

Supplement: btae706_Supplementary_Data [file btae706_supplementary_data.zip › simple_selfloops/figures/C0004610/F1_curve.png]

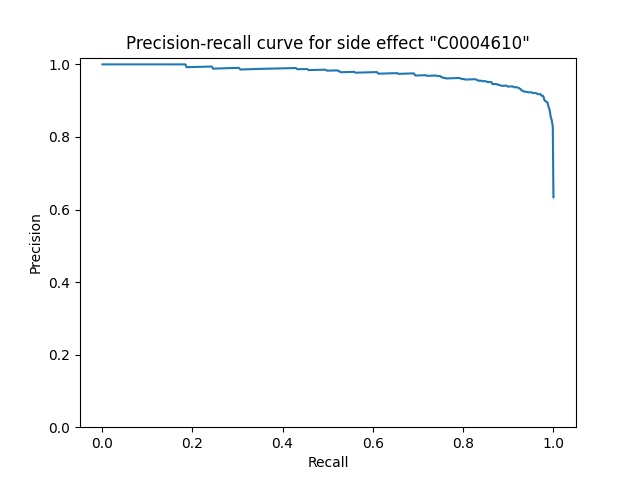

Supplement: btae706_Supplementary_Data [file btae706_supplementary_data.zip › simple_selfloops/figures/C0004610/precision_recall.png]

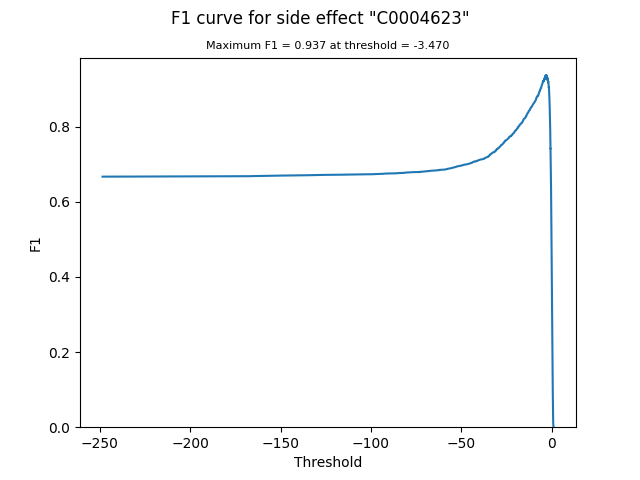

Supplement: btae706_Supplementary_Data [file btae706_supplementary_data.zip › simple_selfloops/figures/C0004623/F1_curve.png]

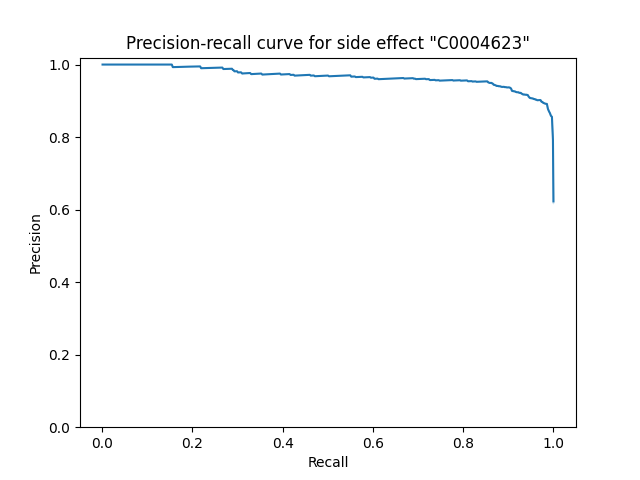

Supplement: btae706_Supplementary_Data [file btae706_supplementary_data.zip › simple_selfloops/figures/C0004623/precision_recall.png]

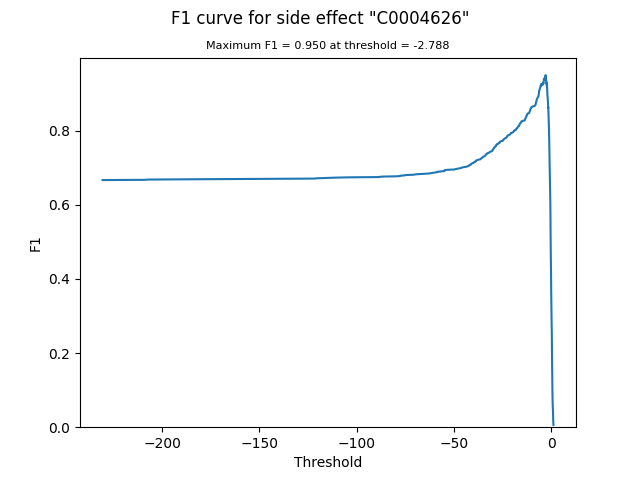

Supplement: btae706_Supplementary_Data [file btae706_supplementary_data.zip › simple_selfloops/figures/C0004626/F1_curve.png]

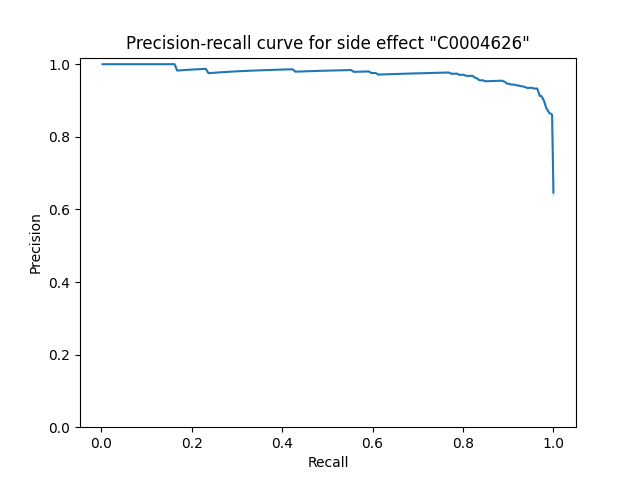

Supplement: btae706_Supplementary_Data [file btae706_supplementary_data.zip › simple_selfloops/figures/C0004626/precision_recall.png]

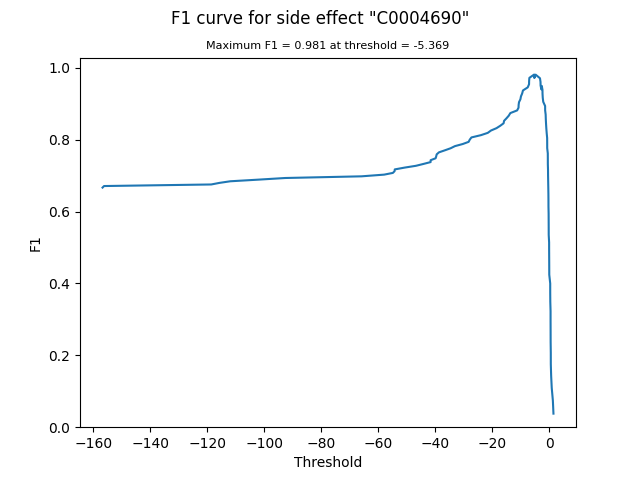

Supplement: btae706_Supplementary_Data [file btae706_supplementary_data.zip › simple_selfloops/figures/C0004690/F1_curve.png]

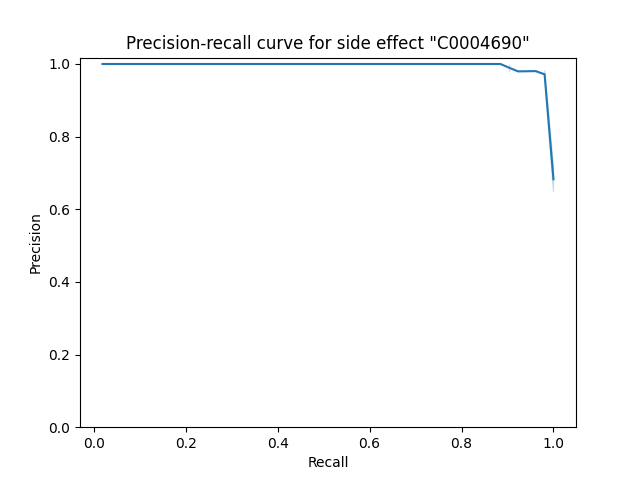

Supplement: btae706_Supplementary_Data [file btae706_supplementary_data.zip › simple_selfloops/figures/C0004690/precision_recall.png]

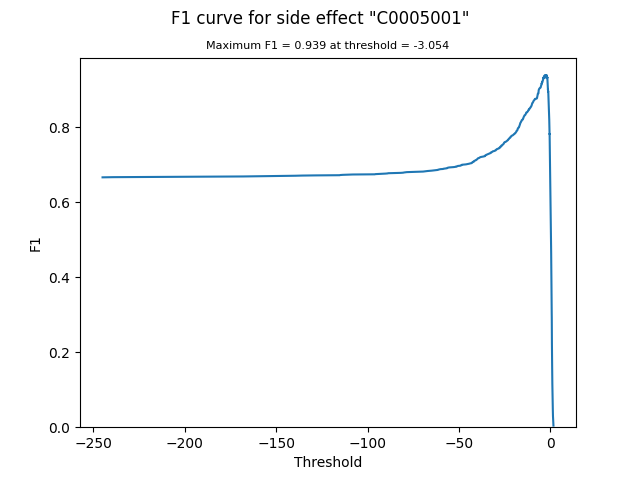

Supplement: btae706_Supplementary_Data [file btae706_supplementary_data.zip › simple_selfloops/figures/C0005001/F1_curve.png]

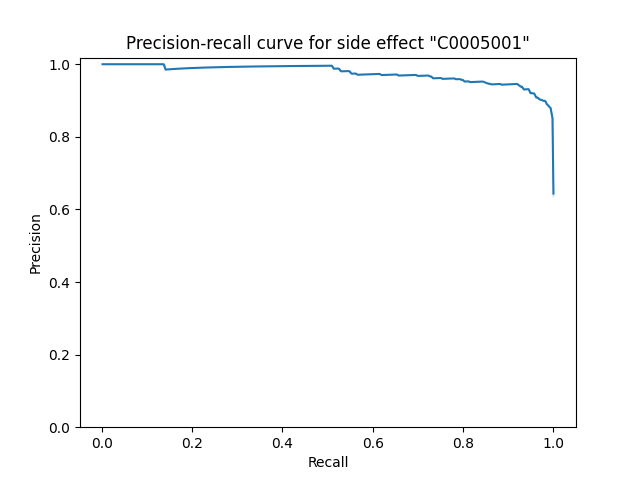

Supplement: btae706_Supplementary_Data [file btae706_supplementary_data.zip › simple_selfloops/figures/C0005001/precision_recall.png]

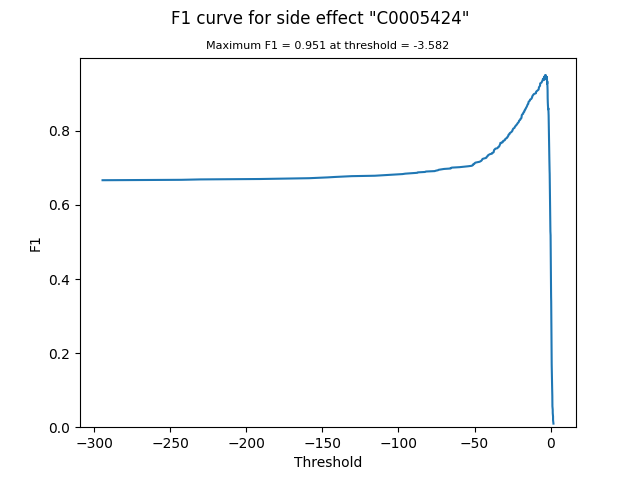

Supplement: btae706_Supplementary_Data [file btae706_supplementary_data.zip › simple_selfloops/figures/C0005424/F1_curve.png]

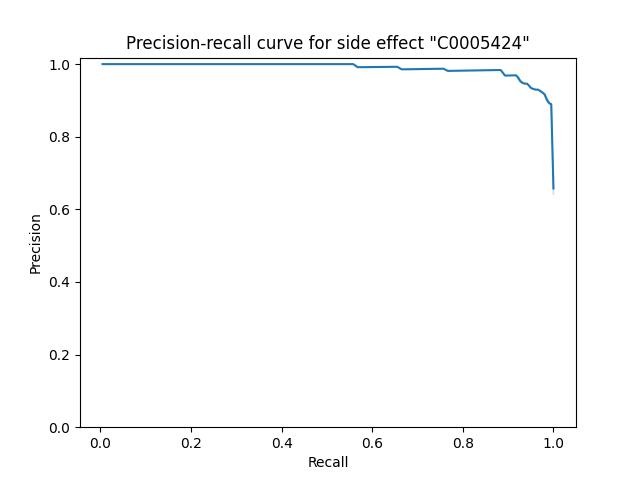

Supplement: btae706_Supplementary_Data [file btae706_supplementary_data.zip › simple_selfloops/figures/C0005424/precision_recall.png]

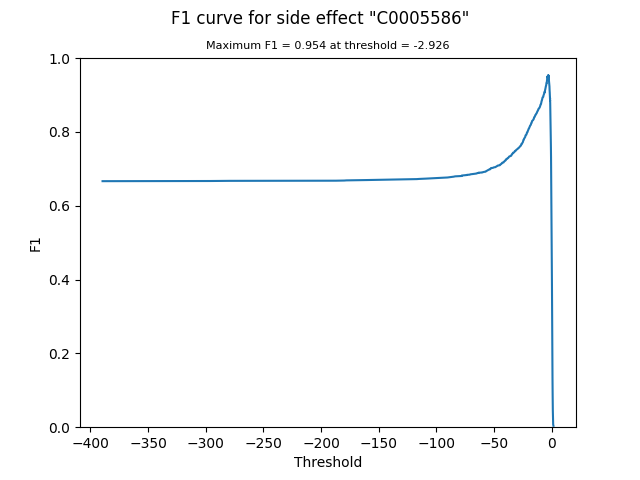

Supplement: btae706_Supplementary_Data [file btae706_supplementary_data.zip › simple_selfloops/figures/C0005586/F1_curve.png]

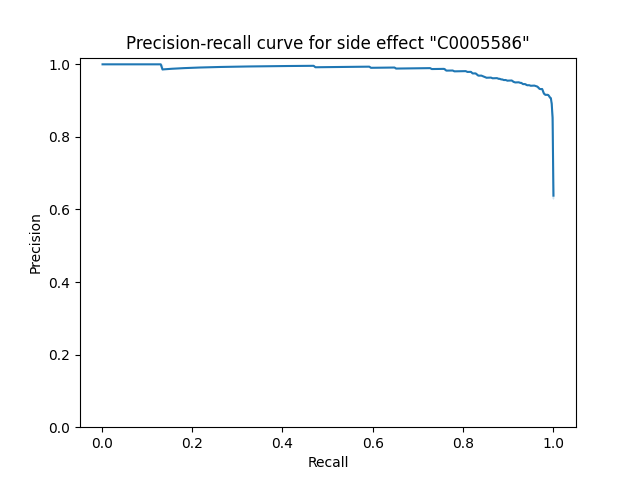

Supplement: btae706_Supplementary_Data [file btae706_supplementary_data.zip › simple_selfloops/figures/C0005586/precision_recall.png]

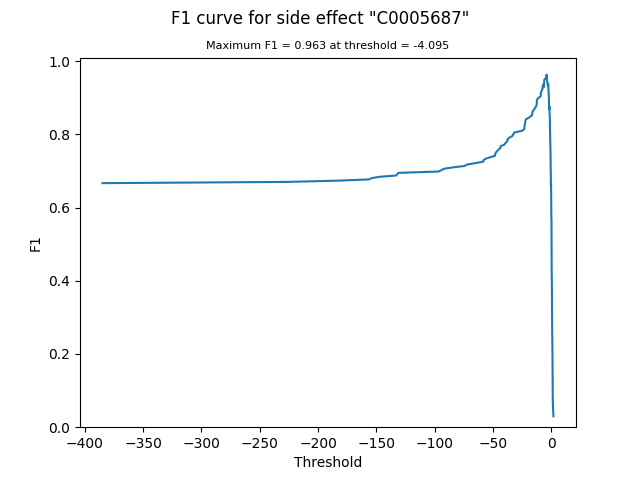

Supplement: btae706_Supplementary_Data [file btae706_supplementary_data.zip › simple_selfloops/figures/C0005687/F1_curve.png]

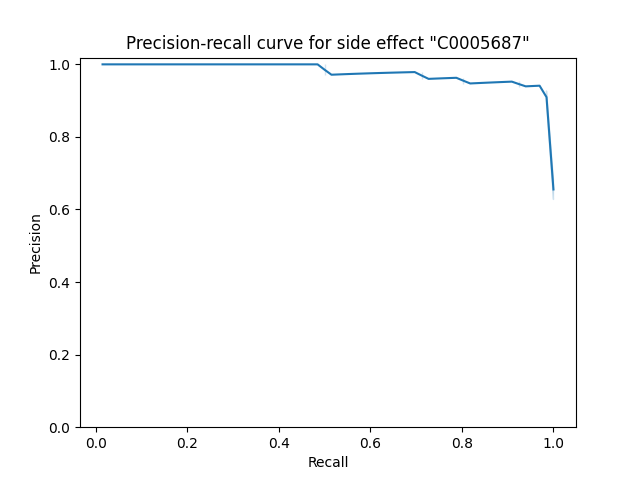

Supplement: btae706_Supplementary_Data [file btae706_supplementary_data.zip › simple_selfloops/figures/C0005687/precision_recall.png]

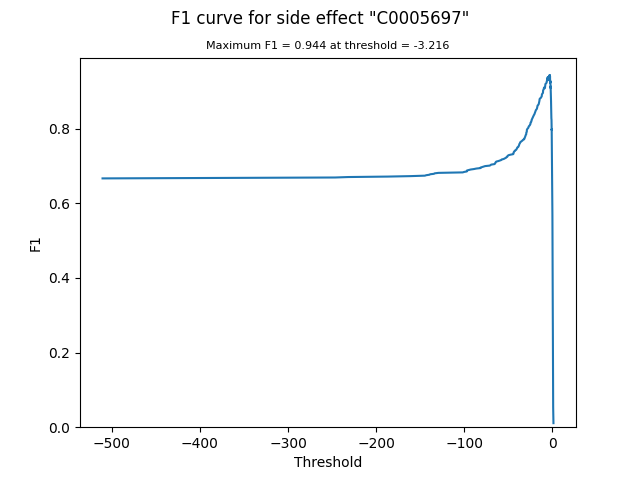

Supplement: btae706_Supplementary_Data [file btae706_supplementary_data.zip › simple_selfloops/figures/C0005697/F1_curve.png]

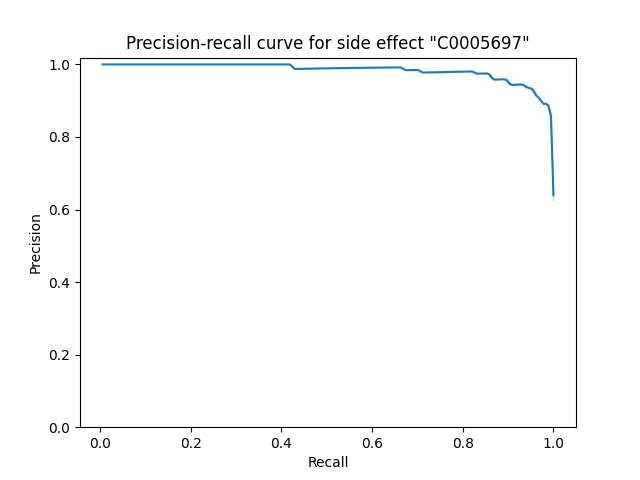

Supplement: btae706_Supplementary_Data [file btae706_supplementary_data.zip › simple_selfloops/figures/C0005697/precision_recall.png]

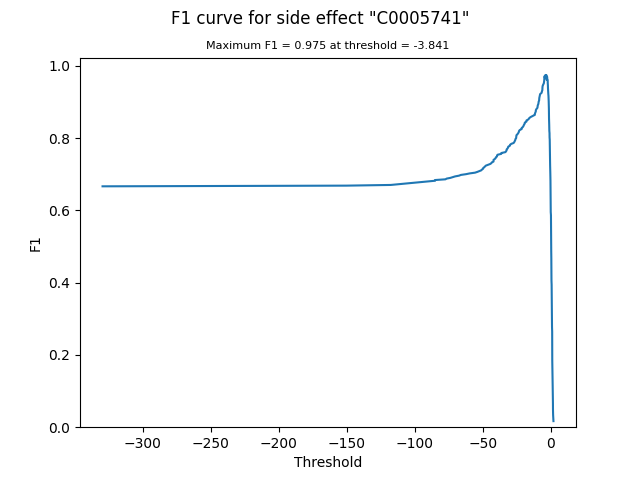

Supplement: btae706_Supplementary_Data [file btae706_supplementary_data.zip › simple_selfloops/figures/C0005741/F1_curve.png]

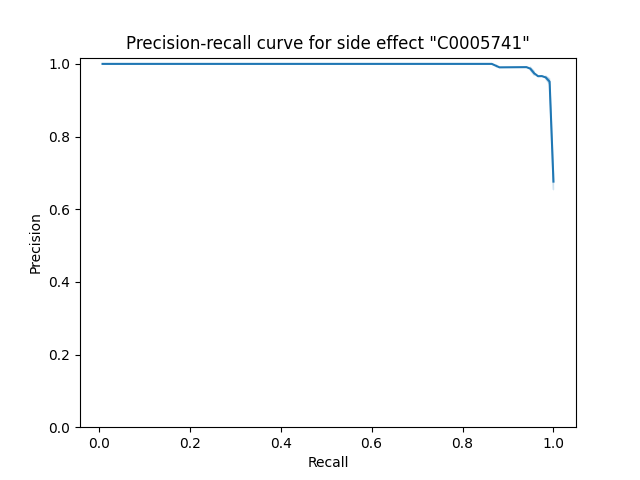

Supplement: btae706_Supplementary_Data [file btae706_supplementary_data.zip › simple_selfloops/figures/C0005741/precision_recall.png]

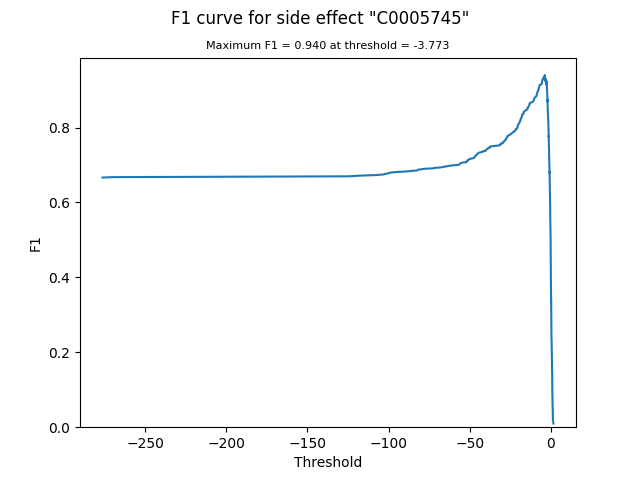

Supplement: btae706_Supplementary_Data [file btae706_supplementary_data.zip › simple_selfloops/figures/C0005745/F1_curve.png]

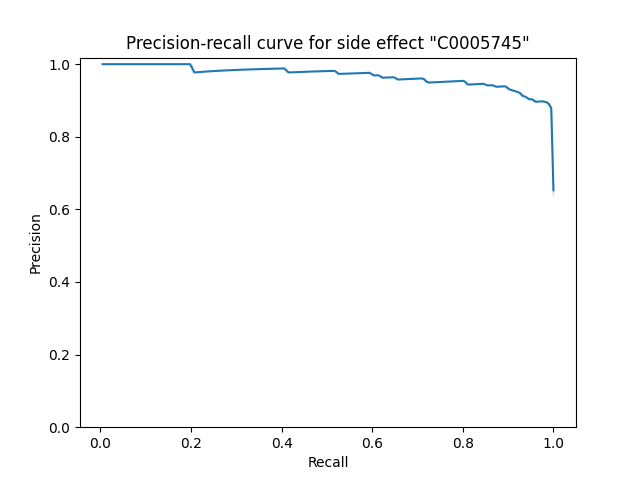

Supplement: btae706_Supplementary_Data [file btae706_supplementary_data.zip › simple_selfloops/figures/C0005745/precision_recall.png]

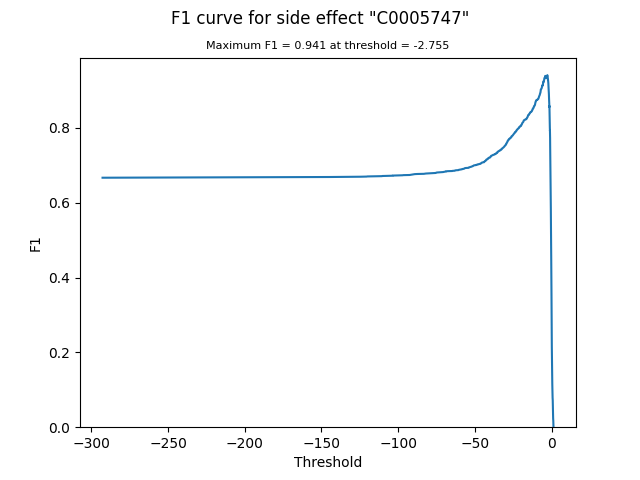

Supplement: btae706_Supplementary_Data [file btae706_supplementary_data.zip › simple_selfloops/figures/C0005747/F1_curve.png]

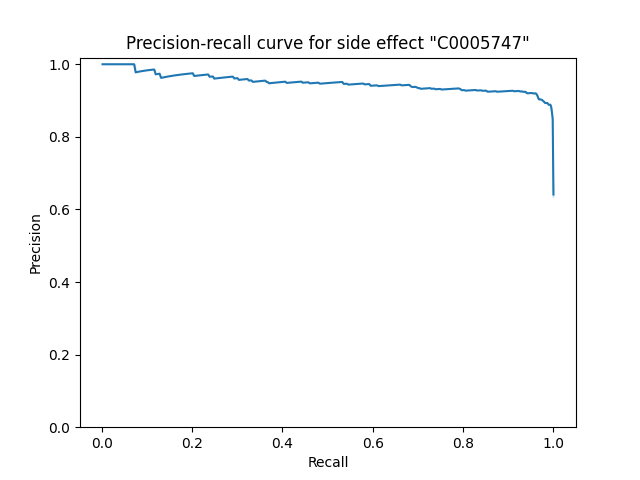

Supplement: btae706_Supplementary_Data [file btae706_supplementary_data.zip › simple_selfloops/figures/C0005747/precision_recall.png]

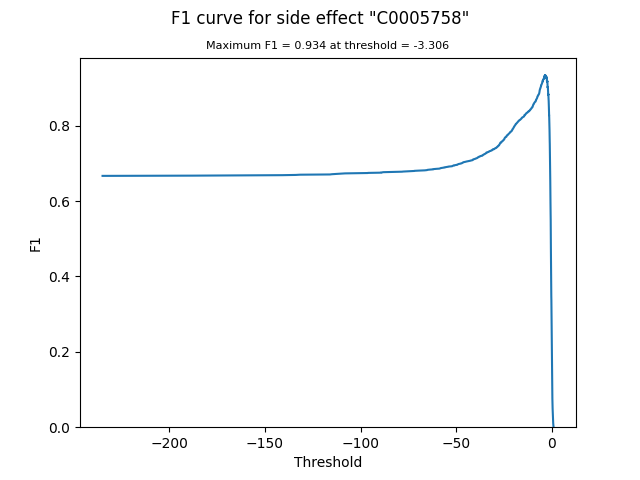

Supplement: btae706_Supplementary_Data [file btae706_supplementary_data.zip › simple_selfloops/figures/C0005758/F1_curve.png]

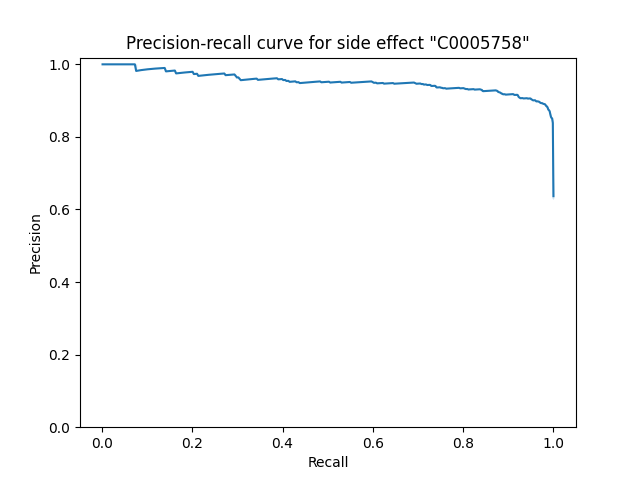

Supplement: btae706_Supplementary_Data [file btae706_supplementary_data.zip › simple_selfloops/figures/C0005758/precision_recall.png]

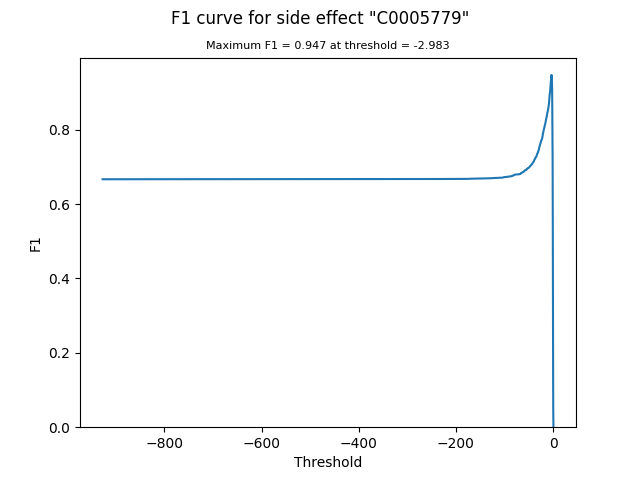

Supplement: btae706_Supplementary_Data [file btae706_supplementary_data.zip › simple_selfloops/figures/C0005779/F1_curve.png]

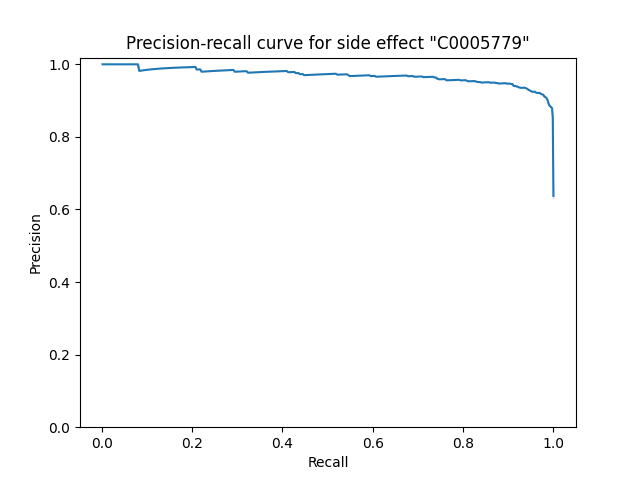

Supplement: btae706_Supplementary_Data [file btae706_supplementary_data.zip › simple_selfloops/figures/C0005779/precision_recall.png]

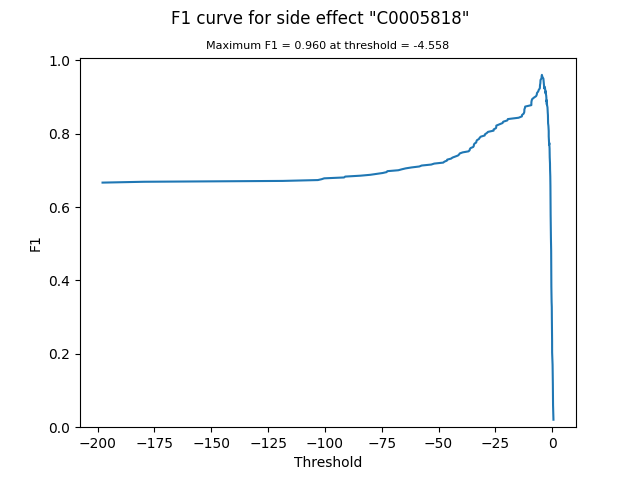

Supplement: btae706_Supplementary_Data [file btae706_supplementary_data.zip › simple_selfloops/figures/C0005818/F1_curve.png]

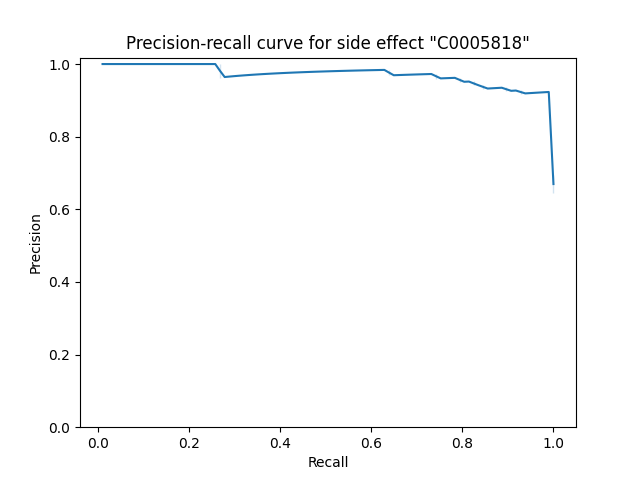

Supplement: btae706_Supplementary_Data [file btae706_supplementary_data.zip › simple_selfloops/figures/C0005818/precision_recall.png]

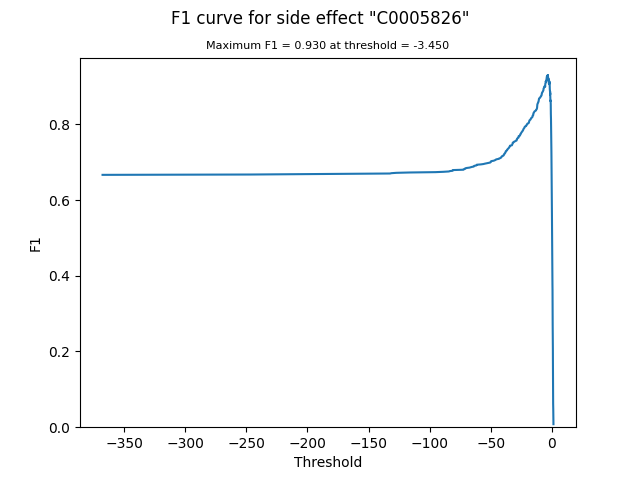

Supplement: btae706_Supplementary_Data [file btae706_supplementary_data.zip › simple_selfloops/figures/C0005826/F1_curve.png]

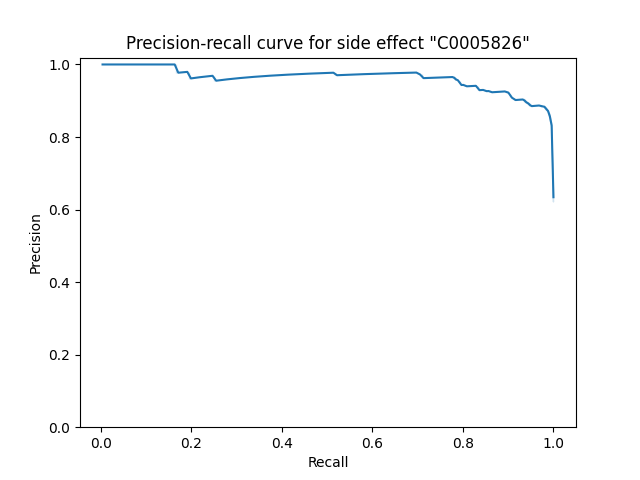

Supplement: btae706_Supplementary_Data [file btae706_supplementary_data.zip › simple_selfloops/figures/C0005826/precision_recall.png]

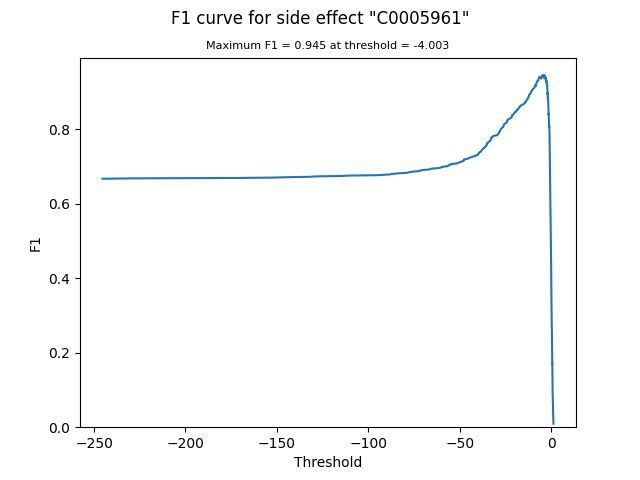

Supplement: btae706_Supplementary_Data [file btae706_supplementary_data.zip › simple_selfloops/figures/C0005961/F1_curve.png]

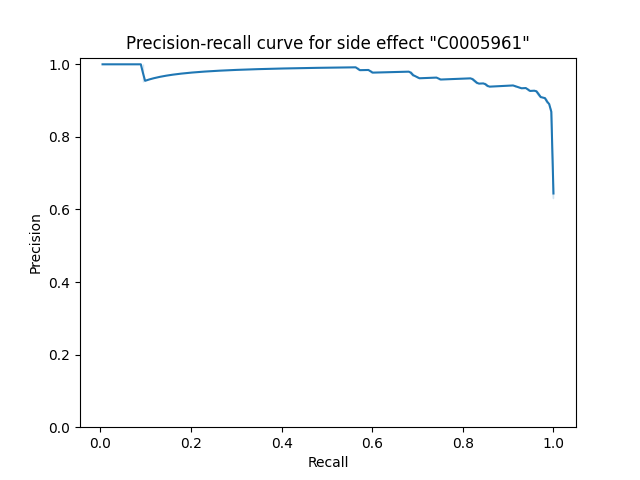

Supplement: btae706_Supplementary_Data [file btae706_supplementary_data.zip › simple_selfloops/figures/C0005961/precision_recall.png]

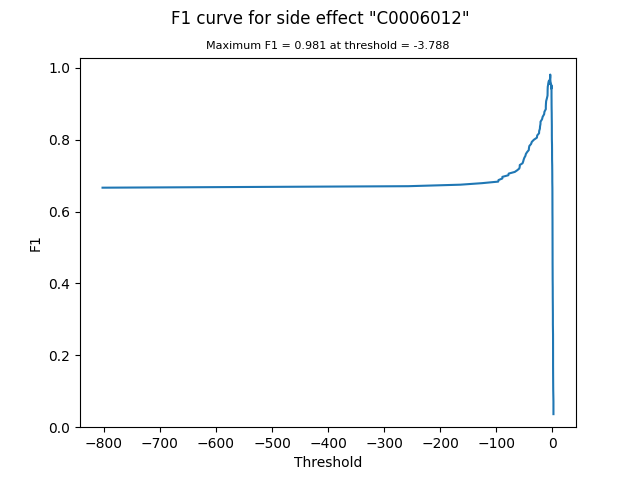

Supplement: btae706_Supplementary_Data [file btae706_supplementary_data.zip › simple_selfloops/figures/C0006012/F1_curve.png]

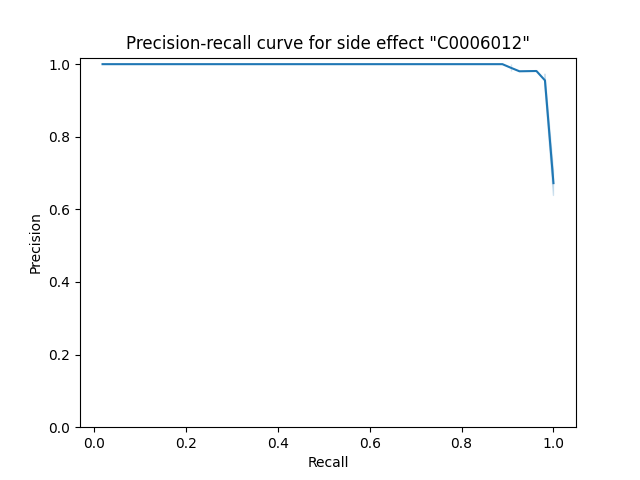

Supplement: btae706_Supplementary_Data [file btae706_supplementary_data.zip › simple_selfloops/figures/C0006012/precision_recall.png]

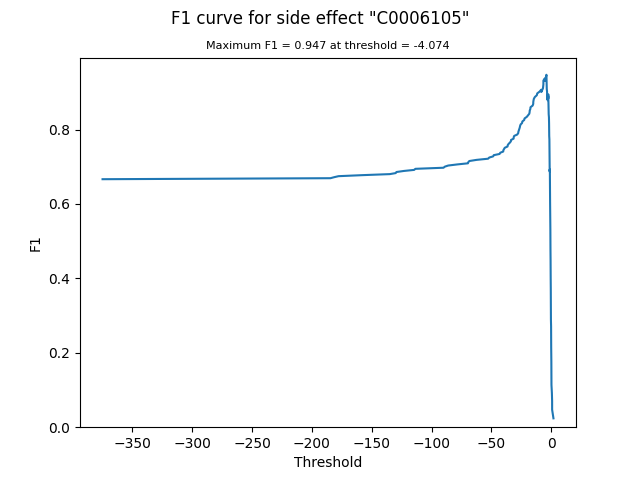

Supplement: btae706_Supplementary_Data [file btae706_supplementary_data.zip › simple_selfloops/figures/C0006105/F1_curve.png]

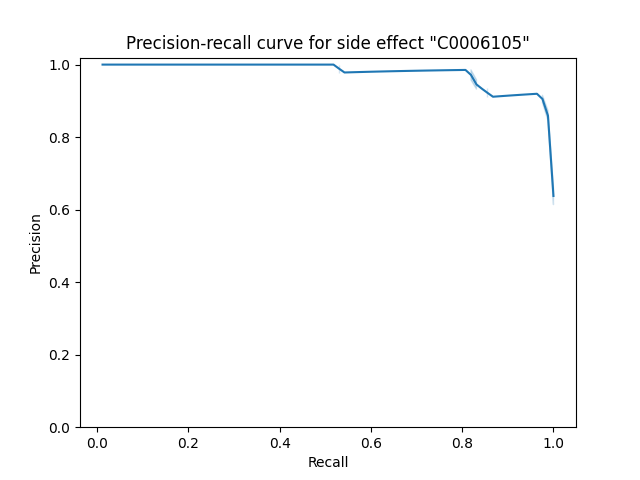

Supplement: btae706_Supplementary_Data [file btae706_supplementary_data.zip › simple_selfloops/figures/C0006105/precision_recall.png]

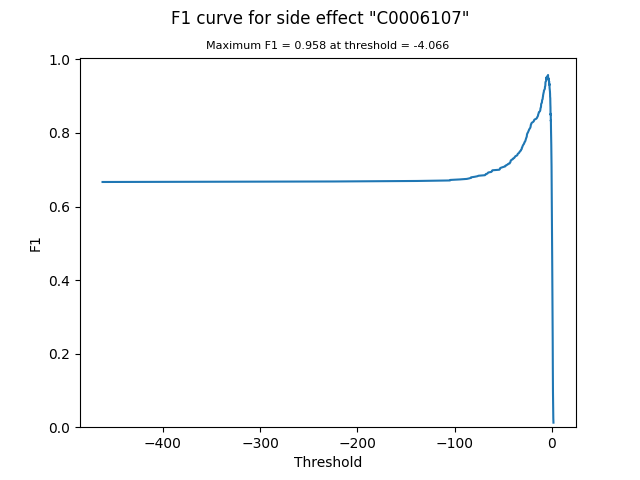

Supplement: btae706_Supplementary_Data [file btae706_supplementary_data.zip › simple_selfloops/figures/C0006107/F1_curve.png]

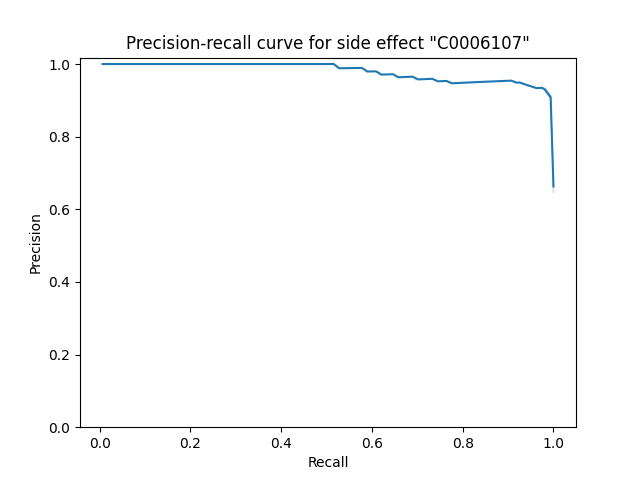

Supplement: btae706_Supplementary_Data [file btae706_supplementary_data.zip › simple_selfloops/figures/C0006107/precision_recall.png]

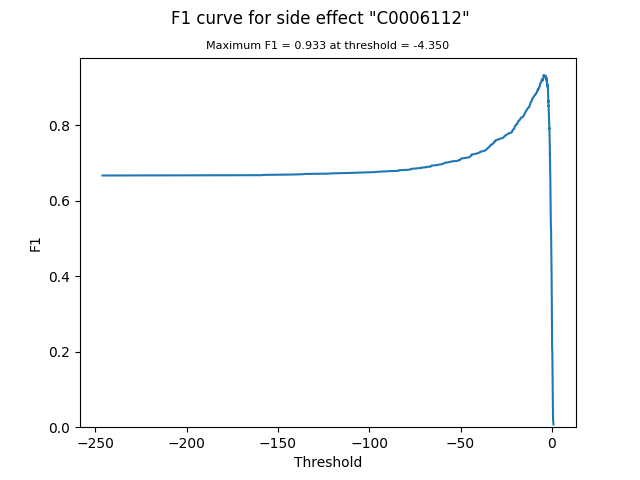

Supplement: btae706_Supplementary_Data [file btae706_supplementary_data.zip › simple_selfloops/figures/C0006112/F1_curve.png]

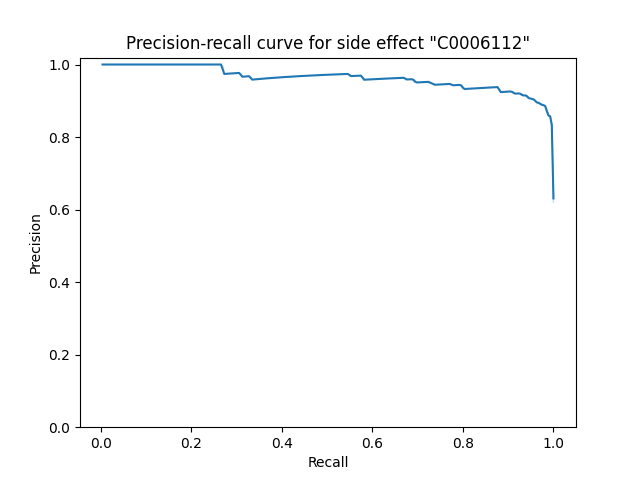

Supplement: btae706_Supplementary_Data [file btae706_supplementary_data.zip › simple_selfloops/figures/C0006112/precision_recall.png]

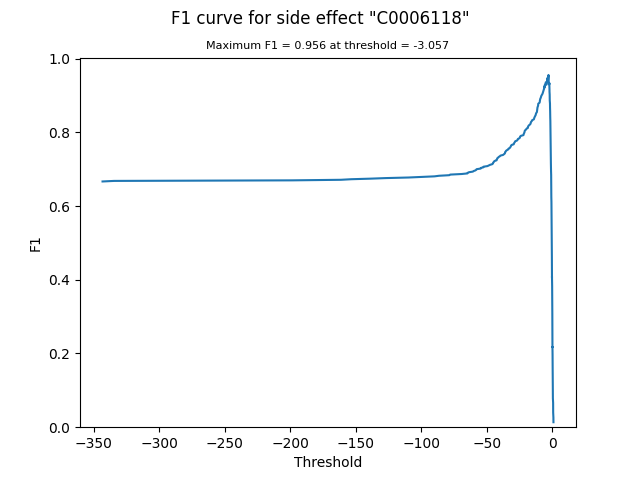

Supplement: btae706_Supplementary_Data [file btae706_supplementary_data.zip › simple_selfloops/figures/C0006118/F1_curve.png]

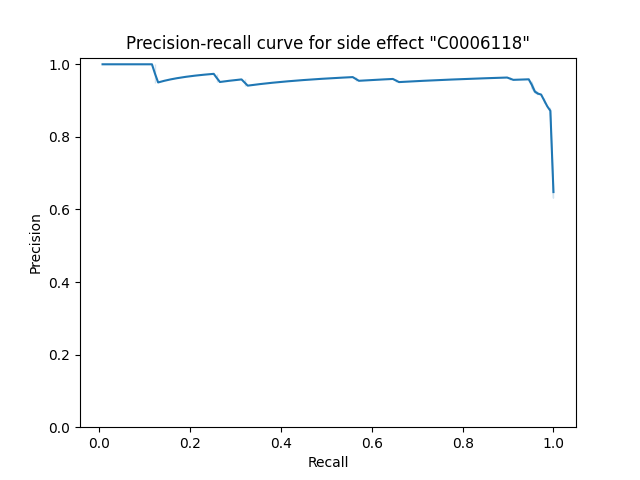

Supplement: btae706_Supplementary_Data [file btae706_supplementary_data.zip › simple_selfloops/figures/C0006118/precision_recall.png]

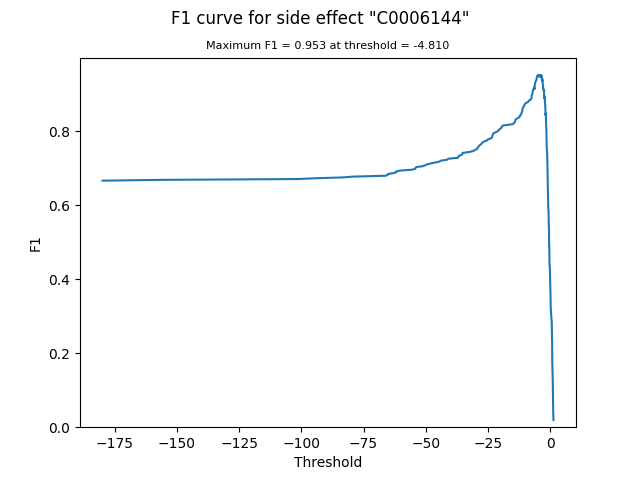

Supplement: btae706_Supplementary_Data [file btae706_supplementary_data.zip › simple_selfloops/figures/C0006144/F1_curve.png]

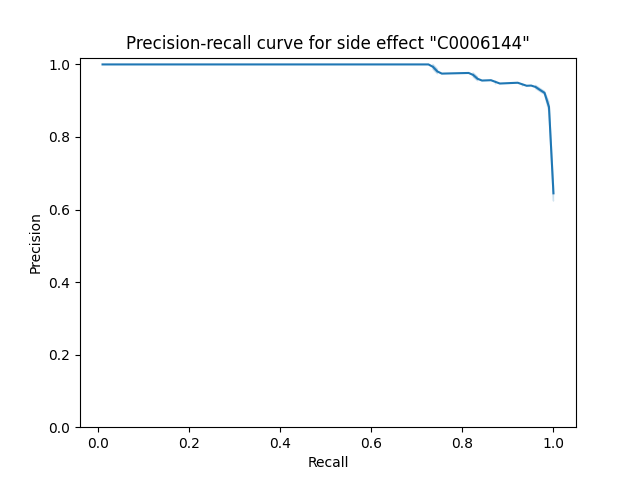

Supplement: btae706_Supplementary_Data [file btae706_supplementary_data.zip › simple_selfloops/figures/C0006144/precision_recall.png]

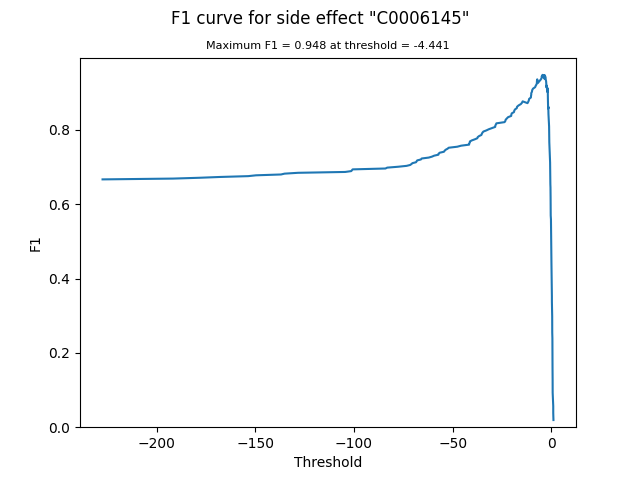

Supplement: btae706_Supplementary_Data [file btae706_supplementary_data.zip › simple_selfloops/figures/C0006145/F1_curve.png]

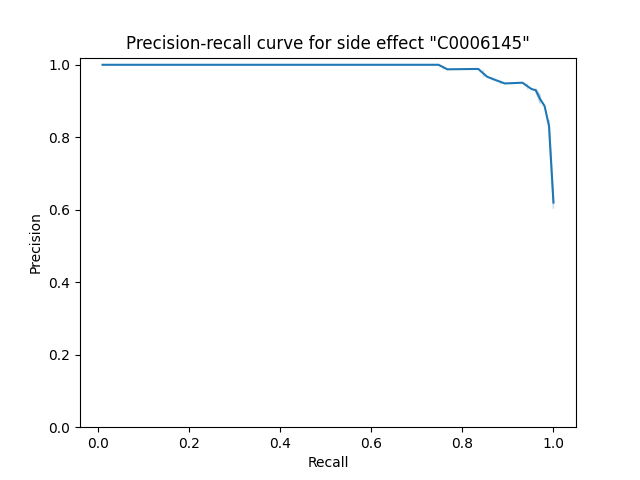

Supplement: btae706_Supplementary_Data [file btae706_supplementary_data.zip › simple_selfloops/figures/C0006145/precision_recall.png]

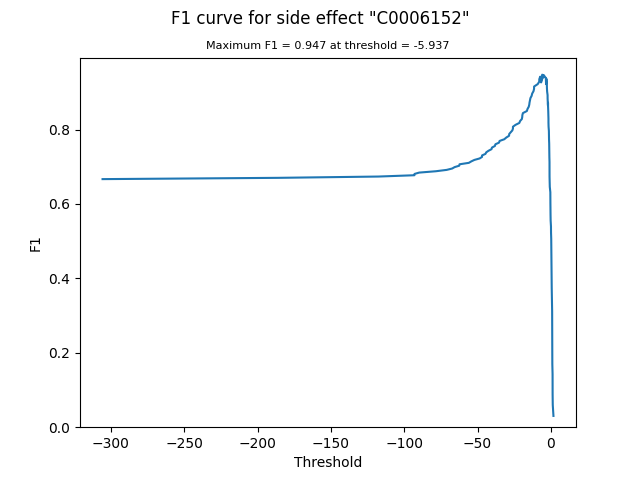

Supplement: btae706_Supplementary_Data [file btae706_supplementary_data.zip › simple_selfloops/figures/C0006152/F1_curve.png]

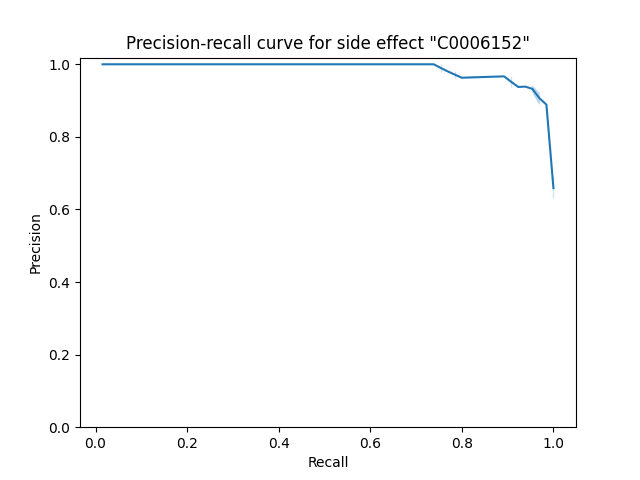

Supplement: btae706_Supplementary_Data [file btae706_supplementary_data.zip › simple_selfloops/figures/C0006152/precision_recall.png]

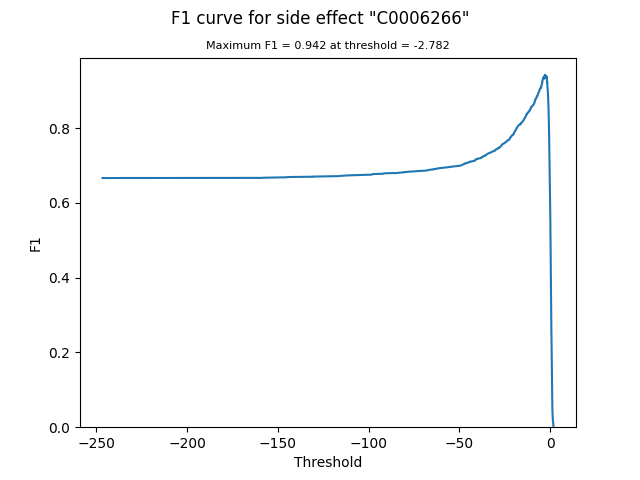

Supplement: btae706_Supplementary_Data [file btae706_supplementary_data.zip › simple_selfloops/figures/C0006266/F1_curve.png]

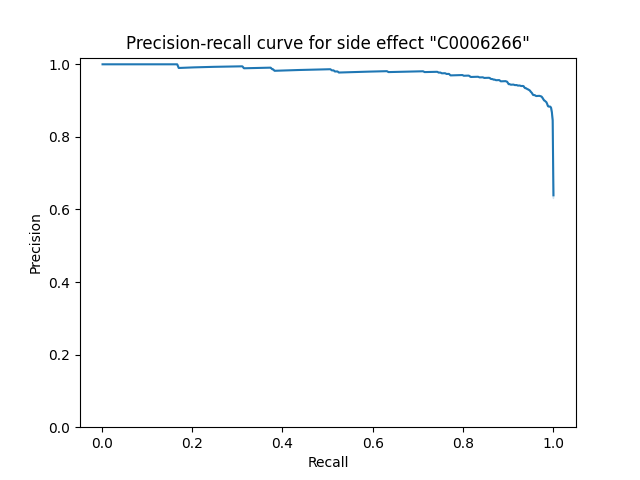

Supplement: btae706_Supplementary_Data [file btae706_supplementary_data.zip › simple_selfloops/figures/C0006266/precision_recall.png]

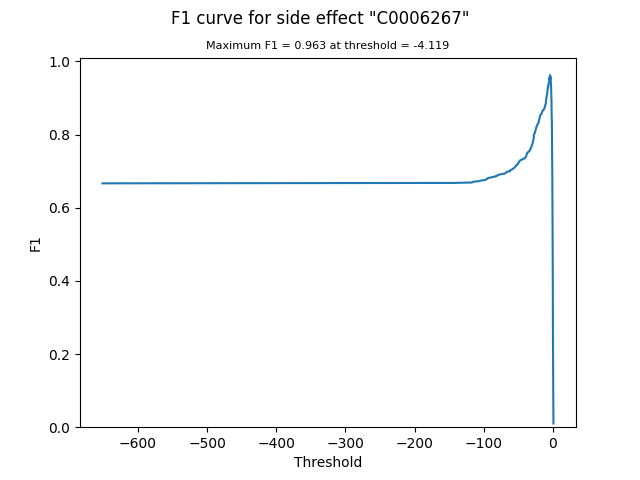

Supplement: btae706_Supplementary_Data [file btae706_supplementary_data.zip › simple_selfloops/figures/C0006267/F1_curve.png]

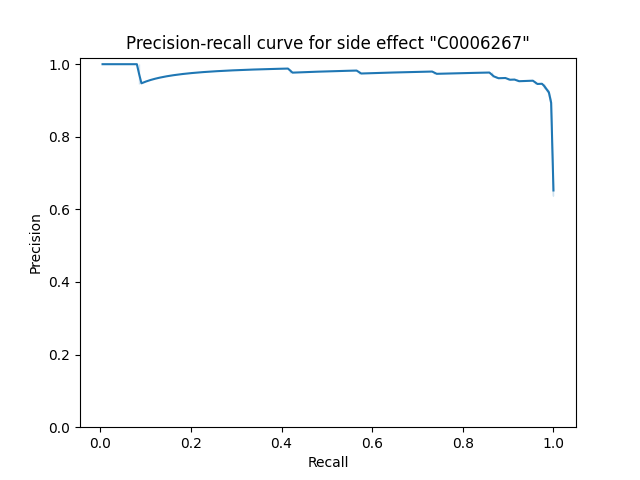

Supplement: btae706_Supplementary_Data [file btae706_supplementary_data.zip › simple_selfloops/figures/C0006267/precision_recall.png]

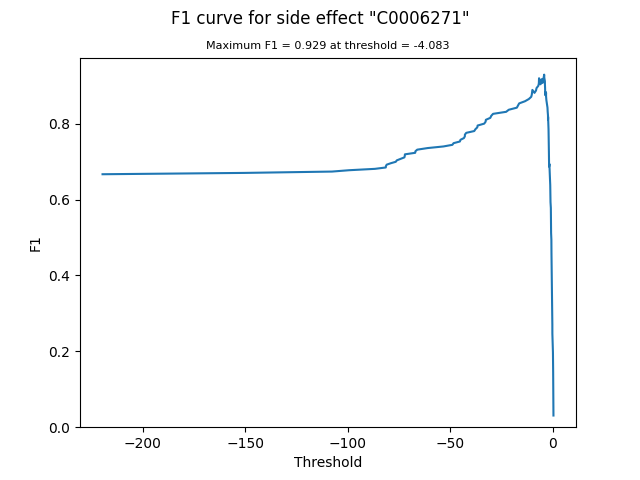

Supplement: btae706_Supplementary_Data [file btae706_supplementary_data.zip › simple_selfloops/figures/C0006271/F1_curve.png]

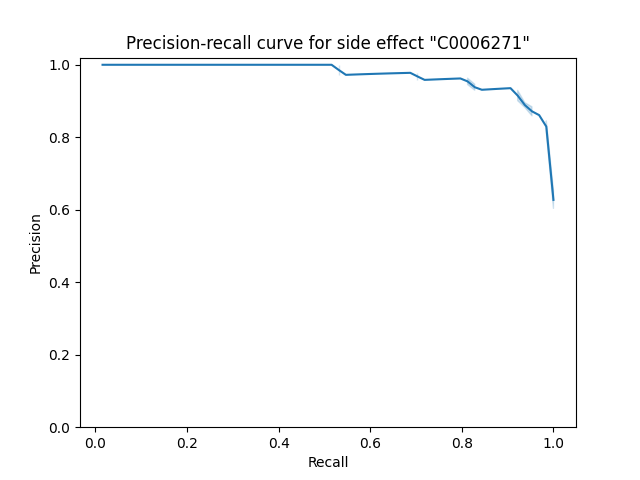

Supplement: btae706_Supplementary_Data [file btae706_supplementary_data.zip › simple_selfloops/figures/C0006271/precision_recall.png]

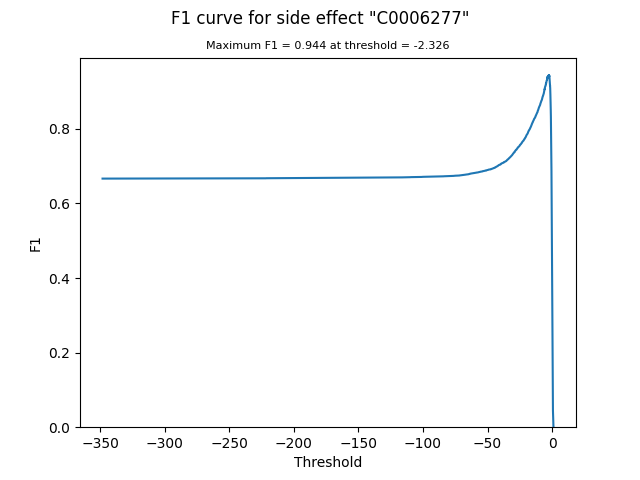

Supplement: btae706_Supplementary_Data [file btae706_supplementary_data.zip › simple_selfloops/figures/C0006277/F1_curve.png]

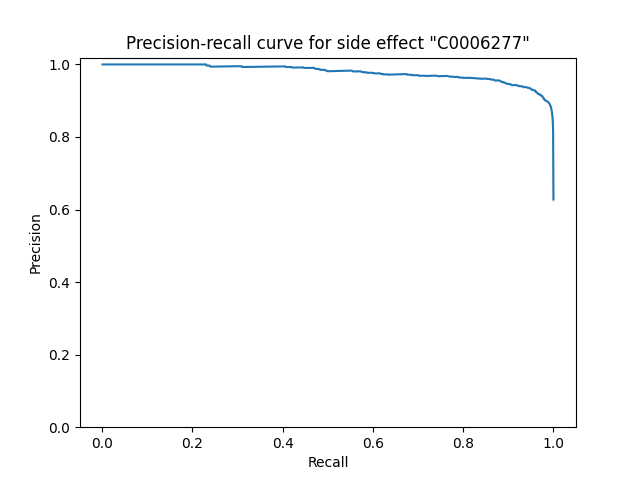

Supplement: btae706_Supplementary_Data [file btae706_supplementary_data.zip › simple_selfloops/figures/C0006277/precision_recall.png]

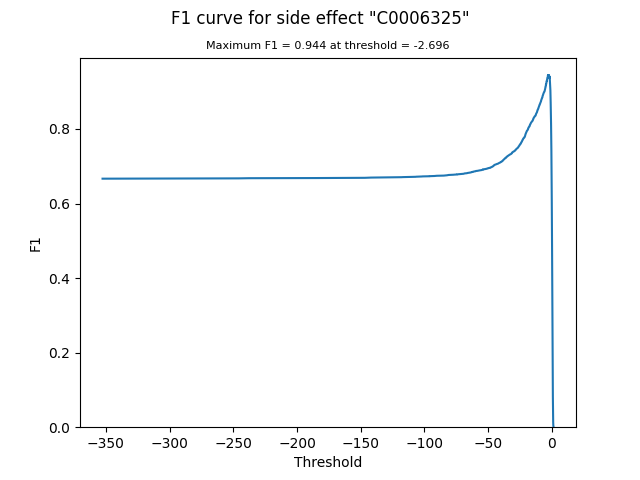

Supplement: btae706_Supplementary_Data [file btae706_supplementary_data.zip › simple_selfloops/figures/C0006325/F1_curve.png]

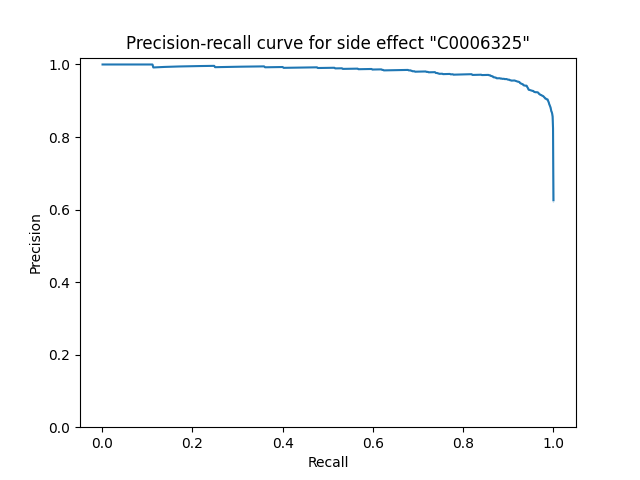

Supplement: btae706_Supplementary_Data [file btae706_supplementary_data.zip › simple_selfloops/figures/C0006325/precision_recall.png]

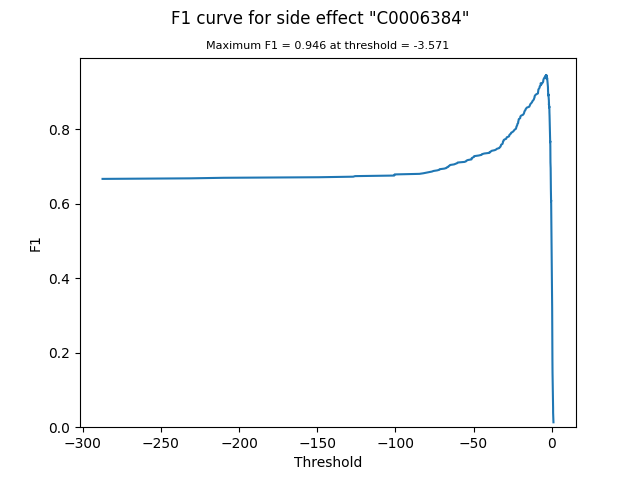

Supplement: btae706_Supplementary_Data [file btae706_supplementary_data.zip › simple_selfloops/figures/C0006384/F1_curve.png]

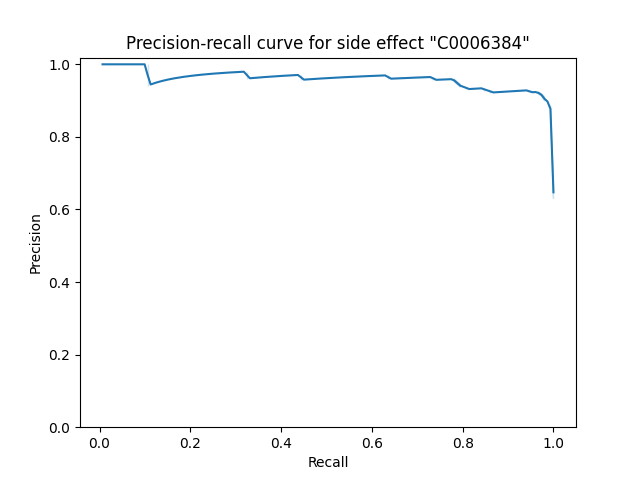

Supplement: btae706_Supplementary_Data [file btae706_supplementary_data.zip › simple_selfloops/figures/C0006384/precision_recall.png]
