# Supplementary figures and images for: Fast polypharmacy side effect prediction using tensor factorization (part 1 of 2)
Source: Bioinformatics. 2024 Nov 25;40(12):btae706. doi: 10.1093/bioinformatics/btae706 (PMC11646082; doi:10.1093/bioinformatics/btae706)

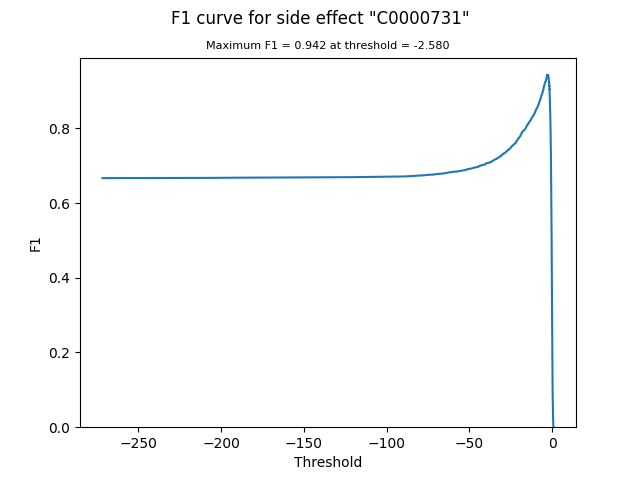

Supplement: btae706_Supplementary_Data [file btae706_supplementary_data.zip › simple_selfloops/figures/C0000731/F1_curve.png]

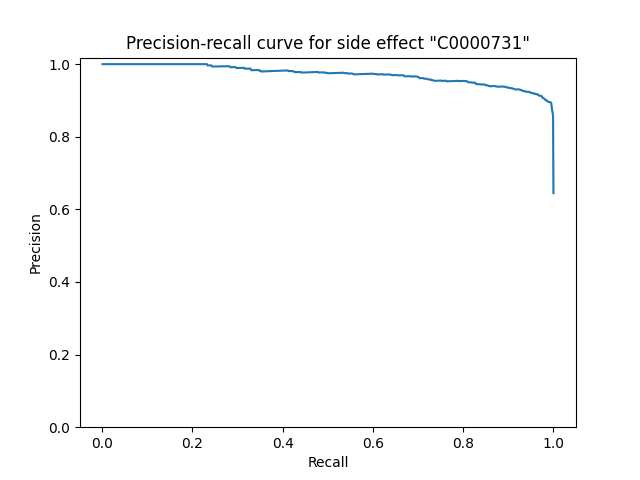

Supplement: btae706_Supplementary_Data [file btae706_supplementary_data.zip › simple_selfloops/figures/C0000731/precision_recall.png]

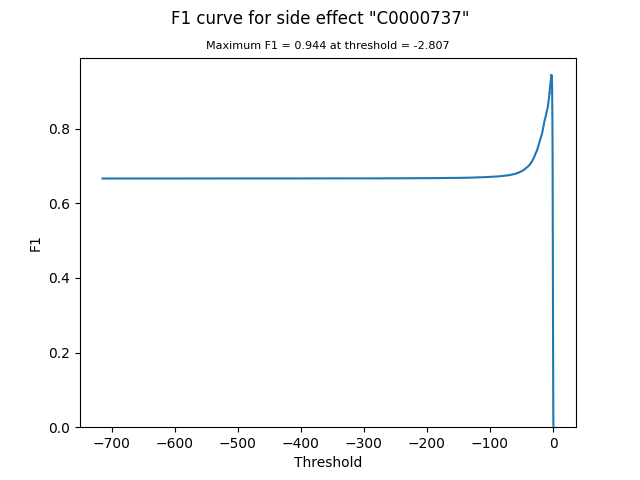

Supplement: btae706_Supplementary_Data [file btae706_supplementary_data.zip › simple_selfloops/figures/C0000737/F1_curve.png]

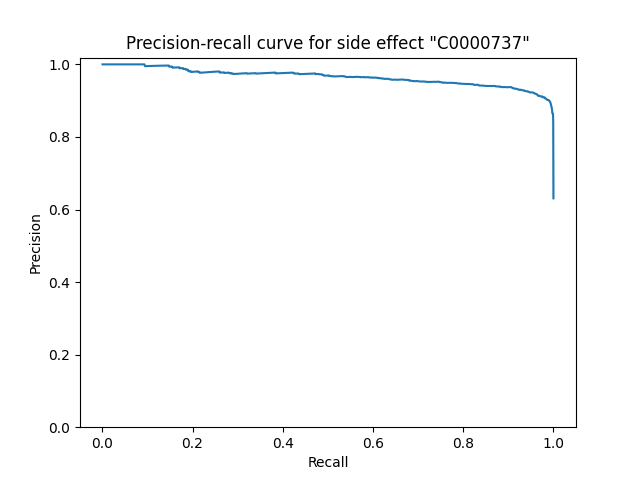

Supplement: btae706_Supplementary_Data [file btae706_supplementary_data.zip › simple_selfloops/figures/C0000737/precision_recall.png]

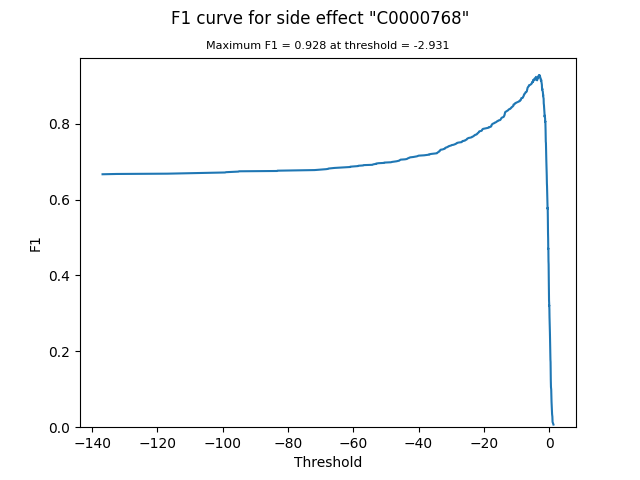

Supplement: btae706_Supplementary_Data [file btae706_supplementary_data.zip › simple_selfloops/figures/C0000768/F1_curve.png]

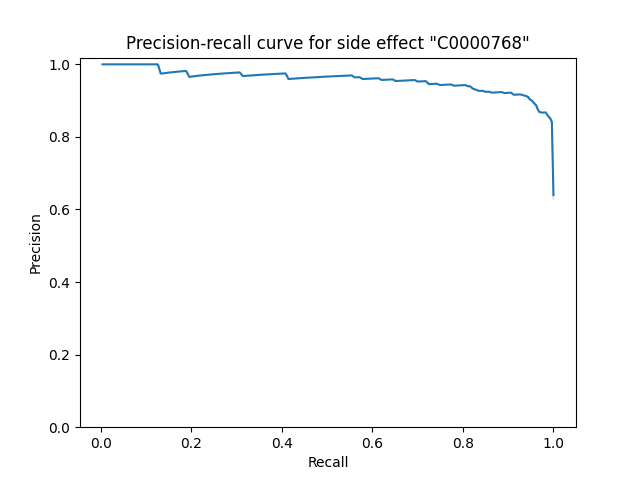

Supplement: btae706_Supplementary_Data [file btae706_supplementary_data.zip › simple_selfloops/figures/C0000768/precision_recall.png]

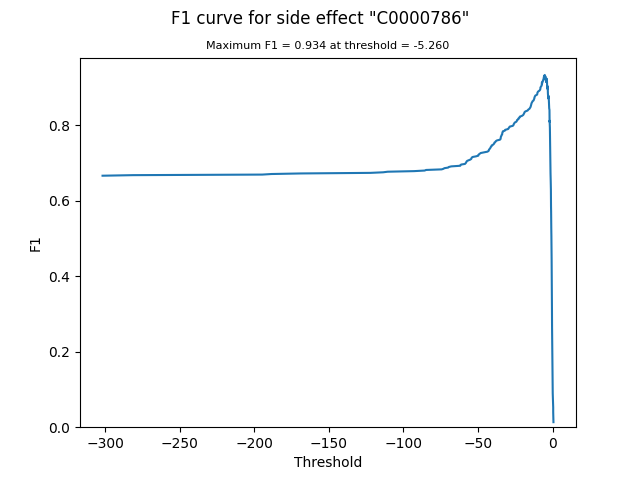

Supplement: btae706_Supplementary_Data [file btae706_supplementary_data.zip › simple_selfloops/figures/C0000786/F1_curve.png]

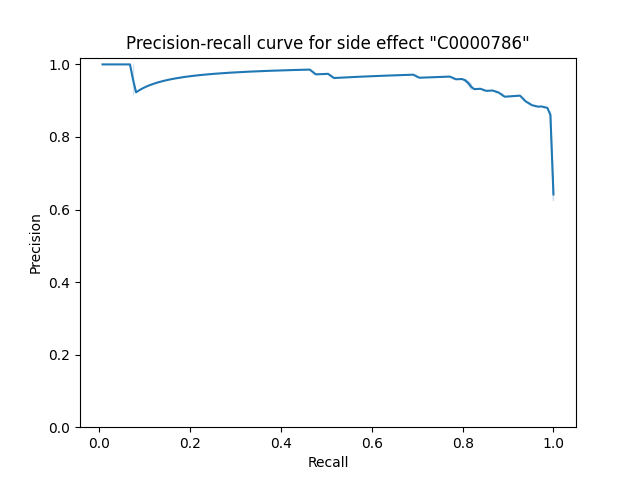

Supplement: btae706_Supplementary_Data [file btae706_supplementary_data.zip › simple_selfloops/figures/C0000786/precision_recall.png]

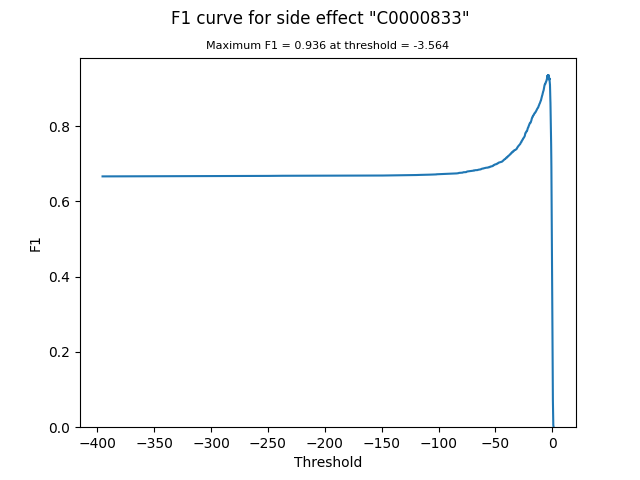

Supplement: btae706_Supplementary_Data [file btae706_supplementary_data.zip › simple_selfloops/figures/C0000833/F1_curve.png]

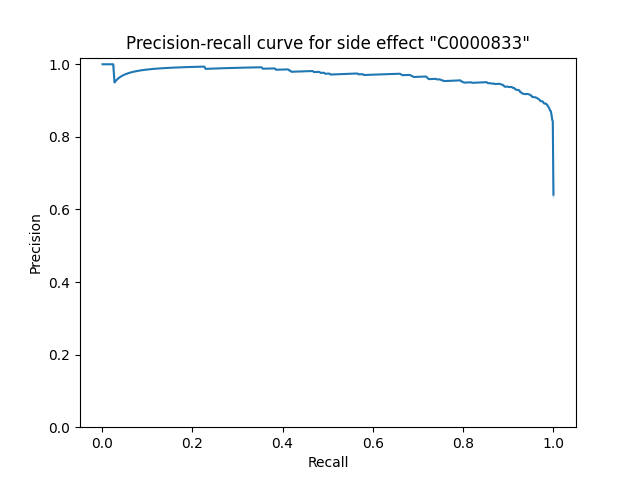

Supplement: btae706_Supplementary_Data [file btae706_supplementary_data.zip › simple_selfloops/figures/C0000833/precision_recall.png]

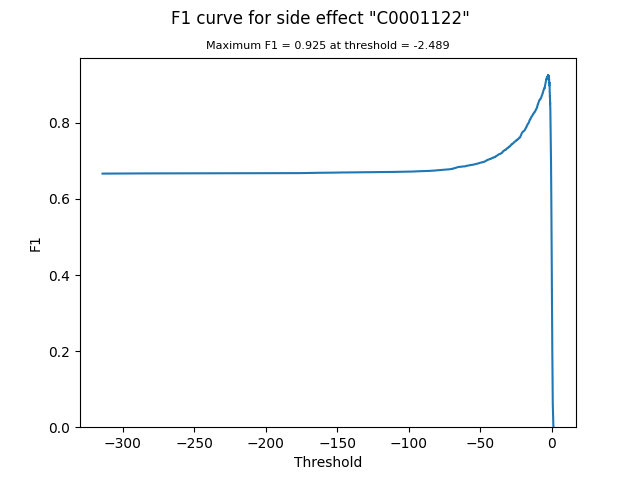

Supplement: btae706_Supplementary_Data [file btae706_supplementary_data.zip › simple_selfloops/figures/C0001122/F1_curve.png]

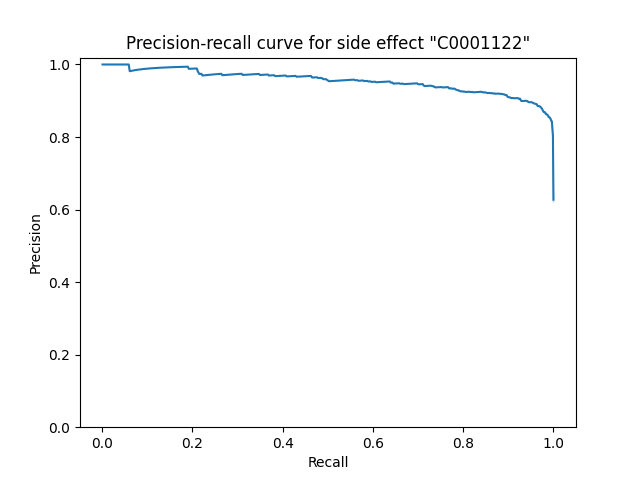

Supplement: btae706_Supplementary_Data [file btae706_supplementary_data.zip › simple_selfloops/figures/C0001122/precision_recall.png]

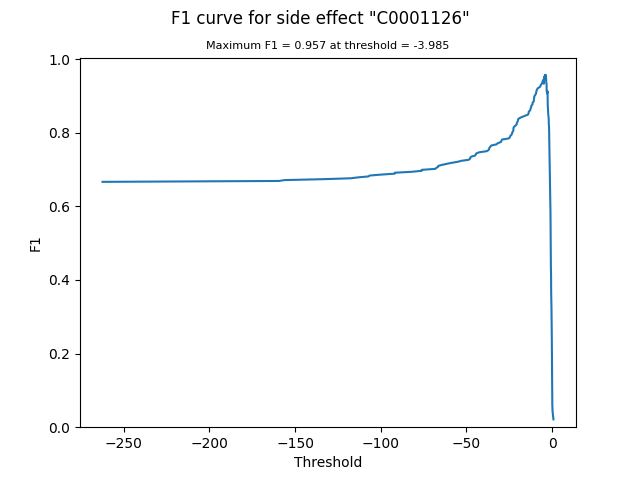

Supplement: btae706_Supplementary_Data [file btae706_supplementary_data.zip › simple_selfloops/figures/C0001126/F1_curve.png]

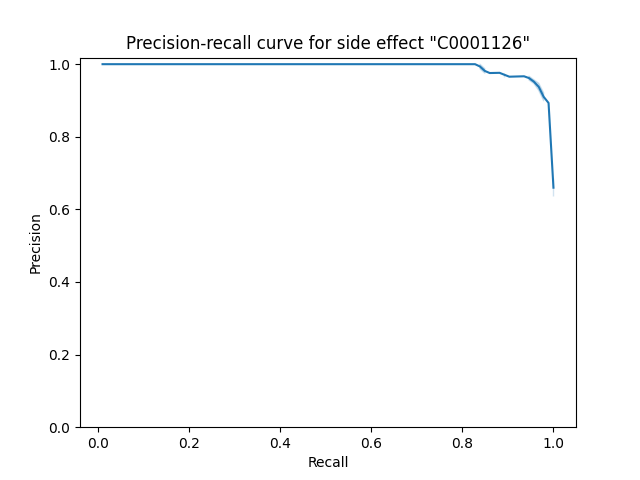

Supplement: btae706_Supplementary_Data [file btae706_supplementary_data.zip › simple_selfloops/figures/C0001126/precision_recall.png]

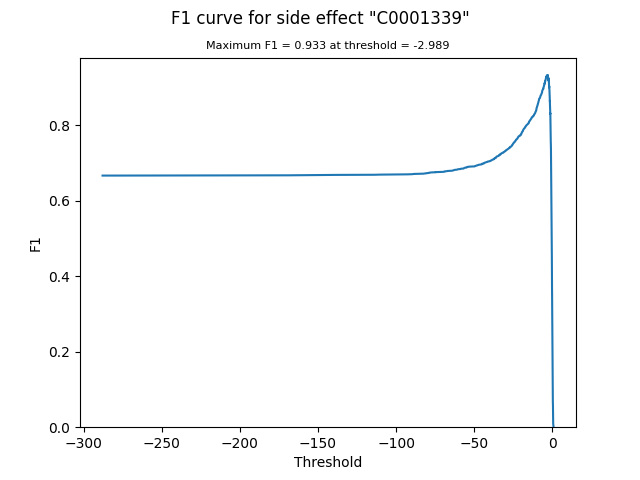

Supplement: btae706_Supplementary_Data [file btae706_supplementary_data.zip › simple_selfloops/figures/C0001339/F1_curve.png]

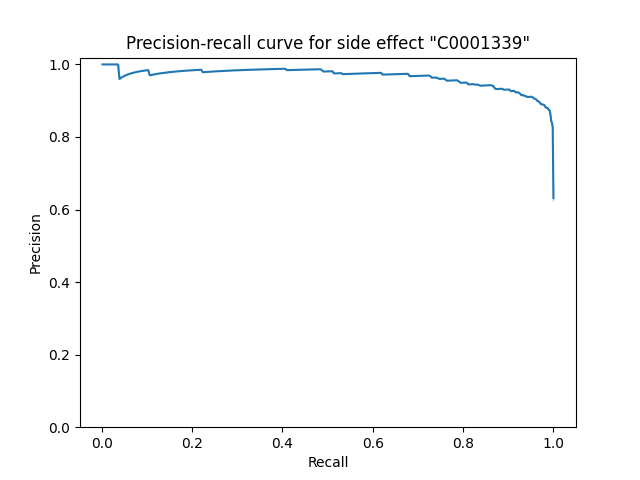

Supplement: btae706_Supplementary_Data [file btae706_supplementary_data.zip › simple_selfloops/figures/C0001339/precision_recall.png]

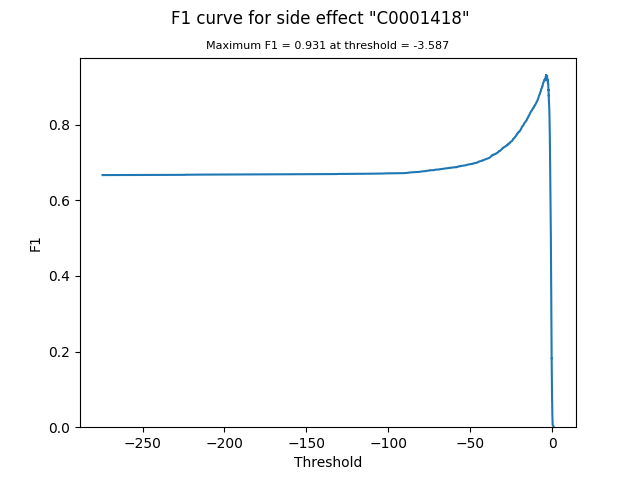

Supplement: btae706_Supplementary_Data [file btae706_supplementary_data.zip › simple_selfloops/figures/C0001418/F1_curve.png]

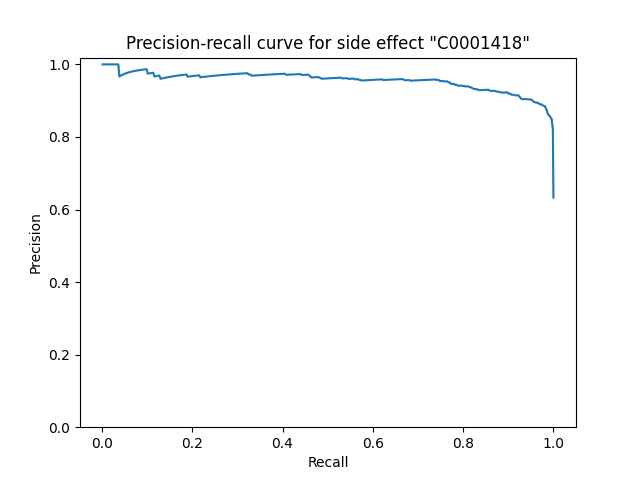

Supplement: btae706_Supplementary_Data [file btae706_supplementary_data.zip › simple_selfloops/figures/C0001418/precision_recall.png]

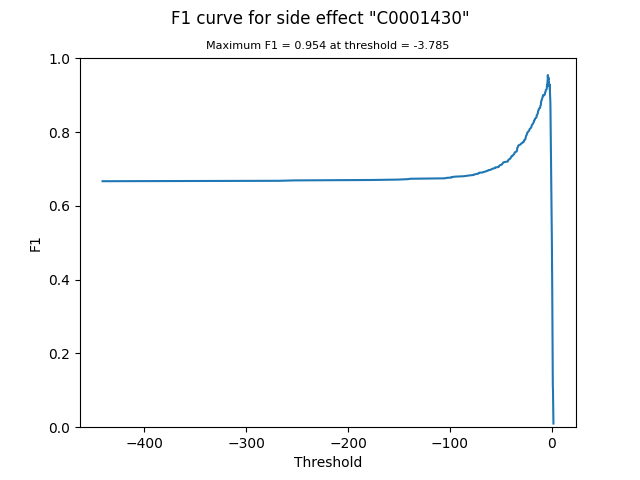

Supplement: btae706_Supplementary_Data [file btae706_supplementary_data.zip › simple_selfloops/figures/C0001430/F1_curve.png]

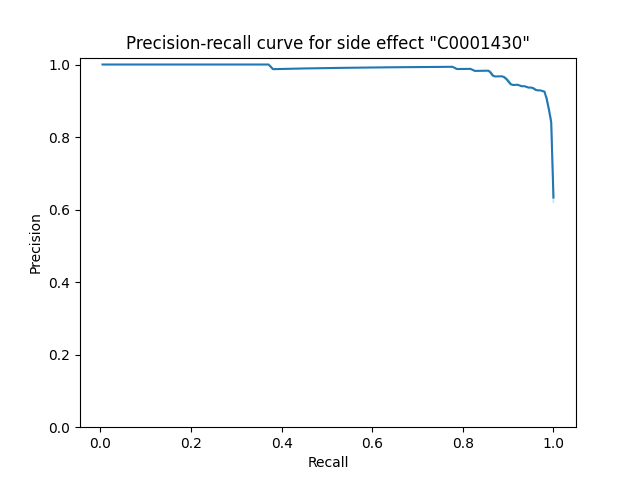

Supplement: btae706_Supplementary_Data [file btae706_supplementary_data.zip › simple_selfloops/figures/C0001430/precision_recall.png]

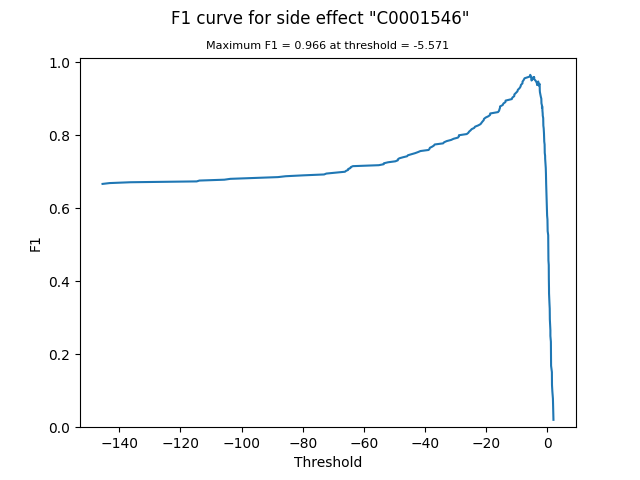

Supplement: btae706_Supplementary_Data [file btae706_supplementary_data.zip › simple_selfloops/figures/C0001546/F1_curve.png]

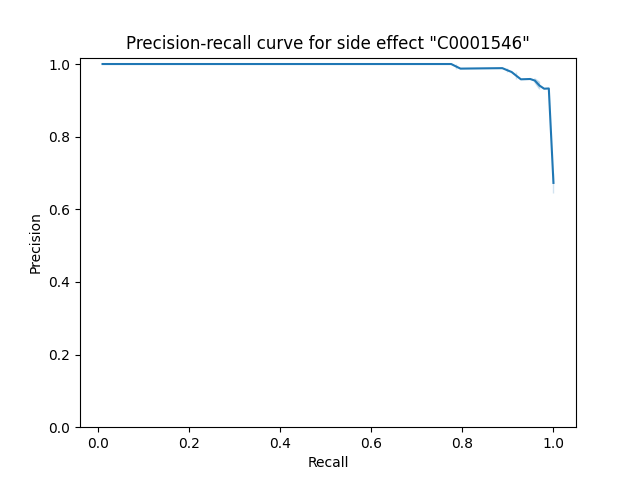

Supplement: btae706_Supplementary_Data [file btae706_supplementary_data.zip › simple_selfloops/figures/C0001546/precision_recall.png]

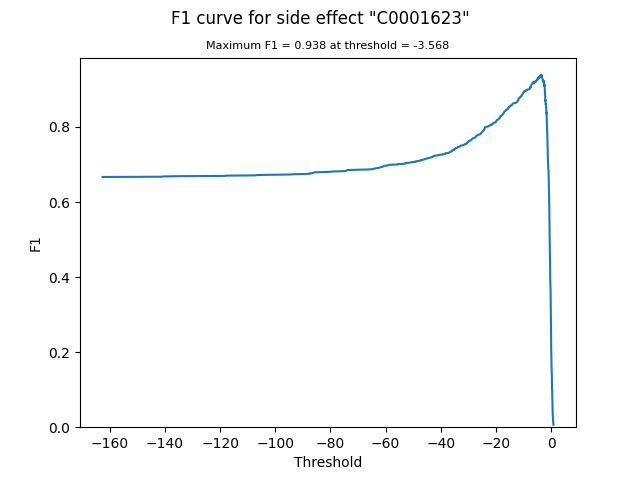

Supplement: btae706_Supplementary_Data [file btae706_supplementary_data.zip › simple_selfloops/figures/C0001623/F1_curve.png]

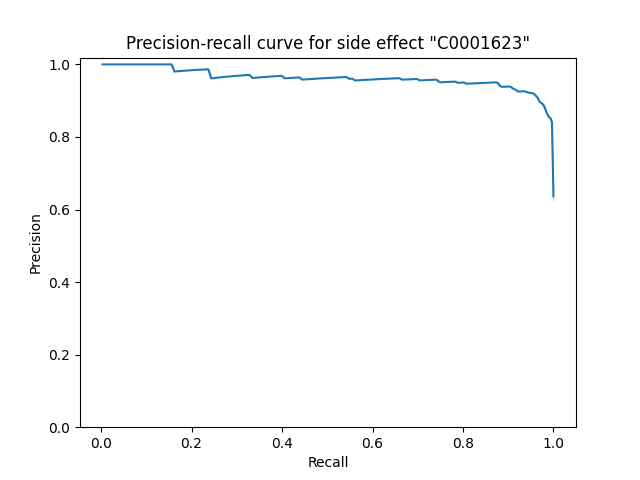

Supplement: btae706_Supplementary_Data [file btae706_supplementary_data.zip › simple_selfloops/figures/C0001623/precision_recall.png]

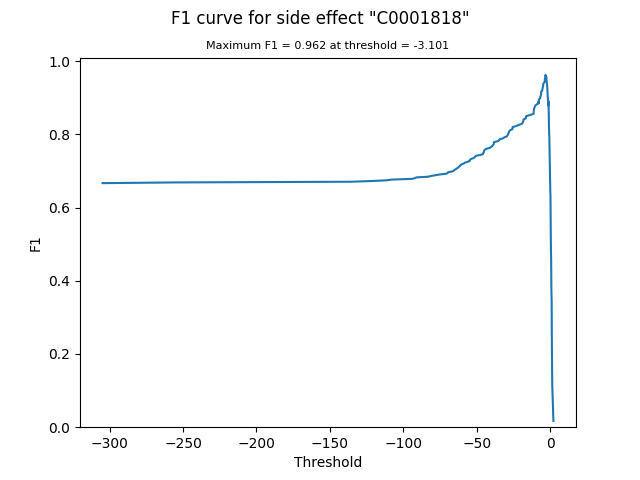

Supplement: btae706_Supplementary_Data [file btae706_supplementary_data.zip › simple_selfloops/figures/C0001818/F1_curve.png]

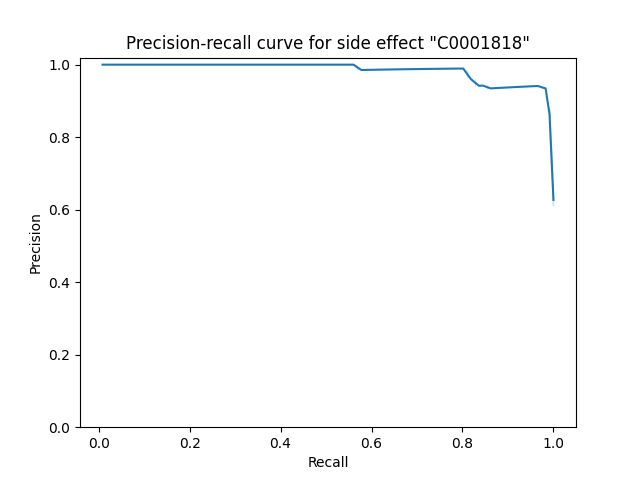

Supplement: btae706_Supplementary_Data [file btae706_supplementary_data.zip › simple_selfloops/figures/C0001818/precision_recall.png]

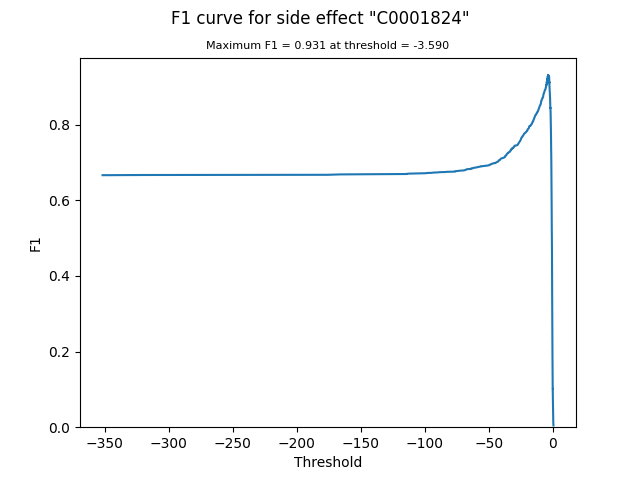

Supplement: btae706_Supplementary_Data [file btae706_supplementary_data.zip › simple_selfloops/figures/C0001824/F1_curve.png]

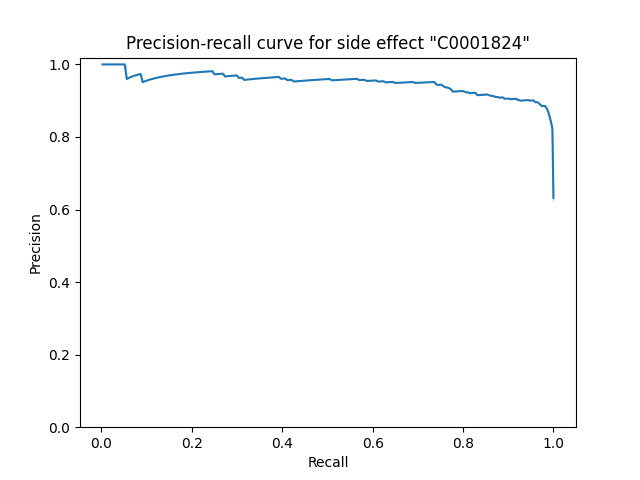

Supplement: btae706_Supplementary_Data [file btae706_supplementary_data.zip › simple_selfloops/figures/C0001824/precision_recall.png]

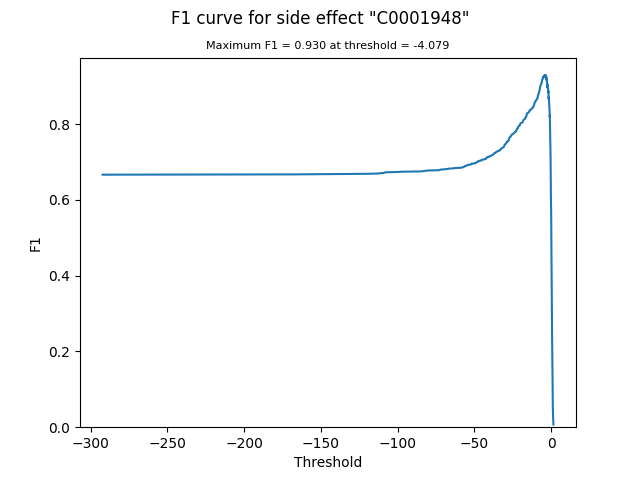

Supplement: btae706_Supplementary_Data [file btae706_supplementary_data.zip › simple_selfloops/figures/C0001948/F1_curve.png]

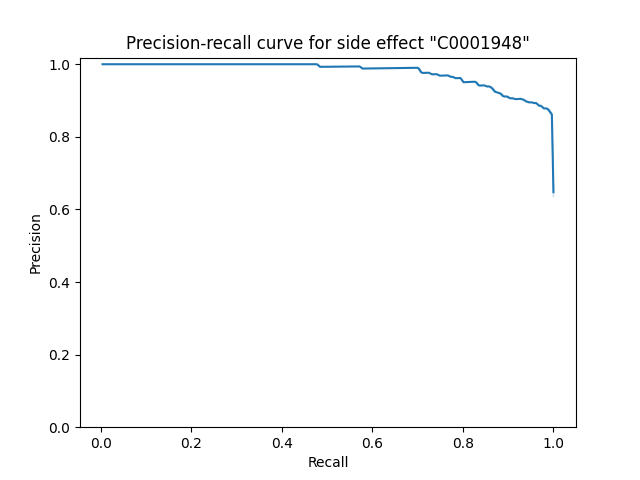

Supplement: btae706_Supplementary_Data [file btae706_supplementary_data.zip › simple_selfloops/figures/C0001948/precision_recall.png]

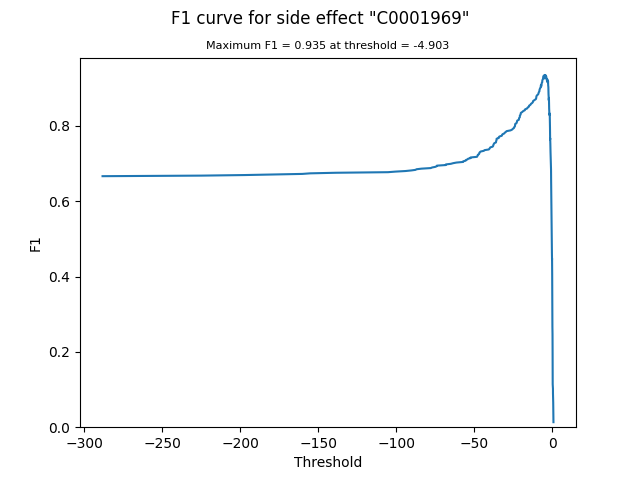

Supplement: btae706_Supplementary_Data [file btae706_supplementary_data.zip › simple_selfloops/figures/C0001969/F1_curve.png]

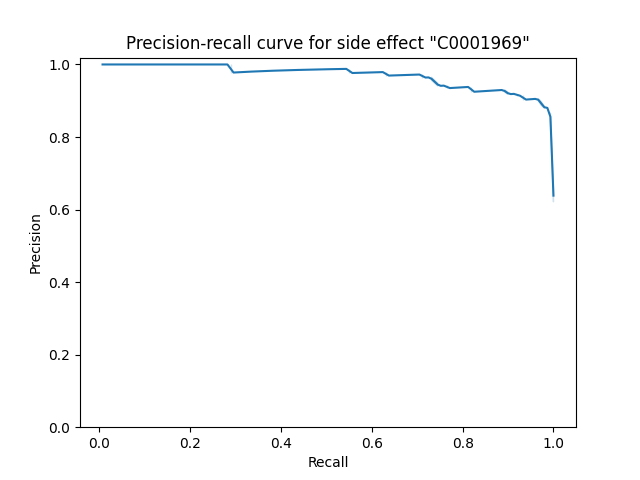

Supplement: btae706_Supplementary_Data [file btae706_supplementary_data.zip › simple_selfloops/figures/C0001969/precision_recall.png]

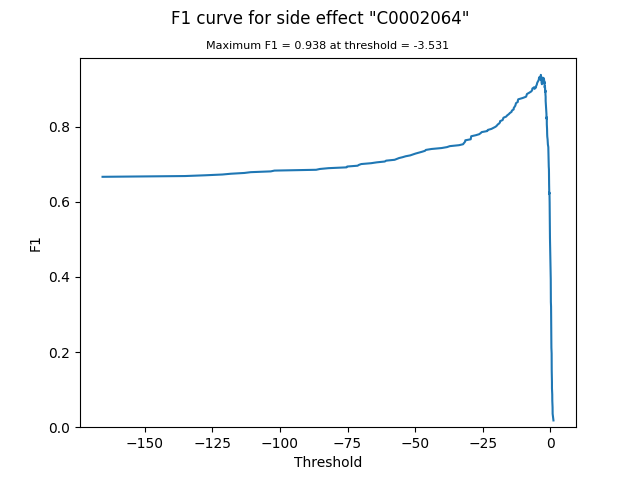

Supplement: btae706_Supplementary_Data [file btae706_supplementary_data.zip › simple_selfloops/figures/C0002064/F1_curve.png]

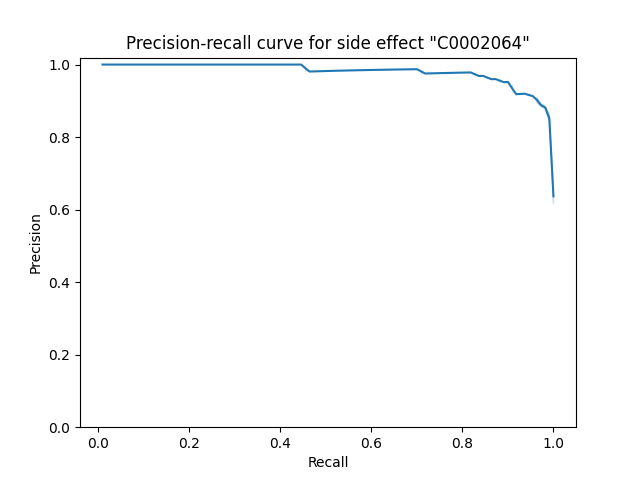

Supplement: btae706_Supplementary_Data [file btae706_supplementary_data.zip › simple_selfloops/figures/C0002064/precision_recall.png]

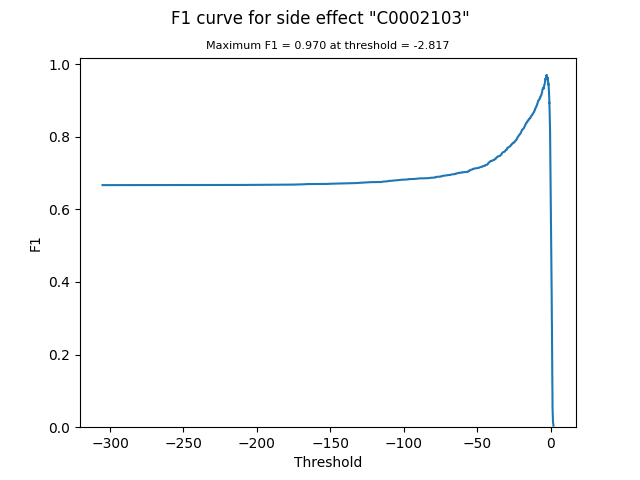

Supplement: btae706_Supplementary_Data [file btae706_supplementary_data.zip › simple_selfloops/figures/C0002103/F1_curve.png]

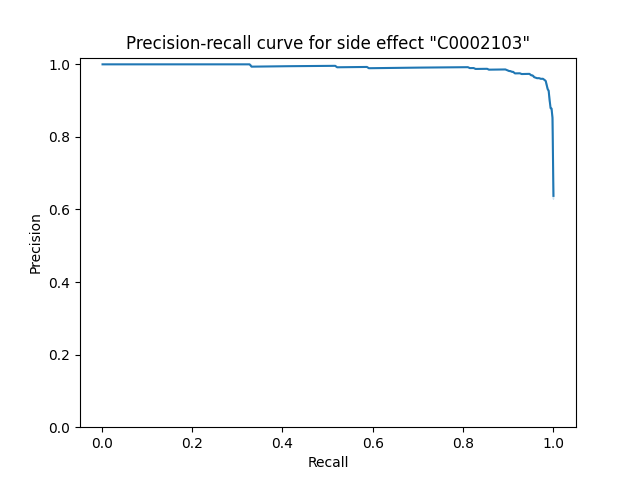

Supplement: btae706_Supplementary_Data [file btae706_supplementary_data.zip › simple_selfloops/figures/C0002103/precision_recall.png]

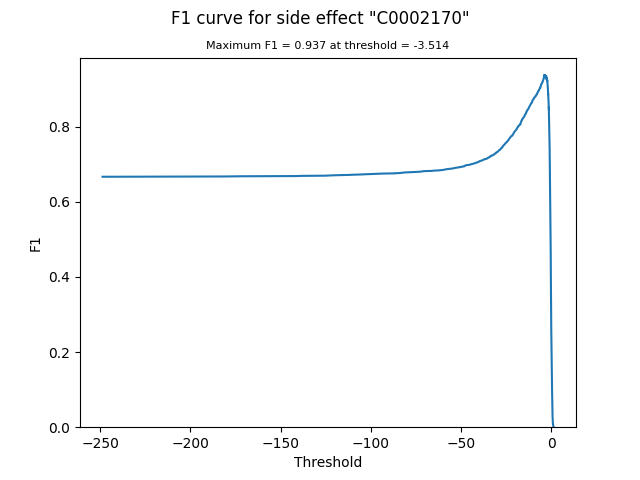

Supplement: btae706_Supplementary_Data [file btae706_supplementary_data.zip › simple_selfloops/figures/C0002170/F1_curve.png]

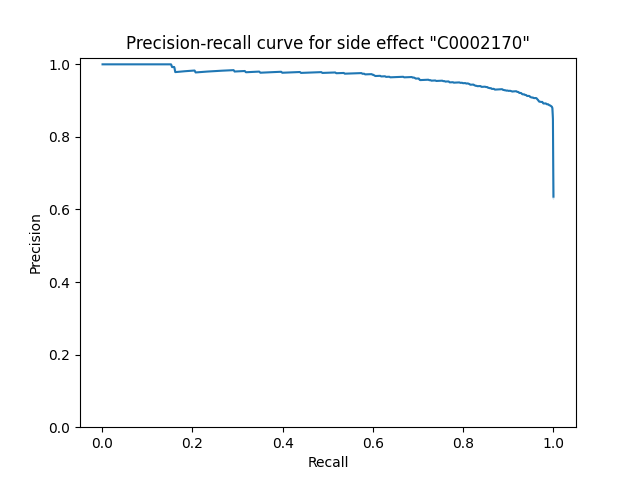

Supplement: btae706_Supplementary_Data [file btae706_supplementary_data.zip › simple_selfloops/figures/C0002170/precision_recall.png]

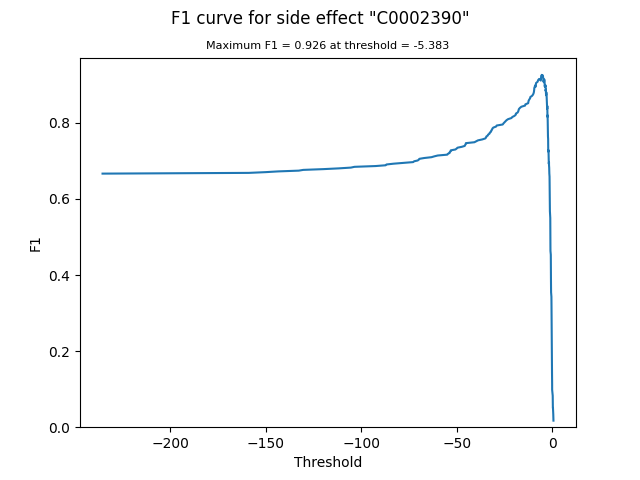

Supplement: btae706_Supplementary_Data [file btae706_supplementary_data.zip › simple_selfloops/figures/C0002390/F1_curve.png]

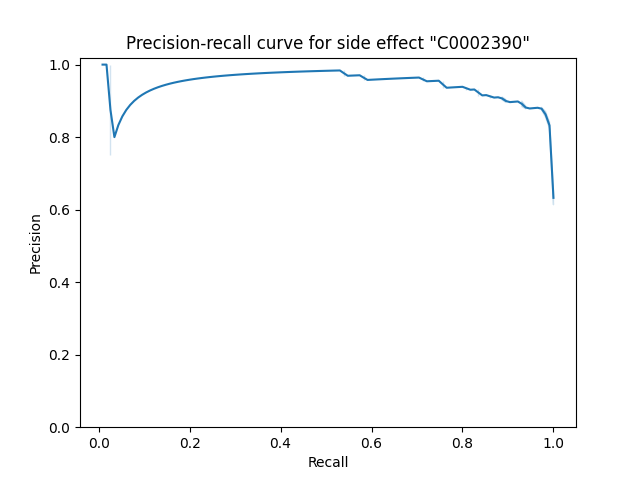

Supplement: btae706_Supplementary_Data [file btae706_supplementary_data.zip › simple_selfloops/figures/C0002390/precision_recall.png]

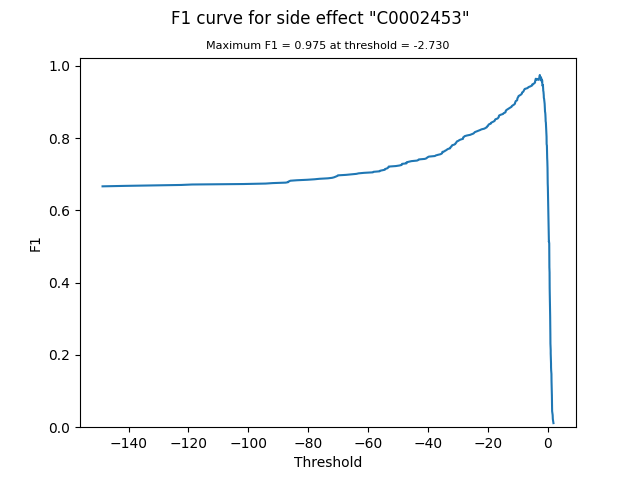

Supplement: btae706_Supplementary_Data [file btae706_supplementary_data.zip › simple_selfloops/figures/C0002453/F1_curve.png]

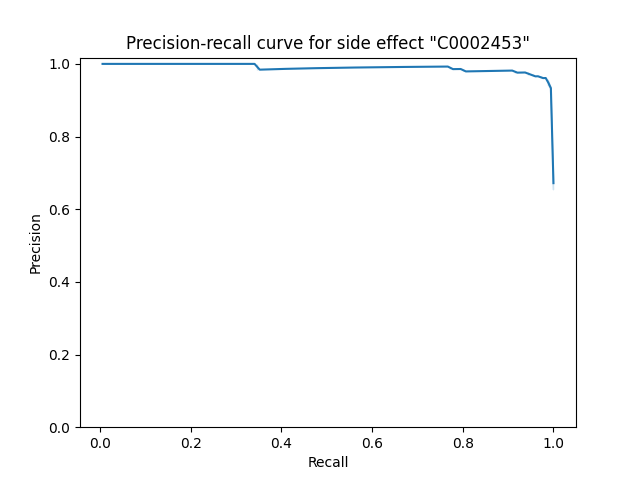

Supplement: btae706_Supplementary_Data [file btae706_supplementary_data.zip › simple_selfloops/figures/C0002453/precision_recall.png]

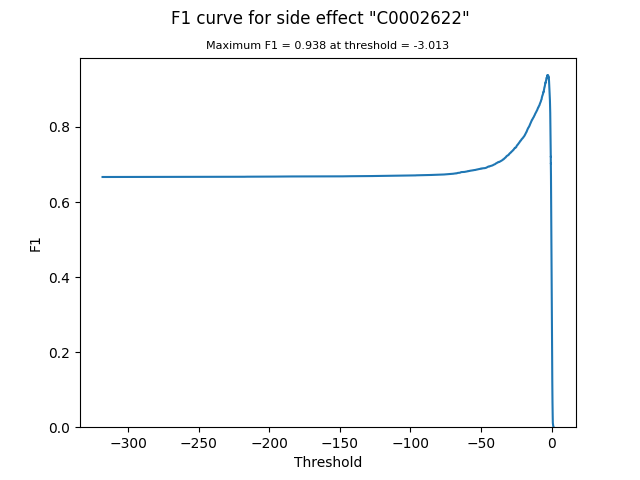

Supplement: btae706_Supplementary_Data [file btae706_supplementary_data.zip › simple_selfloops/figures/C0002622/F1_curve.png]

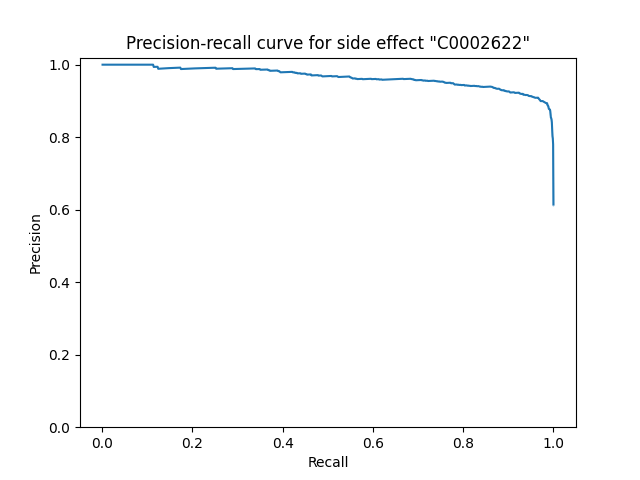

Supplement: btae706_Supplementary_Data [file btae706_supplementary_data.zip › simple_selfloops/figures/C0002622/precision_recall.png]

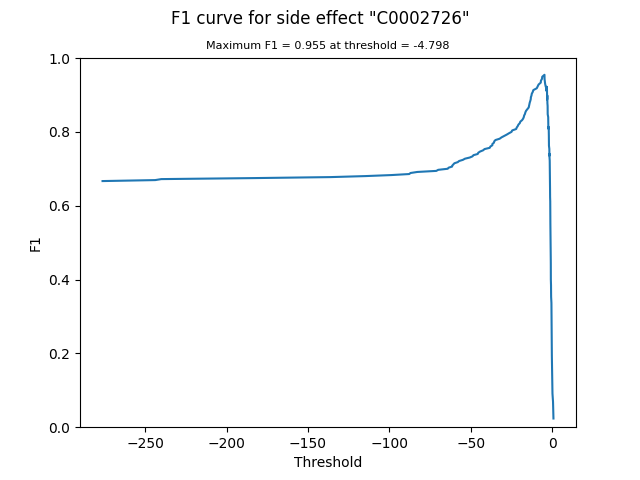

Supplement: btae706_Supplementary_Data [file btae706_supplementary_data.zip › simple_selfloops/figures/C0002726/F1_curve.png]

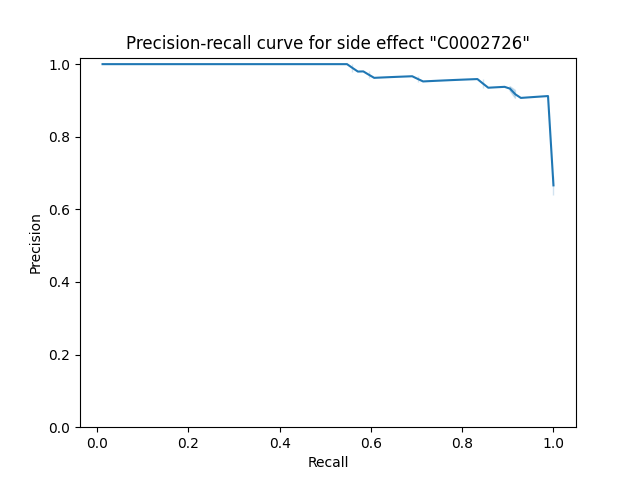

Supplement: btae706_Supplementary_Data [file btae706_supplementary_data.zip › simple_selfloops/figures/C0002726/precision_recall.png]

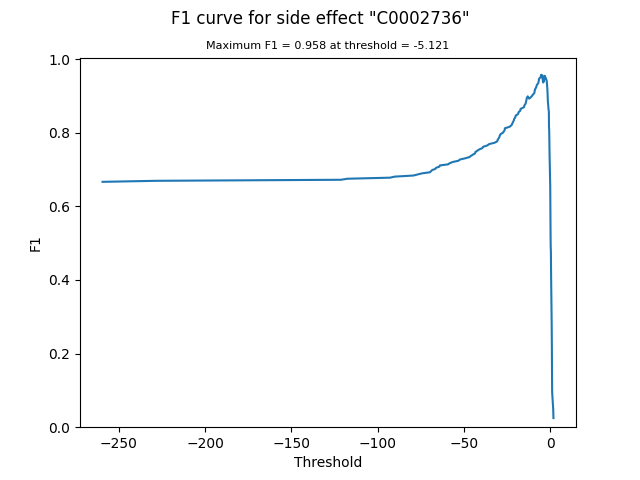

Supplement: btae706_Supplementary_Data [file btae706_supplementary_data.zip › simple_selfloops/figures/C0002736/F1_curve.png]

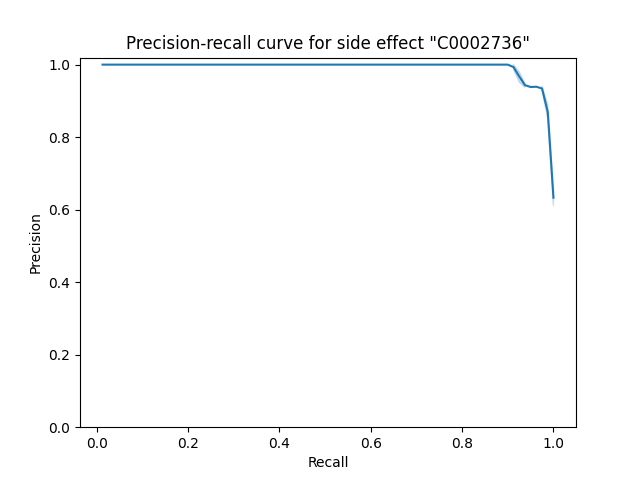

Supplement: btae706_Supplementary_Data [file btae706_supplementary_data.zip › simple_selfloops/figures/C0002736/precision_recall.png]

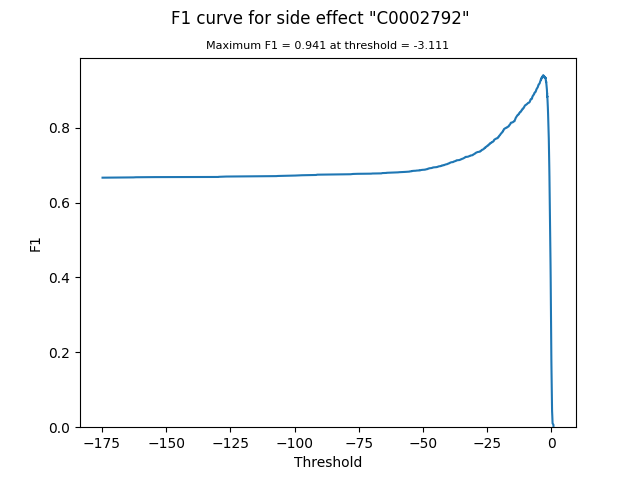

Supplement: btae706_Supplementary_Data [file btae706_supplementary_data.zip › simple_selfloops/figures/C0002792/F1_curve.png]

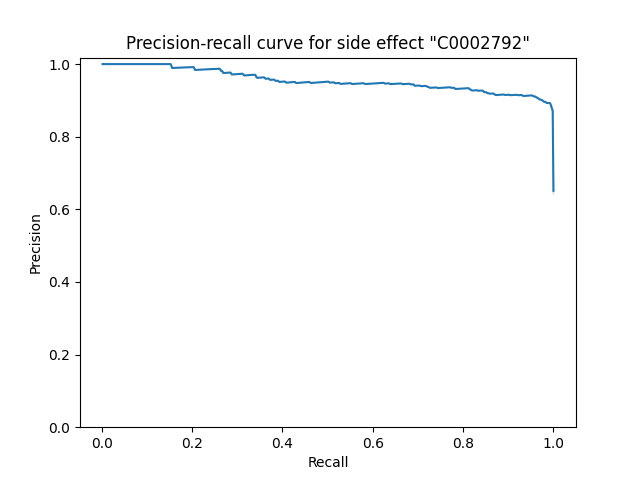

Supplement: btae706_Supplementary_Data [file btae706_supplementary_data.zip › simple_selfloops/figures/C0002792/precision_recall.png]

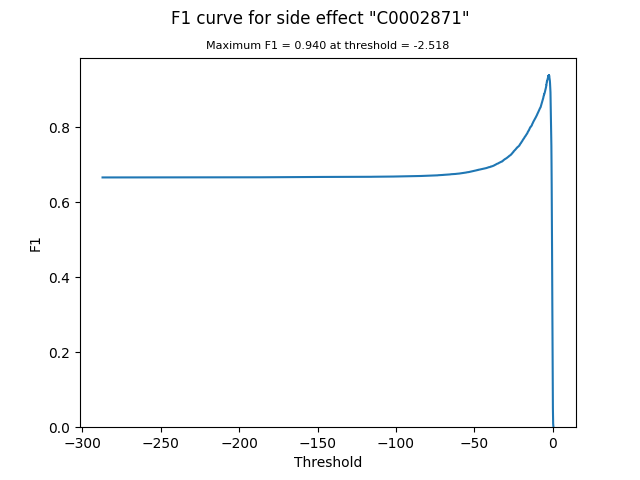

Supplement: btae706_Supplementary_Data [file btae706_supplementary_data.zip › simple_selfloops/figures/C0002871/F1_curve.png]

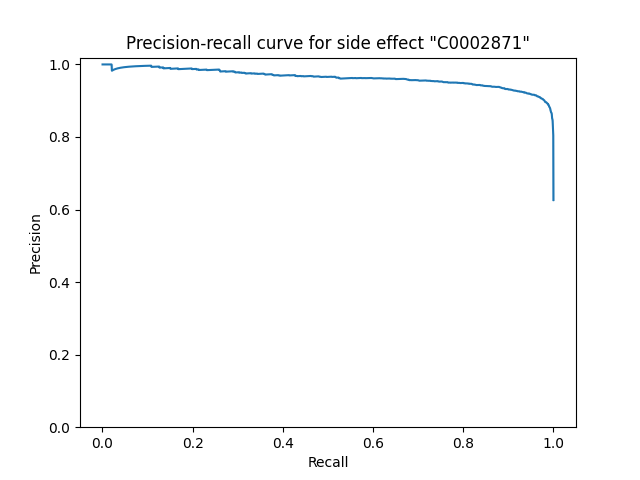

Supplement: btae706_Supplementary_Data [file btae706_supplementary_data.zip › simple_selfloops/figures/C0002871/precision_recall.png]

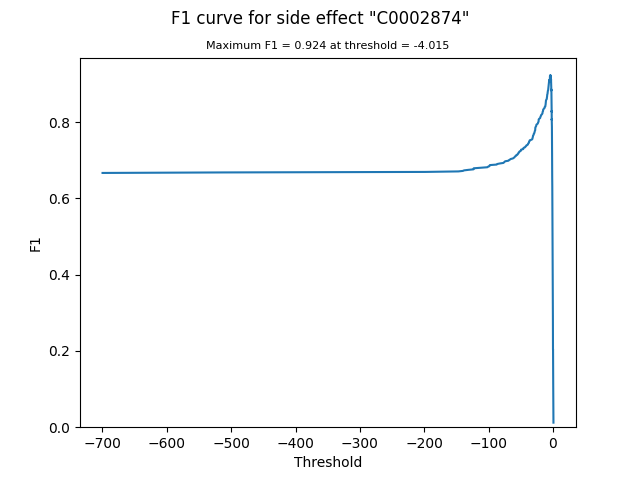

Supplement: btae706_Supplementary_Data [file btae706_supplementary_data.zip › simple_selfloops/figures/C0002874/F1_curve.png]

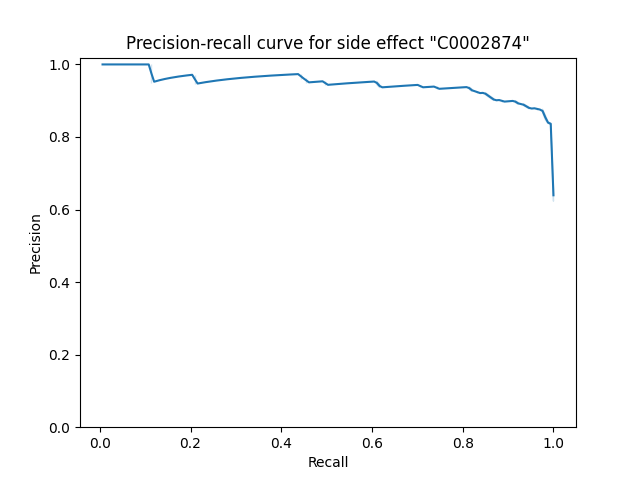

Supplement: btae706_Supplementary_Data [file btae706_supplementary_data.zip › simple_selfloops/figures/C0002874/precision_recall.png]

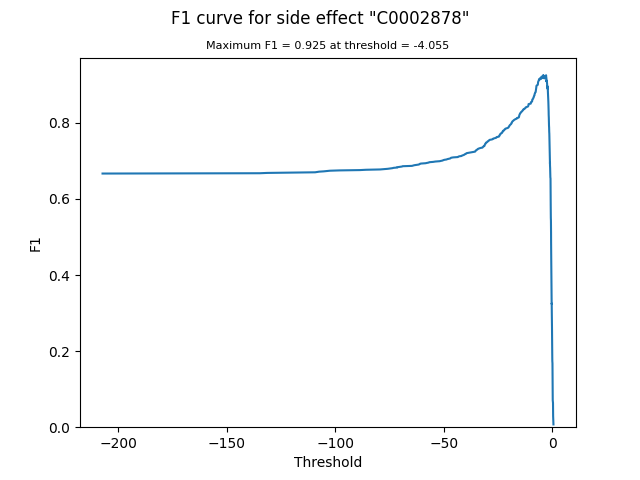

Supplement: btae706_Supplementary_Data [file btae706_supplementary_data.zip › simple_selfloops/figures/C0002878/F1_curve.png]

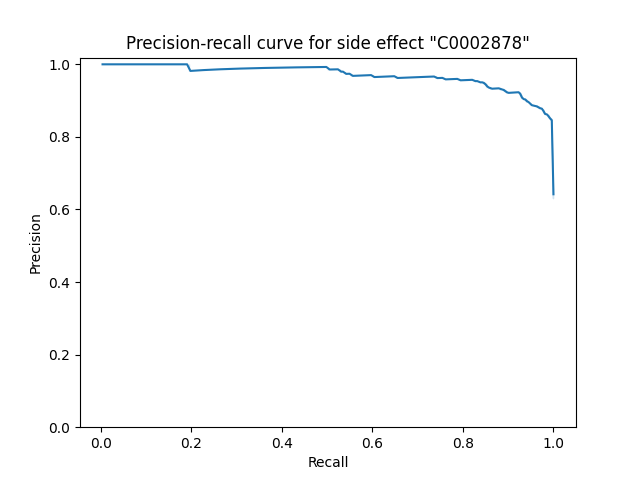

Supplement: btae706_Supplementary_Data [file btae706_supplementary_data.zip › simple_selfloops/figures/C0002878/precision_recall.png]

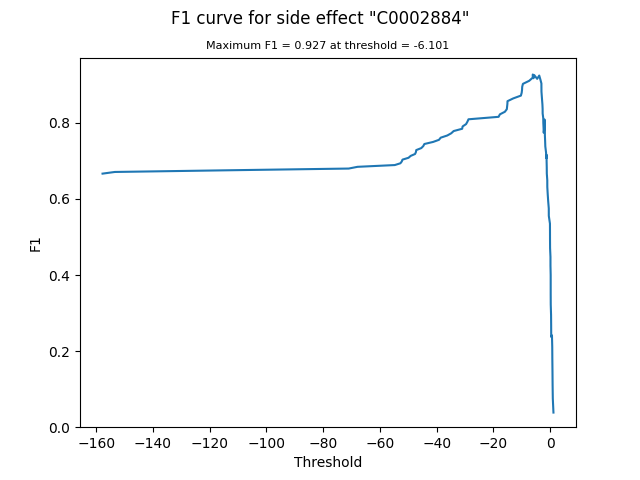

Supplement: btae706_Supplementary_Data [file btae706_supplementary_data.zip › simple_selfloops/figures/C0002884/F1_curve.png]

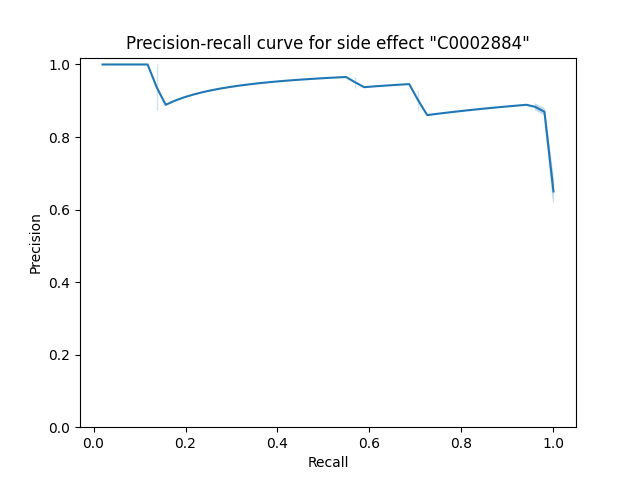

Supplement: btae706_Supplementary_Data [file btae706_supplementary_data.zip › simple_selfloops/figures/C0002884/precision_recall.png]

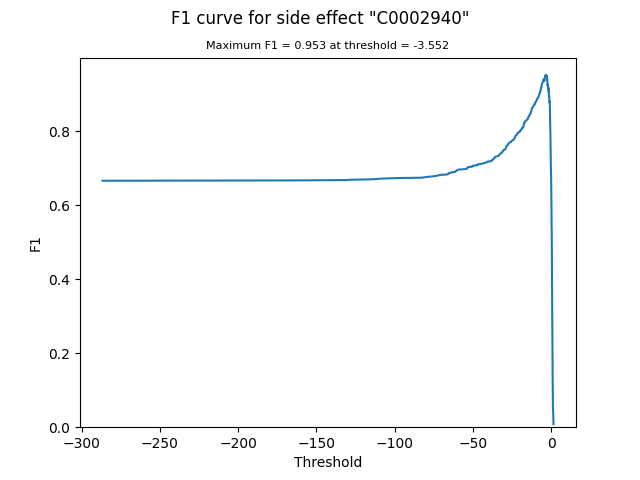

Supplement: btae706_Supplementary_Data [file btae706_supplementary_data.zip › simple_selfloops/figures/C0002940/F1_curve.png]

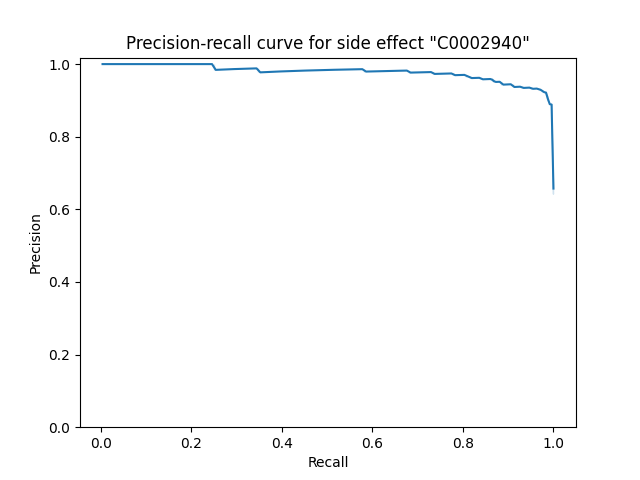

Supplement: btae706_Supplementary_Data [file btae706_supplementary_data.zip › simple_selfloops/figures/C0002940/precision_recall.png]

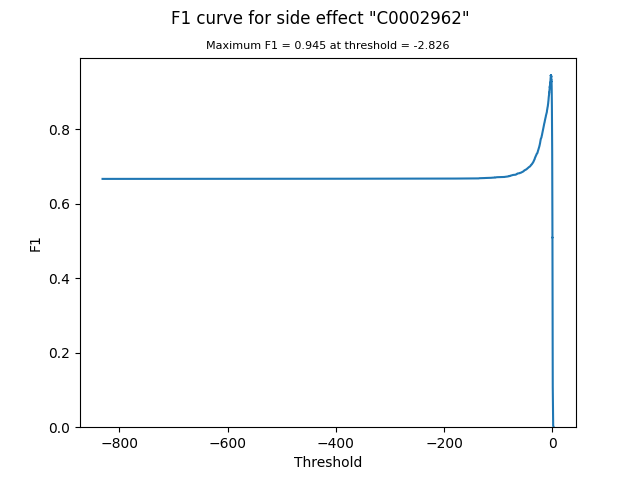

Supplement: btae706_Supplementary_Data [file btae706_supplementary_data.zip › simple_selfloops/figures/C0002962/F1_curve.png]

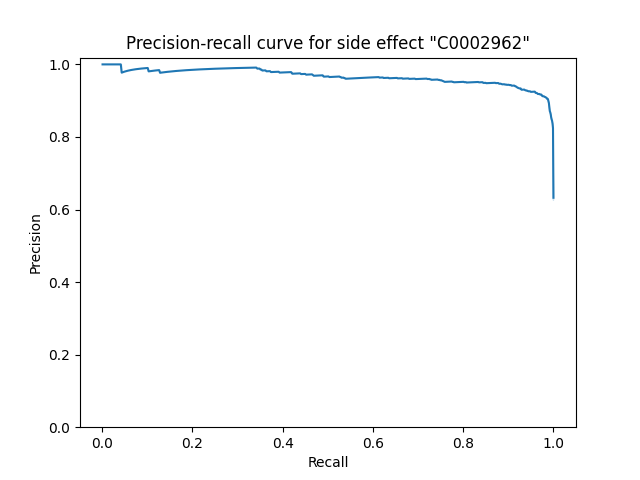

Supplement: btae706_Supplementary_Data [file btae706_supplementary_data.zip › simple_selfloops/figures/C0002962/precision_recall.png]

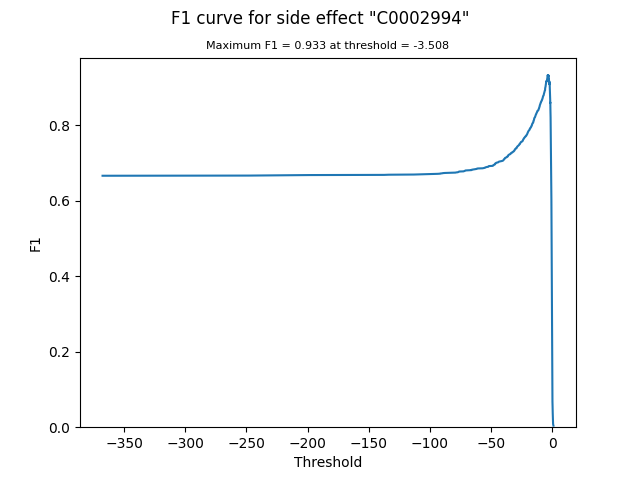

Supplement: btae706_Supplementary_Data [file btae706_supplementary_data.zip › simple_selfloops/figures/C0002994/F1_curve.png]

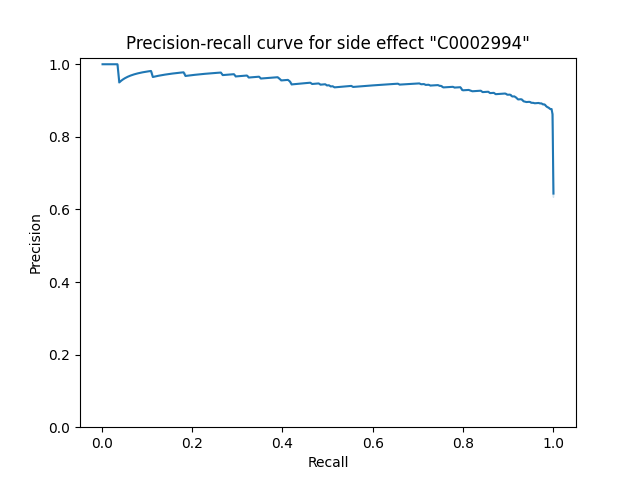

Supplement: btae706_Supplementary_Data [file btae706_supplementary_data.zip › simple_selfloops/figures/C0002994/precision_recall.png]

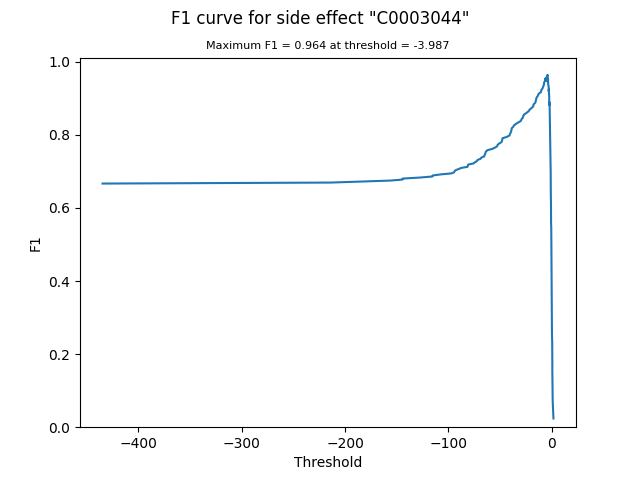

Supplement: btae706_Supplementary_Data [file btae706_supplementary_data.zip › simple_selfloops/figures/C0003044/F1_curve.png]

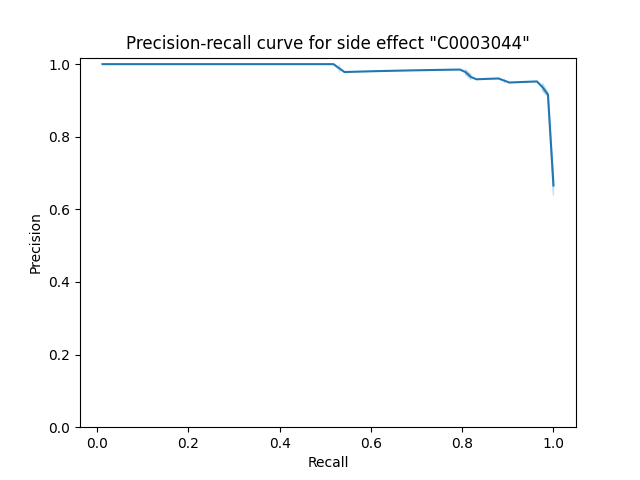

Supplement: btae706_Supplementary_Data [file btae706_supplementary_data.zip › simple_selfloops/figures/C0003044/precision_recall.png]

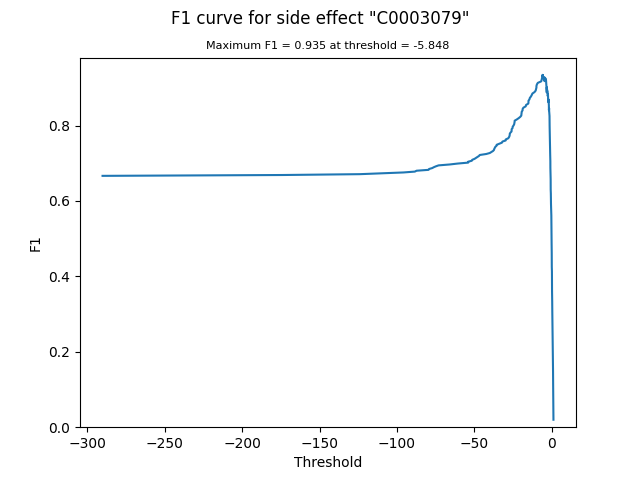

Supplement: btae706_Supplementary_Data [file btae706_supplementary_data.zip › simple_selfloops/figures/C0003079/F1_curve.png]

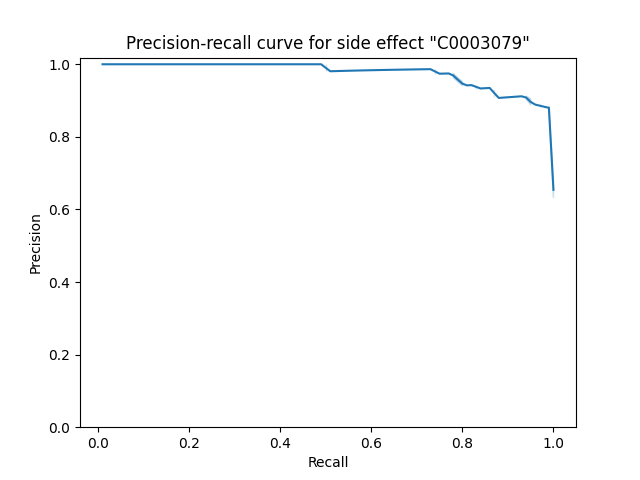

Supplement: btae706_Supplementary_Data [file btae706_supplementary_data.zip › simple_selfloops/figures/C0003079/precision_recall.png]

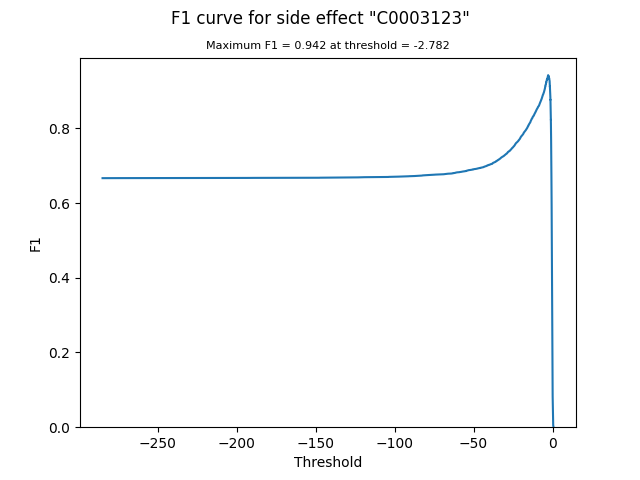

Supplement: btae706_Supplementary_Data [file btae706_supplementary_data.zip › simple_selfloops/figures/C0003123/F1_curve.png]

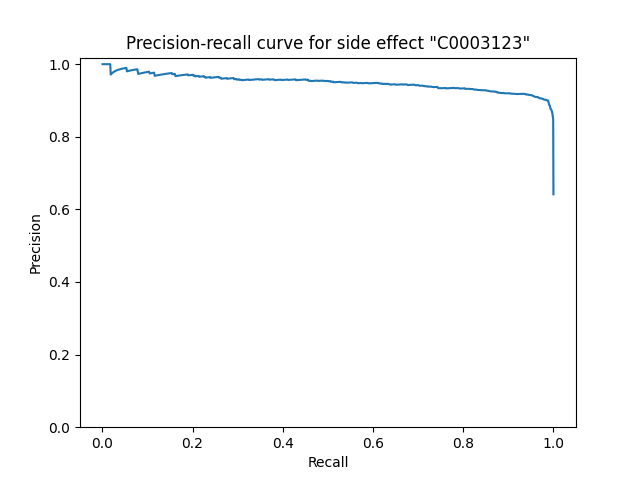

Supplement: btae706_Supplementary_Data [file btae706_supplementary_data.zip › simple_selfloops/figures/C0003123/precision_recall.png]

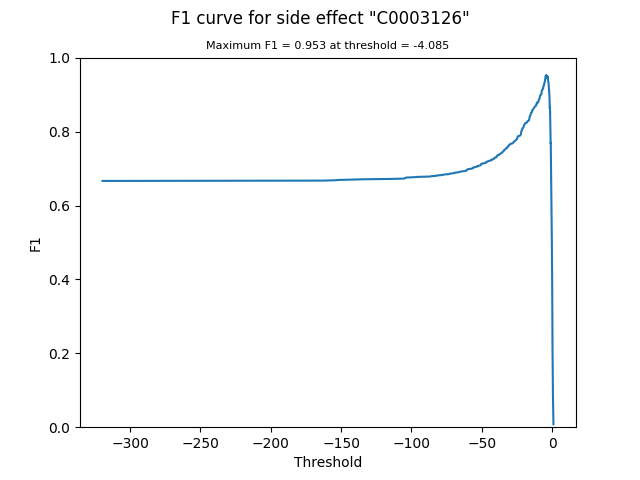

Supplement: btae706_Supplementary_Data [file btae706_supplementary_data.zip › simple_selfloops/figures/C0003126/F1_curve.png]

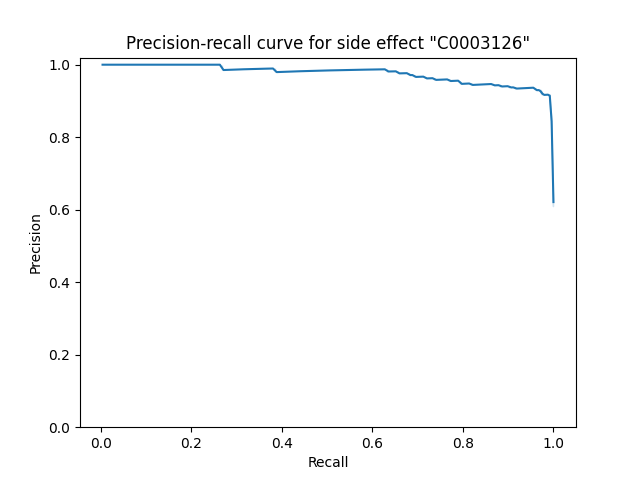

Supplement: btae706_Supplementary_Data [file btae706_supplementary_data.zip › simple_selfloops/figures/C0003126/precision_recall.png]

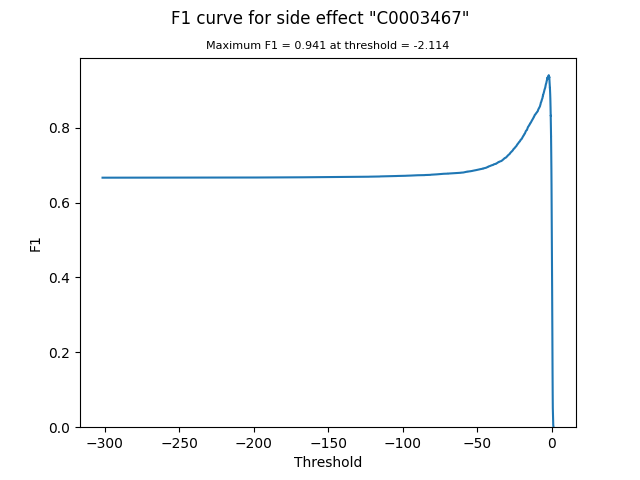

Supplement: btae706_Supplementary_Data [file btae706_supplementary_data.zip › simple_selfloops/figures/C0003467/F1_curve.png]

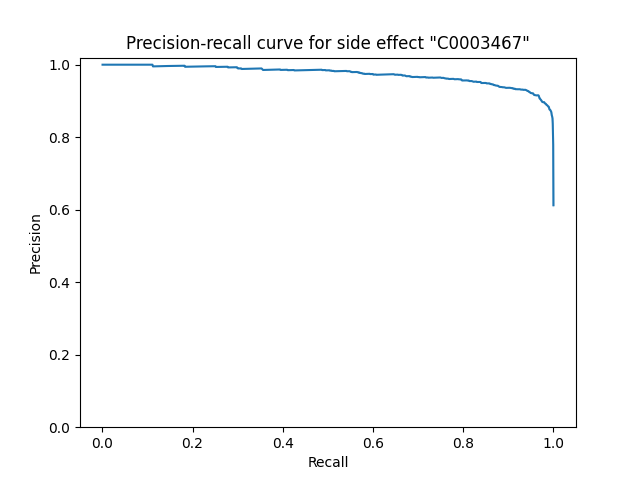

Supplement: btae706_Supplementary_Data [file btae706_supplementary_data.zip › simple_selfloops/figures/C0003467/precision_recall.png]

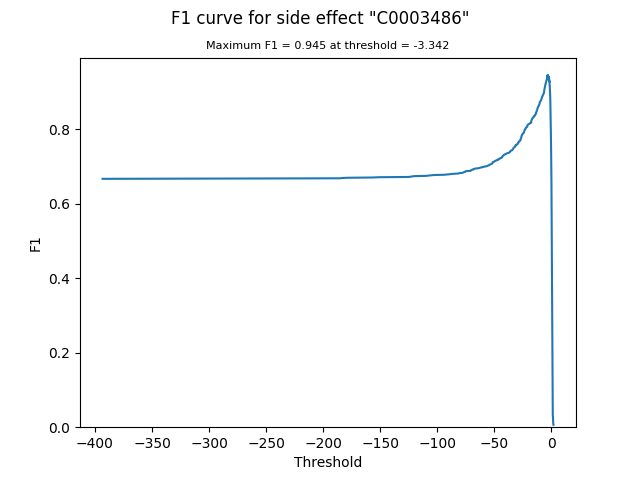

Supplement: btae706_Supplementary_Data [file btae706_supplementary_data.zip › simple_selfloops/figures/C0003486/F1_curve.png]

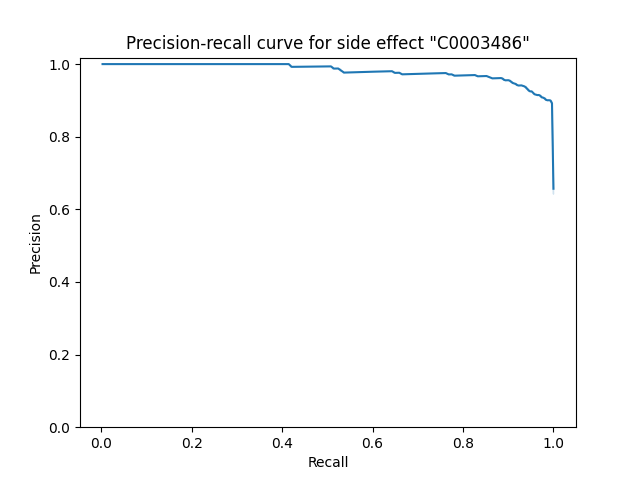

Supplement: btae706_Supplementary_Data [file btae706_supplementary_data.zip › simple_selfloops/figures/C0003486/precision_recall.png]

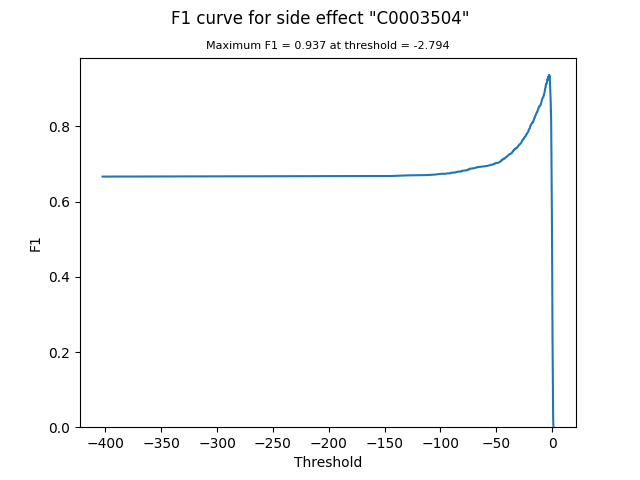

Supplement: btae706_Supplementary_Data [file btae706_supplementary_data.zip › simple_selfloops/figures/C0003504/F1_curve.png]

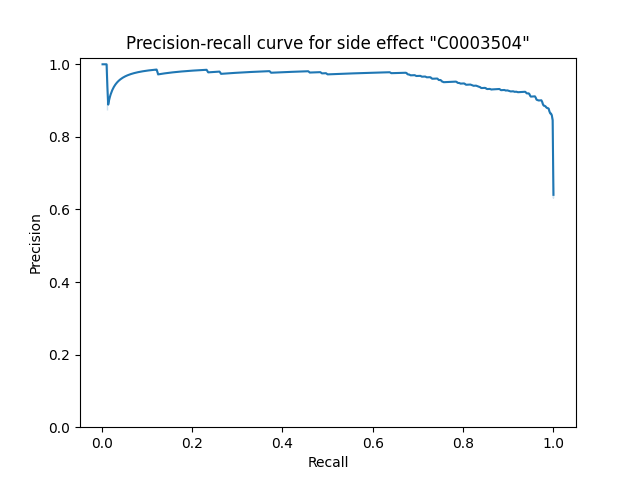

Supplement: btae706_Supplementary_Data [file btae706_supplementary_data.zip › simple_selfloops/figures/C0003504/precision_recall.png]

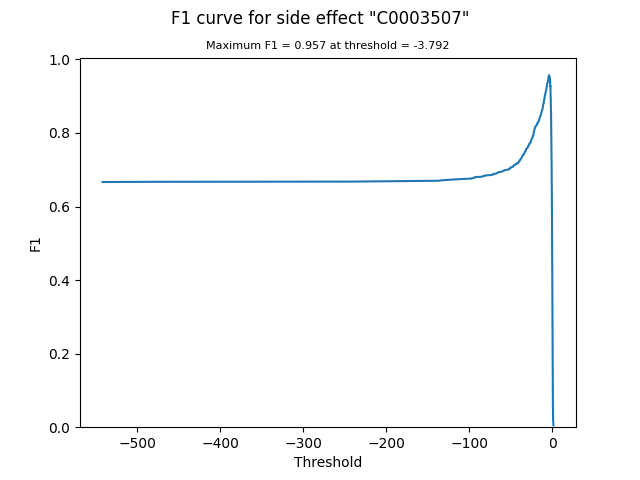

Supplement: btae706_Supplementary_Data [file btae706_supplementary_data.zip › simple_selfloops/figures/C0003507/F1_curve.png]

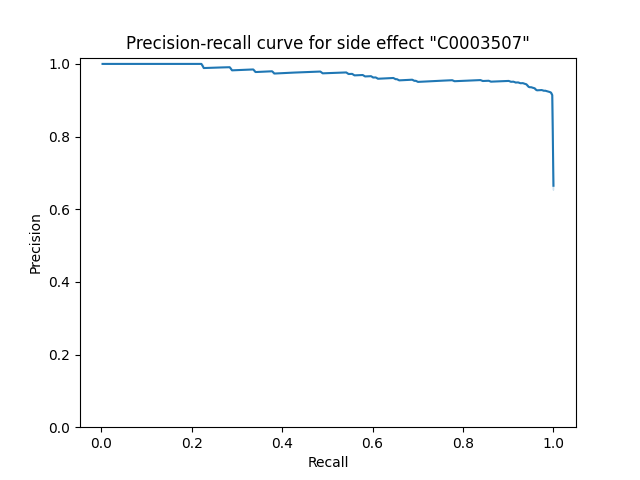

Supplement: btae706_Supplementary_Data [file btae706_supplementary_data.zip › simple_selfloops/figures/C0003507/precision_recall.png]

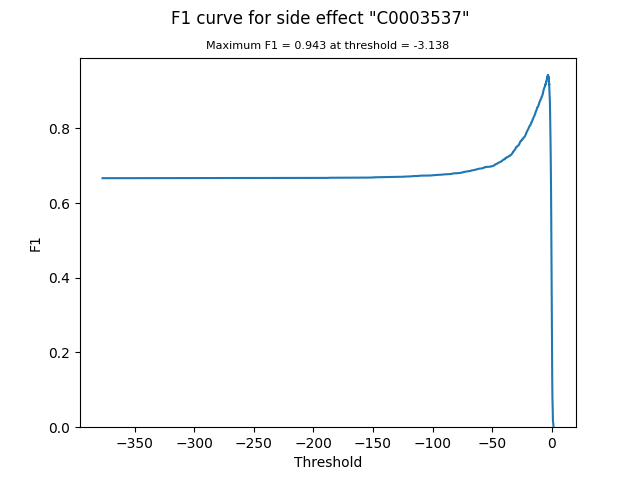

Supplement: btae706_Supplementary_Data [file btae706_supplementary_data.zip › simple_selfloops/figures/C0003537/F1_curve.png]

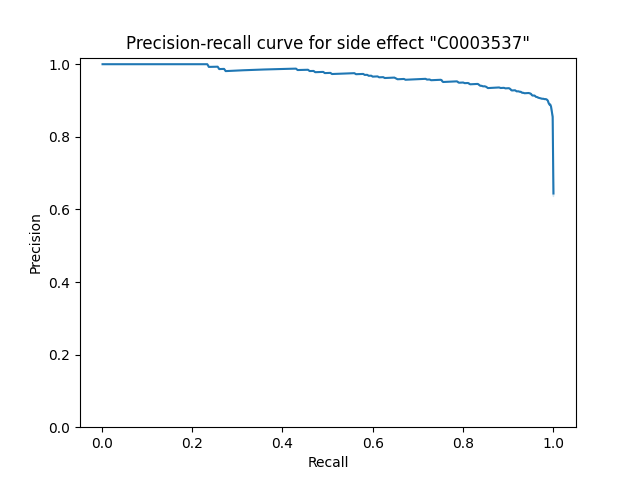

Supplement: btae706_Supplementary_Data [file btae706_supplementary_data.zip › simple_selfloops/figures/C0003537/precision_recall.png]

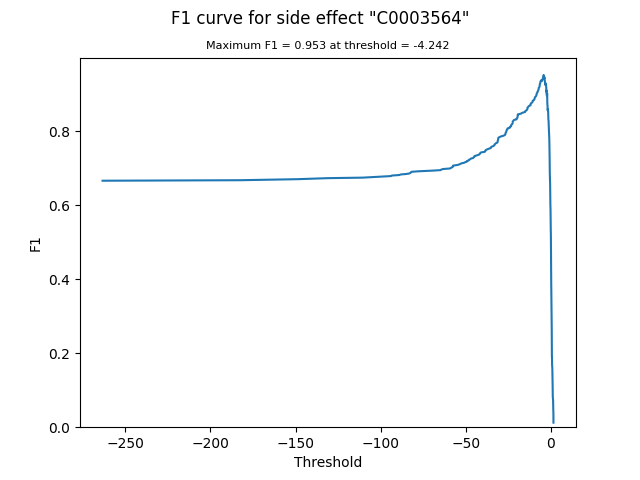

Supplement: btae706_Supplementary_Data [file btae706_supplementary_data.zip › simple_selfloops/figures/C0003564/F1_curve.png]

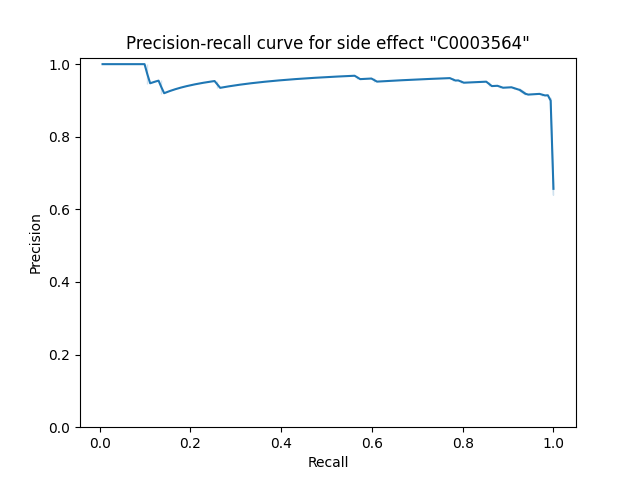

Supplement: btae706_Supplementary_Data [file btae706_supplementary_data.zip › simple_selfloops/figures/C0003564/precision_recall.png]

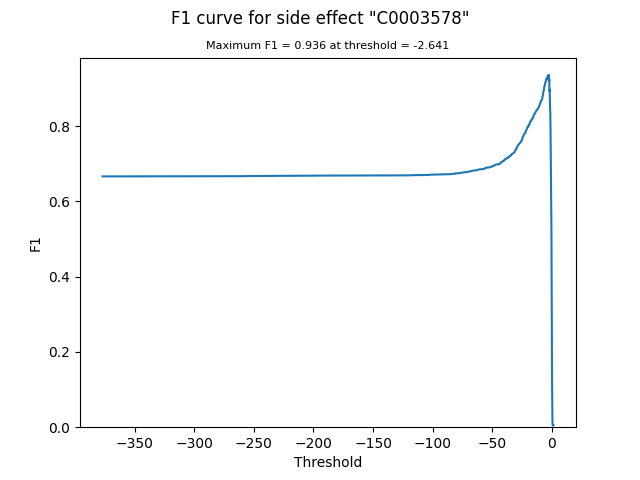

Supplement: btae706_Supplementary_Data [file btae706_supplementary_data.zip › simple_selfloops/figures/C0003578/F1_curve.png]

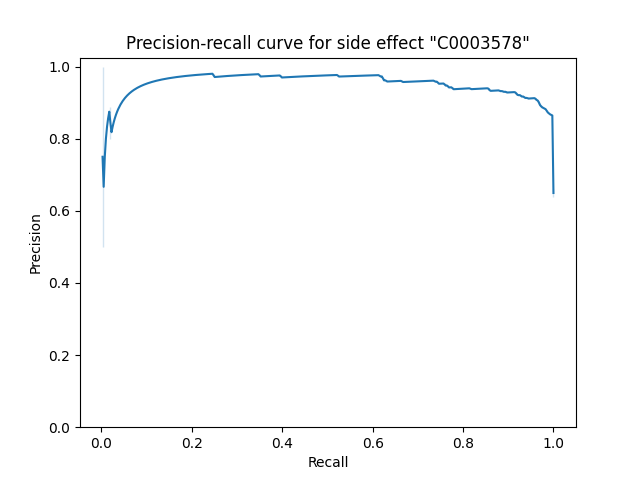

Supplement: btae706_Supplementary_Data [file btae706_supplementary_data.zip › simple_selfloops/figures/C0003578/precision_recall.png]

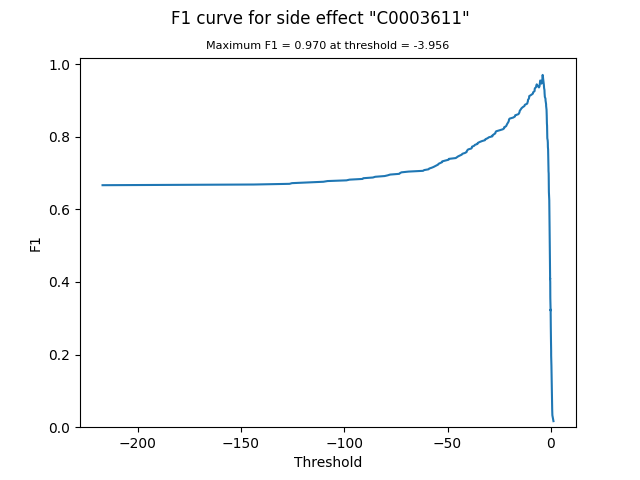

Supplement: btae706_Supplementary_Data [file btae706_supplementary_data.zip › simple_selfloops/figures/C0003611/F1_curve.png]

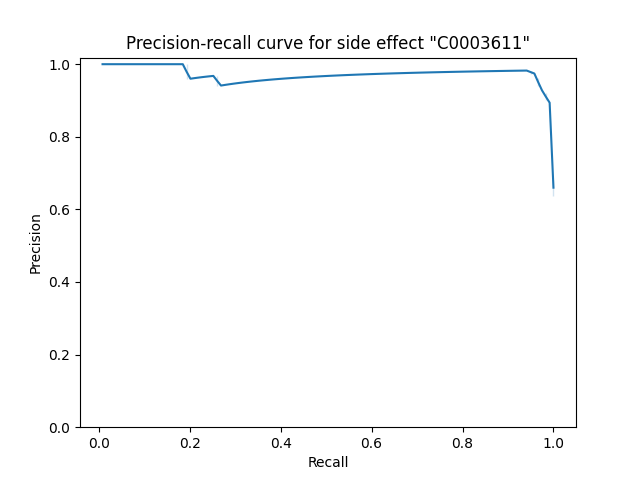

Supplement: btae706_Supplementary_Data [file btae706_supplementary_data.zip › simple_selfloops/figures/C0003611/precision_recall.png]

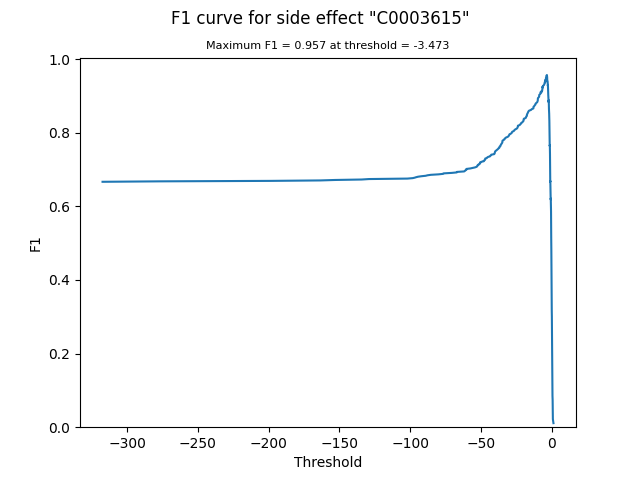

Supplement: btae706_Supplementary_Data [file btae706_supplementary_data.zip › simple_selfloops/figures/C0003615/F1_curve.png]

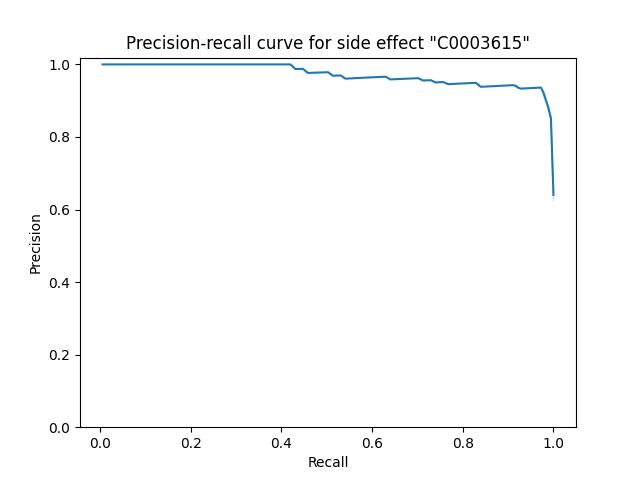

Supplement: btae706_Supplementary_Data [file btae706_supplementary_data.zip › simple_selfloops/figures/C0003615/precision_recall.png]

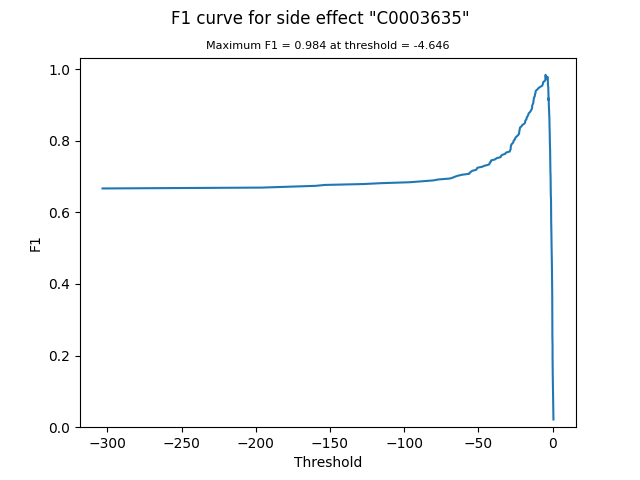

Supplement: btae706_Supplementary_Data [file btae706_supplementary_data.zip › simple_selfloops/figures/C0003635/F1_curve.png]

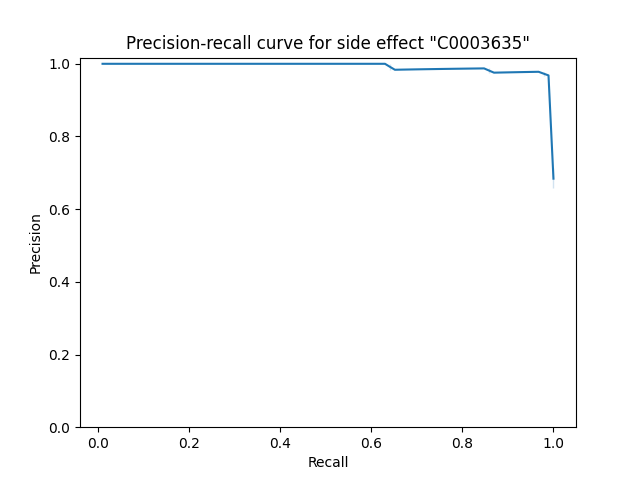

Supplement: btae706_Supplementary_Data [file btae706_supplementary_data.zip › simple_selfloops/figures/C0003635/precision_recall.png]

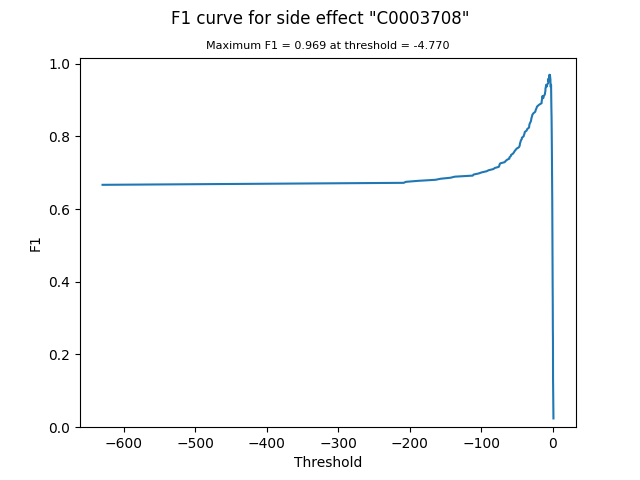

Supplement: btae706_Supplementary_Data [file btae706_supplementary_data.zip › simple_selfloops/figures/C0003708/F1_curve.png]

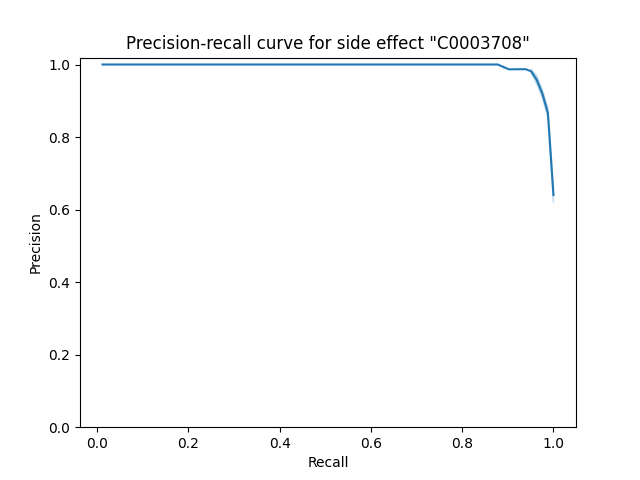

Supplement: btae706_Supplementary_Data [file btae706_supplementary_data.zip › simple_selfloops/figures/C0003708/precision_recall.png]

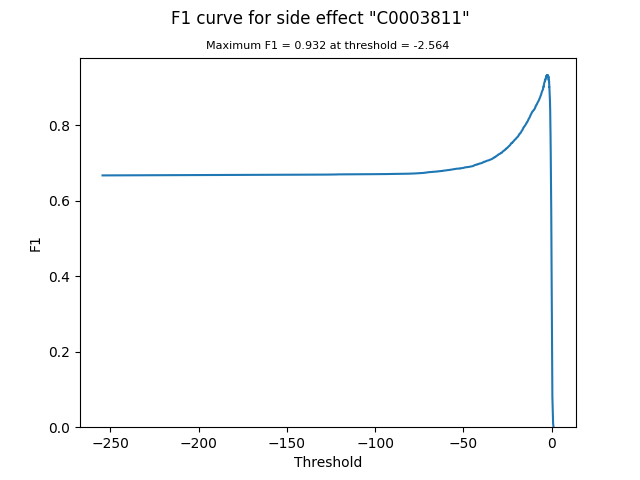

Supplement: btae706_Supplementary_Data [file btae706_supplementary_data.zip › simple_selfloops/figures/C0003811/F1_curve.png]

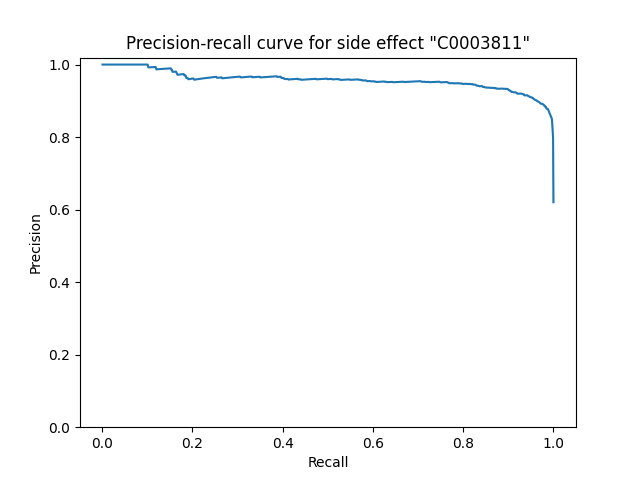

Supplement: btae706_Supplementary_Data [file btae706_supplementary_data.zip › simple_selfloops/figures/C0003811/precision_recall.png]

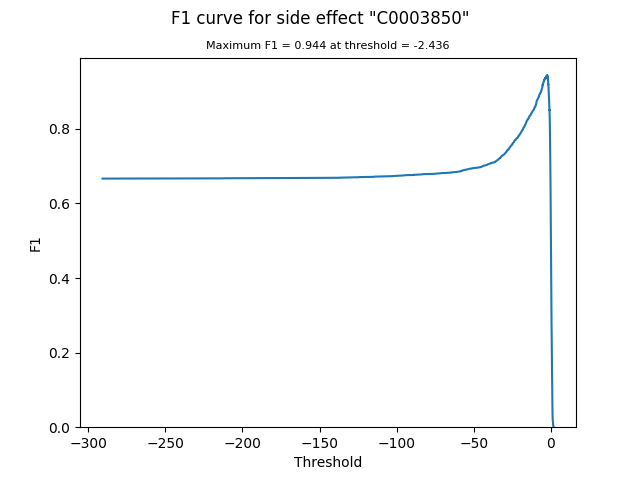

Supplement: btae706_Supplementary_Data [file btae706_supplementary_data.zip › simple_selfloops/figures/C0003850/F1_curve.png]

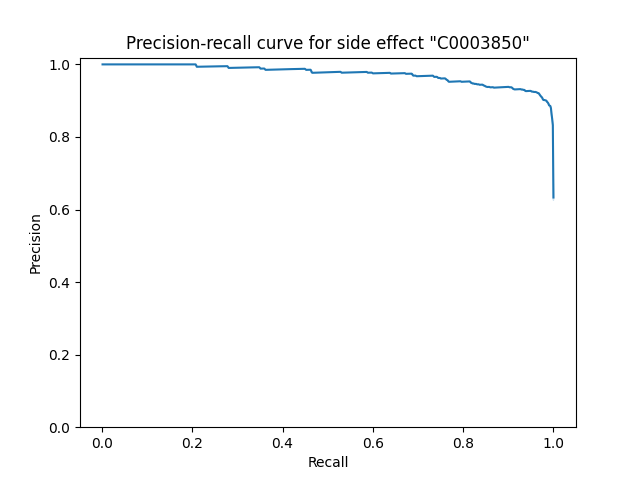

Supplement: btae706_Supplementary_Data [file btae706_supplementary_data.zip › simple_selfloops/figures/C0003850/precision_recall.png]

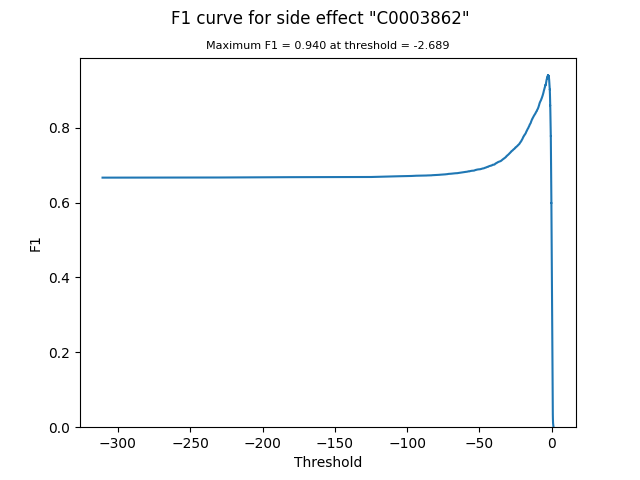

Supplement: btae706_Supplementary_Data [file btae706_supplementary_data.zip › simple_selfloops/figures/C0003862/F1_curve.png]

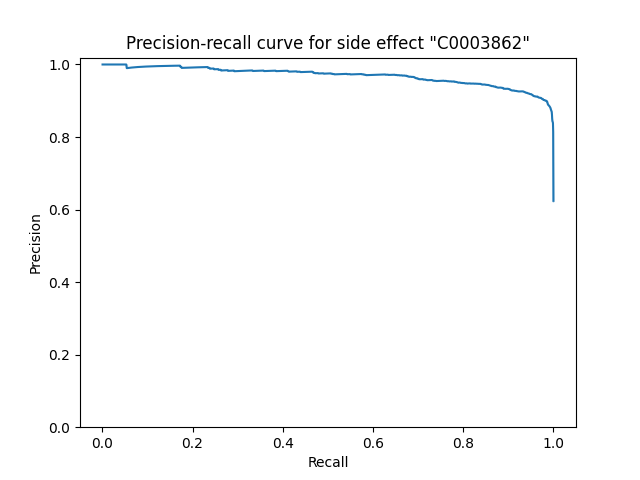

Supplement: btae706_Supplementary_Data [file btae706_supplementary_data.zip › simple_selfloops/figures/C0003862/precision_recall.png]
